# Bench‐Stable *N*‐Heterocyclic Carbene Nickel Precatalysts for C−C and C−N Bond‐Forming Reactions

**DOI:** 10.1002/cctc.201800454

**Published:** 2018-05-02

**Authors:** Felix Strieth‐Kalthoff, Ashley R. Longstreet, Jessica M. Weber, Timothy F. Jamison

**Affiliations:** ^1^ Department of Chemistry Massachusetts Institute of Technology 77 Massachusetts Avenue Cambridge MA 02139 USA; ^2^ Present address: Westfälische Wilhelms-Universität Münster, Organisch-Chemisches Institut Corrensstr. 40 48149 Münster Germany; ^3^ Present address: Department of Chemistry, Biochemistry & Physics The University of Tampa 401 W. Kennedy Blvd. Tampa FL 33606 USA

**Keywords:** homogeneous catalysis, hydroalkenylation, *N*-heterocyclic carbene ligands, nickel, precatalyst

## Abstract

Herein, we introduce a new class of bench‐stable *N*‐heterocyclic carbene (NHC) nickel‐precatalysts for homogeneous nickel‐catalysis. The nickel(II) complexes are readily activated to Ni^0^ in situ under mild conditions, via a proposed Heck‐type mechanism. The precatalysts are shown to facilitate carbonyl‐ene, hydroalkenylation, and amination reactions.

Homogeneous nickel catalysis has evolved into a powerful and versatile tool for organic synthesis.^1^ The particularly attractive properties of nickel extend beyond its low price compared to palladium, and include its ability to undergo facile oxidative addition^2^ and high binding affinity towards unsaturated systems,^3^ along with flexibility in accessing 0 to III oxidation states.1a The development of *N*‐heterocyclic carbene (NHC) ligands^4^ initiated remarkable progress in this field.^5^ Controlled by the electron‐rich, highly‐shielded metal center, Ni−NHC systems have proven effective in a variety of transformations, including challenging cross‐couplings,^6^ cycloadditions,^7^ C−H activation of olefins^8^ and arenes,^9^ and (hydro‐)functionalization of olefins.^10^

The majority of the abovementioned transformations use [Ni(cod)_2_] (cod=1,5‐cyclooctadiene) as the nickel source. Both [Ni(cod)_2_] and free NHCs demonstrate severe sensitivity towards oxygen and moisture and thus require a glovebox for storage and handling. In order to combat this sensitivity, a number of researchers have investigated various strategies^11^ including the formation of stable nickel(II) complexes. Nolan^12^ and Snieckus^13^ independently demonstrated in 2005 the use of Cowley's η^5^‐cyclopentadienyl NHC‐complex **1** 14 (Scheme 1 a) as an effective, bench‐stable Ni‐precatalyst for aryl aminations and Kumada cross‐couplings, respectively. Matsubara also demonstrated the use of NHC‐phosphine Ni‐precatalyst **2** for Kumada cross‐couplings in 2006.^15^ While these precatalysts perform well with reactions such as Suzuki and Kumada cross‐couplings^16^ and hydrosilylations,^17^ reactions are limited when a strong reductant is absent and often require high temperatures. For Ni‐catalyzed aminations, Nicasio demonstrated how mild conditions and a broader substrate scope are obtainable with η^3^‐allyl complex **3**,^18^ presumably by opening up an SN2’ pathway for facile precatalyst activation from Ni^II^ to Ni^0^.^19^ However, the stability of the complex in air was sacrificed for this enhancement.^17^ The Doyle and Monfette groups^20^ reported an air‐stable (TMEDA)Ni(*o*‐tolyl)Cl (TMEDA=tetramethylethylenediamine) precatalyst featuring a labile TMEDA that enables a variety of ligands such as phosphines, diimines and NHCs to be used. While this provides a flexible, modular approach to the formation of the active catalyst, the generality of the precatalyst is limited by its inability to be activated at room temperature.20a

**Scheme 1:**
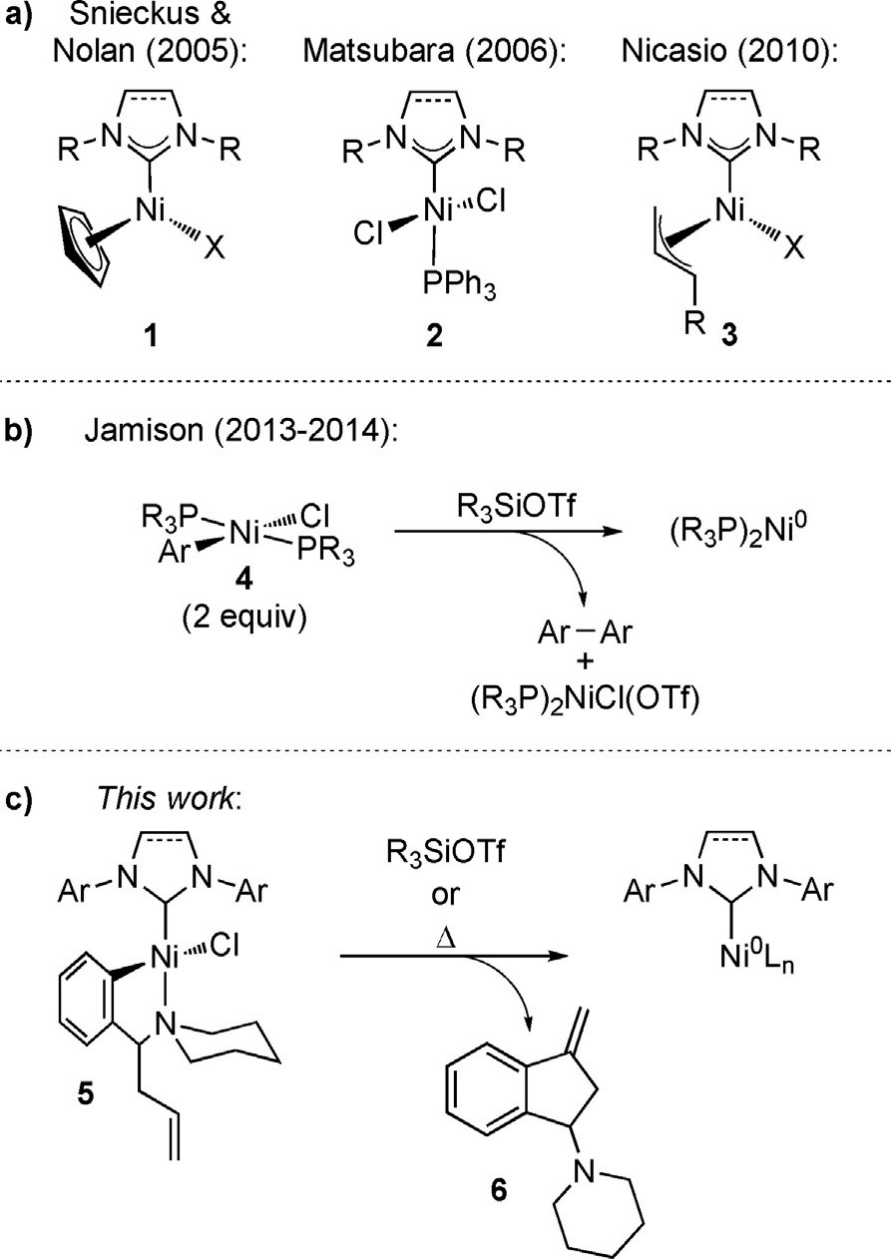
a) Nickel precatalysts bearing an *N*‐heterocyclic carbene ligand.^12^, ^13^, ^15^, ^18^ b) Phosphine Ni‐precatalysts previously developed by our group.^21^.

Our group has previously developed a series of Ni‐precatalysts (**4**) bearing phosphine ligands (Scheme 1 b). The complexes provide facile access to catalytically active Ni^0^ in the presence of silyl triflates.^21^ The reduction of **4** from Ni^II^ to Ni^0^ is suggested to occur by transmetallation with an additional equivalent of **4** followed by reductive elimination, thus only activating 50% of the material (Scheme 1 b).

Herein, we describe a new NHC Ni‐precatalyst design (**5**, Scheme 1 c) that readily reduces to the catalytically active Ni(0) species for reactions such as the Ni‐catalyzed carbonyl‐ene, hydroalkenylation, and amination reactions. This design was inspired by the previous phosphine complexes (**4**) by containing an aryl ligand with the addition of a piperidine moiety to satisfy the coordination sphere. An olefin was also appended to the complex to facilitate the reduction of Ni^II^ to Ni^0^ by an intramolecular Heck reaction.

The investigation began by synthesizing the NHC‐Ni complexes with IPr as the NHC and a bidentate aryl ligand (Table 1). The complexes were each prepared by oxidative addition of the corresponding aryl chloride to a pre‐formed solution of [(IPr)Ni(cod)_2_] in a glovebox. The new complexes were initially synthesized with π‐acceptors *trans* to the strong σ‐donating carbene, such as pyridines (**8**–**10**), an imine (**11**), and a phosphite (**12**), because it was thought necessary for obtaining stable complexes (Table 1, Entries 1–5).^22^ Unexpectedly, stable motifs could also be obtained in the absence of strong π‐acceptors using amine ligands *trans* to the NHC. Whereas acyclic amines did not afford stable complexes (Entry 6), morpholine‐ (**13**), pyrrolidine‐ (**14**), and piperidine‐derived complexes (**15**) could also be prepared in low to moderate yields (Table 1, Entries 7–9). However, complexes **13** and **14** could not be purified to homogeneity. Similar complexes bearing appended olefins with varying carbon linker lengths were also prepared with yields ranging between 38 and 43 % (**5 a**, **16**, **17**, Entries 10–12). The addition of the olefin was made in order to test whether the precatalyst could reduce from Ni^II^ to Ni^0^ by undergoing an intramolecular Heck‐reaction. Interestingly, the bright yellow, cyclic amine complexes (**5 a**, **15**–**17**) demonstrated remarkable stability to both air and column chromatographic conditions with neutral alumina. Lastly, in addition to IPr, complex **5 b** was synthesized with the SIPr ligand (Entry 13).

**Table 1:** Synthesis of novel nickel‐NHC complexes. 
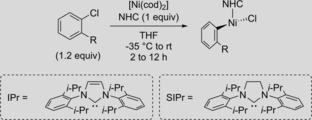

Entry	Ni‐Precatalyst		Isolated
			Yield [%]
1	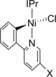	X=H (**8**)	53
2		X=F (**9**)	56
3		X=CF_3_ (**10**)	61
4	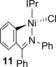		85
5	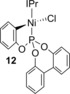		17
6	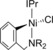	R=Me, Et, Ph	0
7		NR_2_=*N‐*morpholyl (**13**)	29^[a]^
8		NR_2_=*N‐*pyrrolidyl (**14**)	41^[a]^
9		NR_2_=*N‐*piperidyl (**15**)	12
10	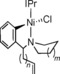	*m*=2, *n*=3 (**16**)	38
11		*m*=1, *n*=1 (**17**)	39
12		*m*=2, *n*=1 (**5 a**)	43
13	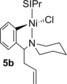		57

[a] Impure complex isolated. See the Supporting Information.

The synthesized complexes along with Cowley's complex (**1 a**) were then evaluated as precatalysts for the IPr‐Ni‐catalyzed carbonyl‐ene reaction between benzaldehyde, 1‐octene, and Et_3_SiOTf (Table 2).^23^ As expected, Cowley's complex **1 a** was not a suitable precatalyst for the carbonyl‐ene reaction (Table 2, Entry 1). When 2‐phenylpyridine (PPy) complex **8** and its electron deficient derivatives (**9** and **10**) were evaluated, very minimal reactivity was observed (Entries 2–4). This may have been caused by catalyst poisoning by the pyridines present. Even when complex **11** with a more bulky imine ligand was employed, no reaction was observed (Entry 5). Complex **12** was then tested for catalytic activity, as cooperative catalysis has been demonstrated for a variety of phosphite‐carbene systems^24^ including the previously mentioned carbonyl‐ene reaction^23^ (Entry 6). Regrettably, **12** also displayed no signs of activity. Complexes with tertiary amines without olefins also displayed little to no reactivity in the carbonyl‐ene reaction (Entries 7–9). However, the yield of the reaction with complex **15** continued to increase to 30 % over seven days. After evaluating a series of complexes with appended olefins with varying linker lengths (Entries 10–12), complex **5 a** (Entry 12) was determined to be an effective precatalyst for the carbonyl‐ene reaction producing **7 a** in 93 % in situ yield after 48 h.

**Table 2:** Evaluation of nickel‐NHC complexes as precatalysts in Ni‐catalyzed carbonyl‐ene reactions. 
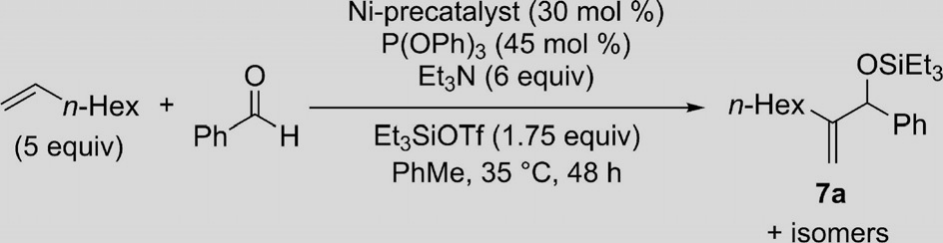

Entry	Ni‐Precatalyst	Yield of **7 a** [%]^[a]^
1	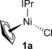	NR
2	**8**	<1
3	**9**	<1
4	**10**	<1
5	**11**	NR
6	**12**	NR
7	**13**	NR
8	**14**	NR
9	**15**	<5, 30^[b]^
10	**16**	NR
11	**17**	<5
***12***	***5 a***	***93***
13	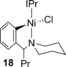	<5

[a] Yields determined by GC against a calibrated internal standard (dodecane, 10 mol %). [b] Reaction time=7 days.

With the success of complex **5 a** as a precatalyst, an investigation into the possible modes of precatalyst activation was undertaken. We hypothesized that by appending an olefin to the NHC‐complex, activation via an intramolecular Heck‐reaction could occur. Indeed, styrene **6** was confirmed to be present by comparing a prepared standard to GC traces and ^1^H NMR spectra of the crude reaction mixtures (Scheme 2). Byproducts that would form by an intermolecular Heck or a mechanism of activation similar to precatalyst **4** were not observed by GC/MS. The necessity of the olefin was further verified by the lack of reactivity observed when complex **18** bearing only the alkyl side chain was used (Table 2, Entry 13).

**Scheme 2:**
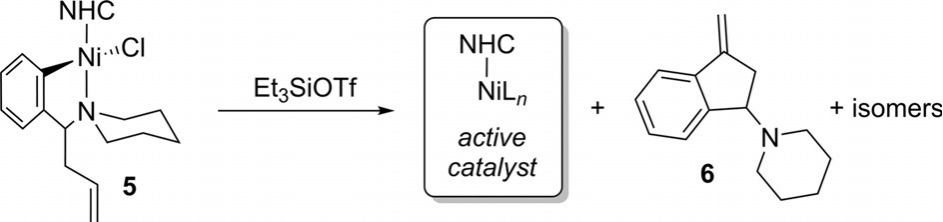
Hypothesized mechanism of activation for precatalyst **5**.

As shown in the product profile for the carbonyl‐ene reaction, precatalyst **5 a** even outperformed the previously reported catalytic system with [Ni(cod)_2_]/IPr by providing an improved turnover number (TON) at identical catalyst loading (Figure 1). After an induction period for ca. 1 h, the reaction with **5 a** produced a catalyst with greater catalytic activity than the reaction with [Ni(cod)_2_]/IPr, which resulted in a higher product yield. Heating the reaction with precatalyst **5 a** to 50 °C did lower the reaction time to 4 h with only a 6–8 % drop in yield (See Supporting Information for details). As an added benefit, no decrease in yield of **7 a** was observed even after precatalyst **5 a** was stored on a benchtop at room temperature for over a month. Because cod was demonstrated to impede reaction yields in earlier reports,^21^ the carbonyl‐ene reaction with precatalyst **5 a** was performed with cod (0.6 equiv) to determine its effect. Indeed, the product yield was suppressed in the presence of cod, with a dramatic 70 % decrease in yield relative to the use of **5 a** without cod at the same 48 h time point.

**Figure 1:**
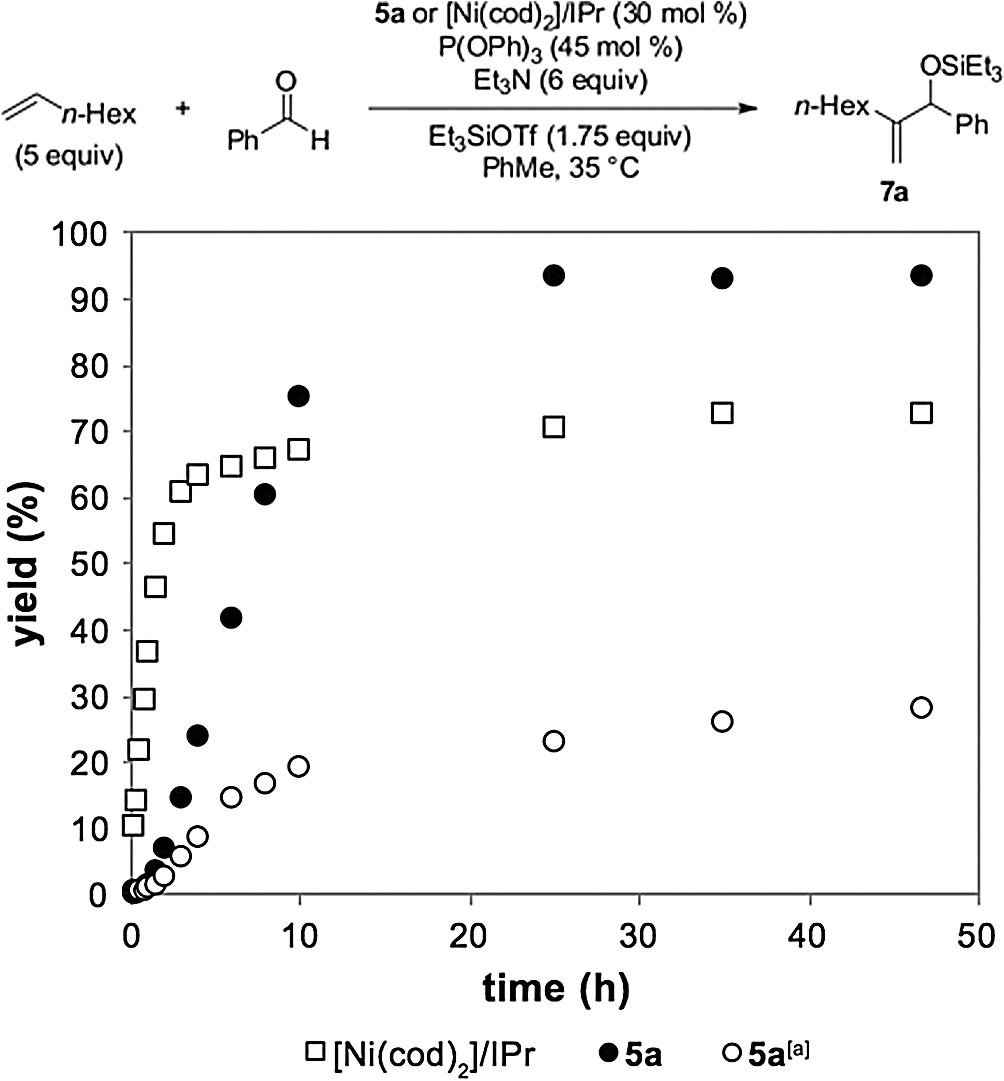
Product profile for the nickel catalyzed carbonyl‐ene reaction with [Ni(cod)_2_]/IPr or **5 a**. Yields determined by GC against a calibrated internal standard (dodecane, 10 mol %). [a] The addition of cod (0.6 equiv) was used.

With these findings in mind, a comparison between precatalyst **5 a** and [Ni(cod)_2_]/IPr with other substrates was performed under otherwise unaltered reaction conditions (Table 3). In all cases, comparable or improved yields with **5 a** were observed. While remarkable improvements in yield for electron deficient aldehydes were observed (Table 3, Entries 4–5), substrates from sterically hindered alkenes still proved to be challenging (Entry 6).

**Table 3:** Reductive, three‐component coupling of aldehydes, α‐olefins and silyl triflates. 
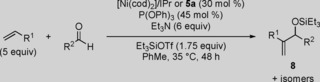

Entry	Product	R^1^	R^2^	Yield of **7** [%]^[a]^	
				[Ni(cod)_2_]/IPr	**5 a**
1	**7 a**	*n*‐hexyl	phenyl	73^[b]^	93^[b]^
2	**7 b**	phenyl	*p‐*anisyl	62	56
3	**7 c**	benzyl	*p‐*anisyl	79	75
4	**7 d**	*n*‐hexyl	2‐furfuryl	10	69
5	**7 e**	*n*‐hexyl	*p‐*chlorophenyl	41	78
6	**7 f**	*t*‐bu	*p‐*anisyl	4	5

[a] Determined by ^1^H NMR against an internal standard (nitromethane). [b] Determined by GC against a calibrated internal standard (dodecane, 10 mol %).

After establishing **5 a** as a valuable precatalyst for the carbonyl‐ene reaction, we next aimed to demonstrate its potential in the Ni‐catalyzed hydrovinylation of α‐olefins (Table 4).^25^ For substrates prepared from styrene (**19 a**) or 2‐vinylnaphthalene (**19 b**), precatalyst **5 a** provided similar or slightly improved yields than the original system (Table 4, Entries 1 and 2). In addition, an electron‐poor substrate demonstrated an improvement in yield by 41 % when **5 a** was employed in place of [Ni(cod)_2_]/IPr (Entry 3).

**Table 4:** Tail‐to‐tail hydrovinylation of olefins with [Ni(cod)_2_]/IPr or precatalyst **5 a**. 
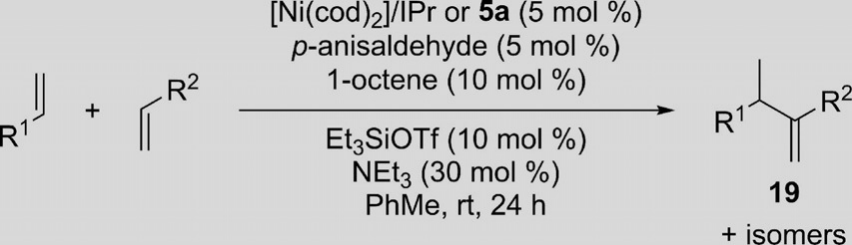

Entry	Product	R^1^	R^2^	Yield of **19** (%)^[a]^	
				[Ni(cod)_2_]/IPr	**5 a**
1	**19 a**	phenyl	*n*‐hexyl	81	79
2	**19 b**	2‐naphthyl	*n*‐hexyl	53	65
3	**19 c** ^[b]^	*p‐*fluoro‐phenyl	*p‐*fluoro‐phenyl	24	65

[a] Isolated yields. [b] The additions of *p‐*anisaldehyde, 1‐octene, and Et_3_SiOTf were doubled.

Lastly, we investigated whether a nickel complex would function as a precatalyst in a reaction that did not involve activation by a silyl triflate. For the Ni‐catalyzed *N*‐arylation of indoles,^26^ the reaction proceeded smoothly with precatalyst **5 a** in lieu of [(IPr)Ni(styrene)_2_] (Scheme 3).

**Scheme 3:**
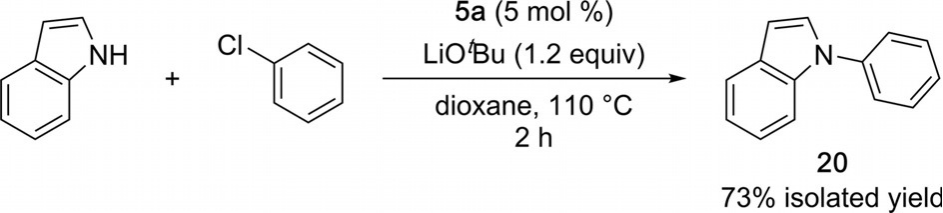
Aryl amination with precatalyst **5 a**.

In summary, we have developed a series of novel bench‐stable precatalysts for homogeneous nickel‐NHC catalysis. These aryl(NHC)nickel(II) chloride complexes with tethered piperidine and olefin moieties were demonstrated to produce the active nickel catalyst in the presence of silyl triflates and under aryl‐amination conditions for the carbonyl‐ene, hydroalkenylation, and amination reactions. While the synthesis of the precatalysts could be further improved to eliminate the need for [Ni(cod)_2_] entirely, the complex **5** has otherwise shown great potential in aiding the continued growth of homogeneous Ni‐catalysis by offering convenience in storage and handling in addition to a catalyst that offers enhanced product yields. The unique tethered‐olefin design has been an inspiration for our group to further develop Ni‐precatalysts beyond NHC ligands and discover new ways to generate Ni^0^ from stable Ni^II^ complexes in situ.

CCDC https://www.ccdc.cam.ac.uk/services/structures?id=doi:10.1002/cctc.201800454 contains the supplementary crystallographic data for this paper.

## Conflict of interest

*The authors declare no conflict of interest*.

## Supporting information

As a service to our authors and readers, this journal provides supporting information supplied by the authors. Such materials are peer reviewed and may be re‐organized for online delivery, but are not copy‐edited or typeset. Technical support issues arising from supporting information (other than missing files) should be addressed to the authors.

Supplementary
