# Supplementary material for: Bench‐Stable N‐Heterocyclic Carbene Nickel Precatalysts for C−C and C−N Bond‐Forming Reactions
Source: ChemCatChem. 2018 May 2;10(13):2873–7. doi: 10.1002/cctc.201800454 (PMC6099443; doi:10.1002/cctc.201800454)

Heterogeneous & Homogeneous & Bio- & Nano-
CHEMCATCHEM
CATALYSIS

# Supporting Information

## Bench-Stable *N*-Heterocyclic Carbene Nickel Precatalysts for C—C and C—N Bond-Forming Reactions

Felix Strieth-Kalthoff,[a, b] Ashley R. Longstreet,[a, c] Jessica M. Weber,[a] and Timothy F. Jamison*[a]

cctc_201800454_sm_miscellaneous_information.pdf

**Table of Contents**

**I. General Information** ........ **3**

**II. Synthesis of NHC-based Nickel Precatalysts** ........ **4**

Pyridine-Derived Nickel-NHC Precatalysts ........ 4

Imine-Derived Nickel-NHC Precatalysts ........ 6

Phosphite-Derived Nickel-NHC Precatalyst ........ 7

Amine-Derived Nickel-NHC Precatalysts ........ 8

Olefin Tethered Amine-Derived Nickel-NHC Precatalysts ........ 10

**III. Carbonyl-Ene Type Coupling of aldehydes and olefins with $R_3SiOTf$** ........ **12**

Temperature Studies ........ 12

**IV. Hydroalkenylation of Olefins** ........ **13**

**V. N-Arylation of Indole** ........ **13**

**VI. Synthesis of Proposed Heck-Activation Product** ........ **14**

**VII. References** ........ **14**

**VIII. Crystallographic Data of Compound 12** ........ **15**

**IX. NMR Spectra** ........ **17**

# I. General Information

Unless otherwise indicated, all reactions were performed in flame- or oven-dried glassware under rigid exclusion of air, either in a nitrogen-filled glove box or using standard Schlenk techniques.

Tetrahydrofuran, dichloromethane, acetonitrile, triethylamine and 1,4-dioxane were degassed by sparging with argon and dried by passing through a column of activated alumina on an SG water solvent purification system. Bis-(1,5-cyclooctadiene)nickel(0) [Ni(cod)$_2$] purchased from Strem Chemicals appeared as yellow crystals and was stored in the glovebox at -30 °C. $PCl_3$ was purified via distillation prior to use. Triethylsilyl triflate (TESOTf) and styrene were distilled over $CaH_2$ prior to use. Liquid olefins were passed through a column of activated $MgSiO_4$ (Florisil) or activated basic alumina and sparged with argon prior to use. 1,3-Bis(2,6-diisopropylphenyl)imidazol-2-ylidene (IPr) was prepared according to a literature procedure[1] and stored in the glove box. Other carbene ligands were used as received from Strem Chemicals.

Thin layer chromatography (TLC) was carried out on EMD Millipore 60 $F_{254}$ glass-backed plates (silica or neutral alumina). Spots were visualized using UV light (254 nm) or a basic potassium permanganate stain. Flash chromatography was carried out on a Biotage Isolera automated column chromatography system using silica (SNAP Ultra), NH-capped silica (SNAP-NH) or alumina (neutral, activated) columns.

Proton ($^1$H), Carbon ($^{13}$C), Fluorine ($^{19}$F) and Phosphorus ($^{31}$P) nuclear magnetic resonance (NMR) spectra were recorded on a Bruker 400 MHz spectrometer. Chemical shifts in $^1$H NMR spectra are reported in parts per million (ppm) relative to residual solvent signals ($CDCl_3$: 7.26 ppm, $CD_2Cl_2$: 5.32 ppm). $^{13}$C chemical shifts are referenced to residual solvent signals ($CDCl_3$: 77.2 ppm, $CD_2Cl_2$: 54.0 ppm). $^{19}$F and $^{31}$P NMR chemical shifts are reported relative to an external standard of $H_3PO_4$ (0.0 ppm) and $CCl_3F$ (0.0 ppm), respectively. Multiplicities are reported as s (singlet), d (doublet), t (triplet), q (quartet), p (quintet), hept (septet). High Resolution Mass Spectra (HRMS) were obtained on a Bruker Daltonics APEXIV 4.7 Tesla Fourier Transform Ion Cyclotron Resonance Mass Spectrometer operating with Electrospray Ionization (ESI) or Direct Analysis in Real Time Ionization (DART). Gas chromatography (GC) was performed with a flame ionization detector. GC yields were determined by calibration using dodecane (20 mol%) as an internal standard.

## II. Synthesis of NHC-based nickel precatalysts

### *Pyridine-derived Nickel-NHC Precatalysts*

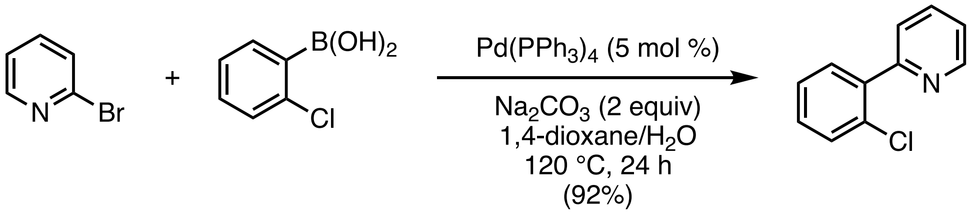

**2-(2-chlorophenyl)pyridine.** Prepared according to the reported procedure with minor modifications.[2] In the glovebox, $Na_2CO_3$ (5.09 g, 48 mmol, 2 equiv), $Pd(Ph_3)_4$ (1.39 g, 1.2 mmol, 0.05 equiv), and 2-chlorophenylboronic acid (4.50 g, 29 mmol, 1.2 equiv) were added to a three-neck round bottom with a stir bar. The flask was equipped with a reflux condenser and sealed with rubber septa. Outside the glovebox, water (10 mL, sparged for >1 h with Ar), 1,4-dioxane (50 mL, from a solvent purification system), and 2-bromopyridine (2.4 mL, 24 mmol, 1 equiv) were added. The reaction was heated to 120 °C for 24 h. Once the reaction was complete, $CH_2Cl_2$ was added and the water was separated from mixture. The organic layer was dried with $MgSO_4$ and concentrated. The product was purified by automated column chromatography ($R_f$ = 0.19, 10% EtOAc, hexanes) to yield 2-(2-chlorophenyl)pyridine as a colorless oil (4.178 g, 92%). $^1H$ NMR (400 MHz, $CDCl_3$): δ 8.74 (m, 1H), 7.76 (td, *J* = 7.6, 1.9 Hz, 1H), 7.70 - 7.64 (m, 1H), 7.61 (dd, *J* = 7.3, 2.1 Hz, 1H), 7.49 (dt, *J* = 7.3, 2.1 Hz, 1H), 7.43 - 7.20 (m, 3H) ppm. $^{13}C\{^1H\}$ NMR (101 MHz, $CDCl_3$): δ 156.9, 149.5, 139.2, 135.9, 132.2, 131.6, 130.1, 129.6, 127.0, 124.9, 122.5 ppm. Spectra in accordance with those described in the literature.[3]

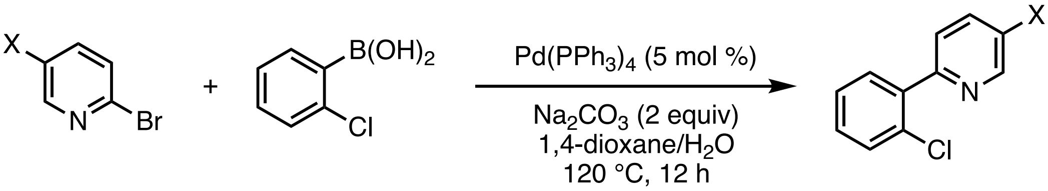

**General procedure:** Prepared according to the reported procedure with minor modifications.[2] A solution of 2-chlorophenylboronic acid (938 mg, 6.0 mmol, 1.2 equiv) in 1,4-dioxane/water (5:1, 10 mL) was added dropwise to a stirred solution of the corresponding 2-bromopyridine (5.0 mmol, 1.0 equiv), sodium carbonate (1.06 g, 10.0 mmol, 2.0 equiv) and $Pd(PPh_3)_4$ (289 mg, 0.25 mmol, 0.05 equiv) in 1,4-dioxane/water (5:1, 10 mL). The mixture was heated under reflux for 12 h. After cooling to room temperature, water (15 mL) was added and the resulting suspension was filtered. The filtrate was extracted with dichloromethane (3 x 15 mL), dried over $MgSO_4$ and concentrated *in vacuo*. The substituted 2-(2-chlorophenyl)pyridines were purified via flash chromatography on silica (10% ethyl acetate/hexanes).

**2-(2-Chlorophenyl)-5-fluoropyridine.** Prepared according to the general procedure using 2-bromo-5-fluoropyridine (880 mg, 5.0 mmol, 1.0 equiv) to obtain 2-(2-chlorophenyl)-5-fluoropyridine as a colorless oil (711 mg, 69%). $^1H$ NMR (400 MHz, $CDCl_3$): δ 8.57 (d, *J* = 2.9 Hz, 1H), 7.67 (dd, *J* = 8.7, 4.4 Hz, 1H), 7.61 – 7.54 (m, 1H), 7.48 (m, 2H), 7.35 (m, 2H) ppm. $^{13}C\{^1H\}$ NMR (101 MHz, $CDCl_3$): δ 158.8 (d, *J* = 256.9 Hz), 153.0 (d, *J* = 4.2 Hz), 138.3, 137.8 (d, *J* = 23.7 Hz), 132.2, 131.6, 130.3, 129.9, 127.2, 125.9 (d, *J* = 4.4 Hz), 122.9 (d, *J* = 18.5 Hz) ppm. $^{19}F$ NMR (376 MHz, $CDCl_3$): δ -128.4 ppm. HRMS-ESI (m/z): $[M+H]^+$ calcd. for $C_{11}H_8ClFN$, 208.0323; found, 208.0321.

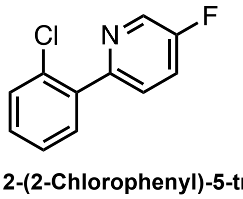

**2-(2-Chlorophenyl)-5-trifluoromethylpyridine.** Prepared according to the general procedure using 2-bromo-5-trifluoromethylpyridine (1.13 g, 5.0 mmol, 1.0 equiv) to obtain 2-(2-chlorophenyl)-5-trifluoromethylpyridine as a colorless oil (961 mg, 75%). $^1H$ NMR (400 MHz, $CDCl_3$): δ 8.99 (m, 1.0 Hz, 1H), 8.01 (m, 1H), 7.82 (m, 1H), 7.66 – 7.58 (m, 1H), 7.54 – 7.47 (m, 1H), 7.44 – 7.36 (m, 2H) ppm. $^{13}C\{^1H\}$ NMR (101 MHz, $CD_2Cl_2$): δ 160.3, 146.6 (q, *J* = 4.1 Hz), 138.0, 133.2 (q, *J* = 3.4 Hz), 132.3, 131.7, 130.5, 130.4, 127.3, 125.4 (q, *J* = 33.2 Hz), 124.8, 123.7 (q, *J* = 273.3 Hz) ppm. $^{19}F$ NMR (376 MHz, $CDCl_3$): δ -62.32 ppm. HRMS-DART (m/z): $[M+H]^+$ calcd. for $C_{12}H_8ClF_3N$, 258.0292; found, 258.0297.

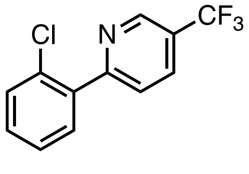

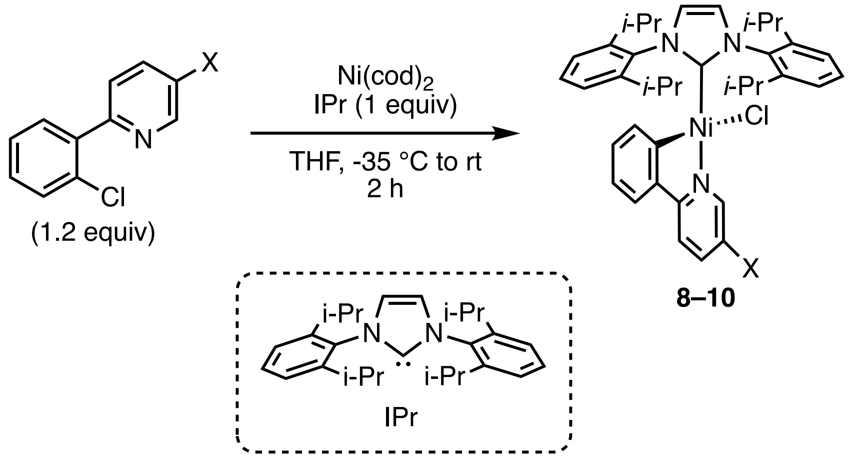


**General procedure:** In the glovebox, a solution of the substituted 2-(2-chlorophenyl)pyridine (0.6 mmol, 1.2 equiv) in tetrahydrofuran (3 mL) was added to a slurry of $Ni(cod)_2$ (138 mg, 0.5 mmol, 1.0 equiv) in tetrahydrofuran (8 mL) at -35 °C. After stirring for 1 min, a solution of IPr (189 mg, 0.5 mmol, 1.0 equiv) was added and the mixture was allowed to reach room temperature and stirred for 2 h. The vial was opened to air and stirred for an addition 10 min. The solution was filtered through celite, and the filtrate was concentrated *in vacuo*. The red-brown residue was purified by flash chromatography on alumina (100% hexanes, then 0% to 100% $CH_2Cl_2$/hexanes).

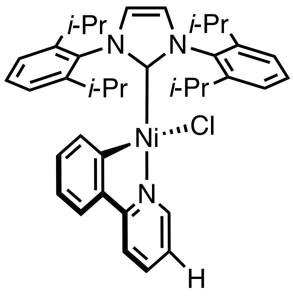

**8**

**Ni(IPr)(2-(pyrid-2-yl)phenyl)Cl (8).** Prepared according to the general procedure using 2-(2-chlorophenyl)pyridine (114 mg, 0.6 mmol, 1.2 equiv) to yield **8** as a yellow/orange solid (169 mg, 53%). $^1$H NMR (400 MHz, $CD_2Cl_2$): δ 9.06 – 8.93 (m, 1H), 7.65 – 7.54 (m, 1H), 7.46 (t, *J* = 7.7 Hz, 2H), 7.42 – 7.17 (m, 8H), 6.95 (m, 2H), 6.90 – 6.82 (m, 1H), 6.34 (d, *J* = 7.5 Hz, 1H), 3.48, (hept, *J* = 6.7 Hz, 2H), 3.14 (hept, *J* = 6.6 Hz, 2H), 1.46 (d, *J* = 6.6 Hz, 6H), 1.20 (d, *J* = 6.8 Hz, 6H), 1.06 (d, *J* = 6.8 Hz, 6H), 0.84 (d, *J* = 6.7 Hz, 6H) ppm. $^{13}$C{$^1$H} NMR (101 MHz, $CD_2Cl_2$): δ 179.8, 163.3, 152.5, 150.3, 148.3, 147.2, 145.4, 139.4, 137.7, 136.4, 129.6,128.4, 125.5, 124.1, 124.0, 123.3, 121.2, 121.0, 116.4, 28.9, 28.5, 26.1, 25.7, 22.9, 22.8 ppm. Anal. Calcd. for $C_{38}H_{44}ClN_3Ni$: C, 71.66; H, 6.96; N, 6.60. Found: C, 71.92; H, 6.90; N, 6.39. HRMS-ESI (m/z): $[M+H]^+$ calcd. for $C_{38}H_{45}ClN_3Ni$, 636.2650; found, 636.2653.

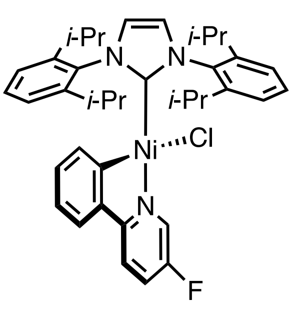

**9**

**Ni(IPr)(2-(5-fluoropyrid-2-yl)phenyl)Cl (9).** Prepared according to the general procedure using 2-(2-chlorophenyl)5-fluoropyridine (125 mg, 0.6 mmol, 1.2 equiv) to yield **9** as an orange solid (184 mg, 56%). $^1$H NMR (400 MHz, $CD_2Cl_2$): δ 8.99 (m, 1H), 7.43 (t, *J* = 7.7 Hz, 2H), 7.33 (m, 4H), 7.27 – 7.21 (m, 4H), 7.12 (m, 1H), 6.92 (m, 1H), 6.83 (m, 1H), 6.30 (d, *J* = 7.5 Hz, 1H), 3.42 (hept, *J* = 6.8 Hz, 2H), 3.07 (hept, *J* = 6.7 Hz, 2H), 1.42 (d, *J* = 6.7 Hz, 6H), 1.16 (d, *J* = 6.8 Hz, 6H), 1.02 (d, *J* = 6.8 Hz, 6H), 0.79 (d, *J* = 6.7 Hz, 6H) ppm. $^{13}$C{$^1$H} NMR (101 MHz, $CD_2Cl_2$): δ 178.9, 160.1 (d, *J* = 3.8 Hz), 158.8, 156.4, 151.3, 148.3, 146.1, 145.4, 139.4, 138.8 (d, *J* = 32.8 Hz), 136.3, 129.6, 128.4, 125.0 (d, *J* = 19.6 Hz), 124.9 (d, *J* = 153 Hz), 124.2, 123.5, 121.3, 117.1 (d, *J* = 5.6 Hz), 28.9, 28.5, 26.1, 25.7, 22.9, 22.8 ppm. $^{19}$F NMR (376 MHz, $CD_2Cl_2$): δ -129.0 ppm. Anal. Calcd. for $C_{38}H_{43}ClN_3Ni$: C, 71.66; H, 6.96; N, 6.60. Found: C, 71.92; H, 6.90; N, 6.39. HRMS-ESI (m/z): $[M-Cl]^+$ calcd. for $C_{38}H_{43}FN_3Ni$, 618.2789; found, 618.2771. MS-ESI (m/z): $[M+H]^+$ calcd. for $C_{38}H_{44}FClN_3Ni$, 654.26; found, 654.26.

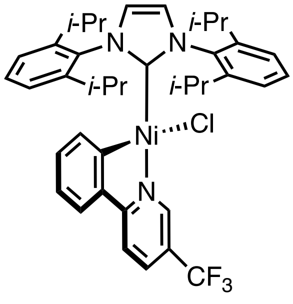

**10**

**Ni(IPr)(2-(5-trifluoromethylpyrid-2-yl)phenyl)Cl (10).** Prepared according to the general procedure using 2-(2-chlorophenyl)5-trifluoromethylpyridine (155 mg, 0.6 mmol, 1.2 equiv) to yield **10** as a red solid (216 mg, 61%). $^1$H NMR (400 MHz, $CD_2Cl_2$): δ 9.39 (m, 1H), 7.78 (m, 1H), 7.49 – 7.40 (m, 3H), 7.33 (m, 2H), 7.29 – 7.21 (m, 5H), 7.04 – 6.89 (m, 2H), 6.38 (m, 1H), 3.42 (hept, *J* = 6.8 Hz, 2H), 3.06 (hept, *J* = 6.7 Hz, 2H), 1.42 (d, *J* = 6.6 Hz, 6H), 1.17 (d, *J* = 6.8 Hz, 6H), 1.02 (d, *J* = 6.8 Hz, 6H), 0.79 (d, *J* = 6.7 Hz, 6H) ppm. $^{13}$C{$^1$H} NMR (101 MHz, $CD_2Cl_2$): δ 178.4, 166.5, 153.9, 148.3, 147.7 (q, *J* = 4.5 Hz), 145.6, 145.4, 139.6, 136.2, 134.7 (q, *J* = 3.3 Hz), 132.74, 129.71, 126.9, 125.7, 124.2, 124.1, 123.7, 123.31 (q, *J* = 271.7 Hz), 122.6, 116.4, 28.9, 28.5, 26.2, 25.7, 22.9, 22.8 ppm. $^{19}$F NMR (376 MHz, $CD_2Cl_2$): δ -62.8 ppm. HRMS-ESI (m/z): $[M-Cl]^+$ calcd. for $C_{39}H_{43}F_3N_3Ni$, 668.2857; found, 668.2767.

***Imine-Derived Nickel-NHC Precatalyst***

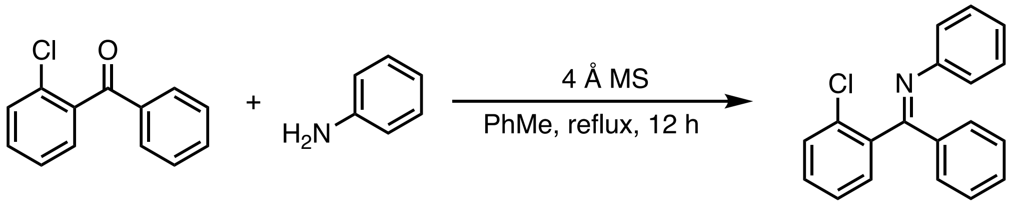


**1-(2-Chlorophenyl)-*N*,1-diphenylmethanimine.** Prepared according to a literature procedure with minor modifications.[4] Aniline (698 mg, 7.50 mmol, 1.50 equiv) was added to a solution of 2-chlorobenzophenone (1.08 g, 5.00 mmol, 1.00 equiv) in toluene (25 mL) over 4 Å molecular sieves. The mixture was heated to reflux and stirred for 20 h. After vacuum filtration and concentration of the filtrate *in vacuo*, the product was purified by flash chromatography (2% ethyl acetate / hexanes) to afford 1-(2-chlorophenyl)-*N*,1-diphenylmethanimine as a light yellow solid (1.12 g, 77%). $^1$H NMR (400 MHz, $CDCl_3$): δ 7.81 – 7.72 (m, 2H), 7.53 – 7.46 (m, 1H), 7.46 – 7.40 (m, 2H), 7.34 (m, 1H), 7.23 (m, 1H), 7.17 (m, 3H), 7.08 (m, 1H), 6.97 – 6.91 (m, 1H), 6.87 – 6.81 (m, 2H) ppm. $^{13}$C{$^1$H} NMR (101 MHz, $CDCl_3$): δ 165.7, 150.9, 138.2, 136.1, 132.5, 131.1, 130.4, 129.9, 129.5, 128.6, 128.5, 126.6, 123.7, 120.2 ppm. HRMS-ESI (m/z): $[M+H]^+$ calcd. for $C_{19}H_{15}ClN$, 292.0888; found, 292.0882.

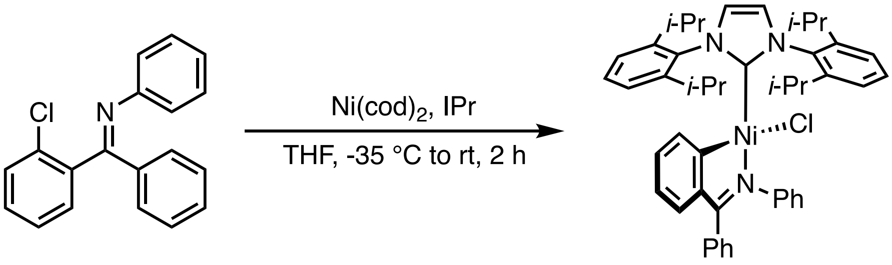


**11**

**Ni(IPr[2-(*N*,1-diphenyliminomethyl)phenyl]Cl.** In the glovebox, a solution of IPr (195 mg, 0.50 mmol, 1.00 equiv) in THF (4 mL) was charged to a slurry of $Ni(cod)_2$ (138 mg, 0.50 mmol, 1.00 equiv) in THF (8 mL) at -35 °C. After stirring for 1 min, 1-(2-chlorophenyl)-*N*,1-diphenylmethanimine (175 mg, 0.60 mmol, 1.20 equiv) in THF (3 mL) was added. The mixture was warmed to room temperature and stirred for 1 h. The vial was then opened to air and the mixture was filtered through celite. After concentration of the filtrate *in vacuo*, purification by flash chromatography (100% hexanes, then 0 to 100% $CH_2Cl_2$) afforded **11** as a red solid (313 mg, 85%). $^1$H NMR (400 MHz, $CD_2Cl_2$): δ 7.56 (t, *J* = 7.7 Hz, 2H), 7.42 (dd, *J* = 7.8, 1.5 Hz, 2H), 7.37 (m, 2H), 7.29 (s, 2H), 7.24 – 7.15 (m, 3H), 6.98 (m, 3H), 6.94 – 6.86 (m, 3H), 6.82 (t, *J* = 7.5 Hz, 1H), 6.65 – 6.50 (m, 4H), 3.51 (hept, *J* = 6.8 Hz, 2H), 3.02 (hept, *J* = 6.7 Hz, 2H), 1.30 (d, *J* = 6.5 Hz, 6H), 1.25 (d, *J* = 6.8 Hz, 6H), 1.01 (d, *J* = 6.9 Hz, 6H), 0.97 (d, *J* = 6.7 Hz, 6H) ppm. $^{13}$C{$^1$H} NMR (101 MHz, $CD_2Cl_2$): δ 180.2, 179.0, 157.3, 150.1, 148.4, 148.2, 145.4, 138.5, 136.5, 134.5, 130.1, 129.6, 128.3, 128.2, 128.2, 127.5, 126.5, 125.4, 125.3, 124.3, 124.1, 124.0, 122.4, 28.9, 28.6, 26.0, 25.8, 23.0, 22.5 ppm. Anal. Calcd. for $C_{46}H_{50}ClN_3Ni$: C, 74.76; H, 6.82; N, 5.57. Found: C, 74.73; H, 6.69; N, 5.57. HRMS-ESI (m/z): $[M-Cl]^+$ calcd. for $C_{46}H_{50}N_3Ni$, 702.3353; found, 702.3347.

### *Phosphite-Derived Nickel-NHC Precatalyst*

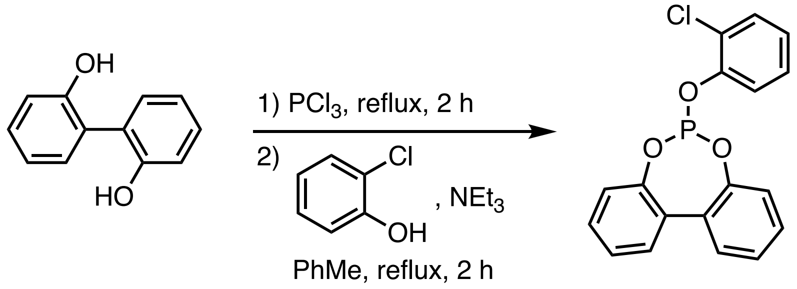


**(2,2'-Biphenyl)(2-chlorophenyl)phosphite.** Prepared according to a literature procedure with minor modifications.[5] 2,2'-Dihydroxybiphenyl (931 mg, 5.00 mmol, 1.00 equiv) was suspended in phosphorus(III)chloride (10.3 g, 75.0 mmol, 15.0 equiv), heated to reflux, and stirred for 2 h. Excess $PCl_3$ was removed by distillation. To remove trace $PCl_3$, the residue was redissolved in toluene (5 mL) and concentrated *in vacuo* three times. To the dried residue was added a solution of 2-chlorophenol (643 mg, 5.00 mmol, 1.00 equiv) in toluene (50 mL). After the addition of triethylamine (608 mg, 6.00 mmol, 1.20 equiv), the mixture was heated to reflux for 2 h. The crude reaction mixture was filtered through a short plug of silica, concentrated *in vacuo* and purified via flash chromatography (10% EtOAc/hexanes) to yield (2,2'-biphenyl)(2-chlorophenyl)phosphite as a white solid (1.13 g, 66%). $^1$H NMR (400 MHz, $CDCl_3$): δ 7.44 (m, 2H), 7.39 (m, 1H), 7.36 – 7.29 (m, 2H), 7.27 – 7.20 (m, 4H), 7.14 (m, 1H), 7.02 (m, 1H) ppm. $^{13}$C{$^1$H} NMR (101 MHz, $CDCl_3$): δ 149.0 (d, *J* = 5.4 Hz), 148.0 (d, *J* = 5.7 Hz), 131.1 (d, *J* = 3.2 Hz), 130.6, 130.1 (d, *J* = 1.2 Hz), 129.3 (d, *J* = 1.0 Hz), 127.8, 126.3 (d, *J* = 3.3 Hz), 125.6 (d, *J* = 1.0 Hz), 125.3 (d, *J* = 1.0 Hz), 122.37 (d, *J* = 9.0 Hz), 122.25 (d, *J* = 1.4 Hz) ppm. $^{31}$P{$^1$H} NMR (162 MHz, $CDCl_3$): δ 142.4 ppm. HRMS-ESI (m/z): [M+H]$^+$ calcd. for $C_{18}H_{13}ClO_3P$, 343.0285; found, 343.0282.

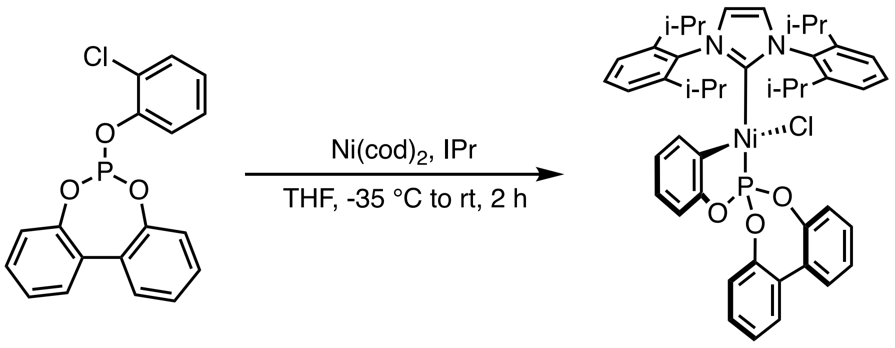


**12**

**Ni(IPr)(η$^2$-(2((2,2'-biphenyl)phosphito)phenyl)Cl (12).** A solution of (2,2'-biphenyl)(2-chlorophenyl)phosphite (206 mg, 0.60 mmol, 1.20 equiv) in THF (3 mL) was charged to a slurry of Ni(cod)$_2$ (138 mg, 0.50 mmol, 1.00 equiv) in THF (7 mL) at -35 °C. After 1 min, a solution of IPr (189 mg, 0.50 mmol, 1.00 equiv) in THF (2 mL) was added and the mixture was warmed room temperature. After stirring for 2 h, the vial was opened to air and stirred for an additional 10 min. The mixture was filtered through celite and concentrated *in vacuo*. Purification by flash chromatography on alumina (100% hexanes, then 0 to 100% $CH_2Cl_2$/hexanes) yielded **12** as a yellow solid (68 mg, 17%). $^1$H NMR (400 MHz, $CD_2Cl_2$): δ 7.52 (m, 2H), 7.45 (m, 2H), 7.39 (d, *J* = 7.7 Hz, 2H), 7.35 – 7.26 (m, 8H), 6.93 (m, 3H), 6.64 (m, 1H), 6.53 (m, 2H), 3.30 (hept, *J* = 6.5 Hz, 2H), 3.05 (hept, *J* = 6.7 Hz, 2H), 1.35 (d, *J* = 6.6 Hz, 6H), 1.19 (d, *J* = 6.8 Hz, 6H), 1.01 (d, *J* = 6.8 Hz, 6H), 0.91 (d, *J* = 6.7 Hz, 6H) ppm. $^{13}$C{$^1$H} NMR (101 MHz, $CD_2Cl_2$): δ 186.5 (d, *J* = 165.4 Hz), 160.1 (d, *J* = 35.2 Hz), 149.3 (d, *J* = 11.6 Hz), 147.8, 145.5, 141.1, 135.9, 133.7, 131.2, 130.9, 130.5, 130.0, 129.9, 129.8, 129.7 (d, *J* = 1.4 Hz), 129.1, 128.9 (d, *J* = 1.5 Hz), 128.6, 126.8, 126.3, 125.8, 125.2 (d, *J* = 4.8 Hz), 124.1 (d, *J* = 4.8 Hz), 122.7 (d, *J* = 3.4 Hz), 122.5 (d, *J* = 2.5 Hz), 110.3 (d, *J* = 17.1 Hz), 28.9, 28.7, 26.2, 26.1, 22.8, 22.6 ppm. $^{31}$P{$^1$H} NMR (162 MHz, $CD_2Cl_2$): δ 142.9 ppm. HRMS-ESI (m/z): [M-Cl]$^+$ calcd. for $C_{45}H_{48}fN_2O_3PNi$, 753.2756; found, 753.2723. MS-ESI (m/z): [M+H]$^+$ calcd. for $C_{45}H_{49}ClN_2O_3PNi$, 789.25; found, 789.27.

***Amine-Derived Nickel-NHC Precatalysts***

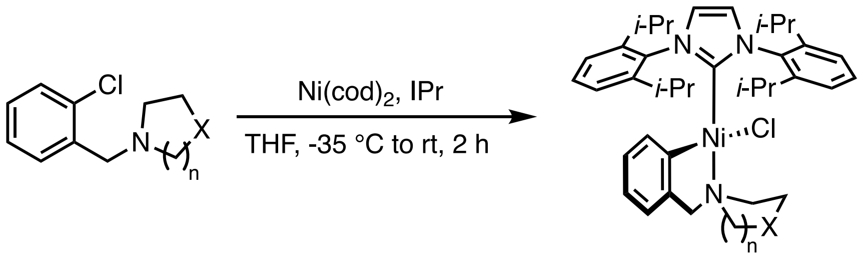


**13–15**

**General procedure:** *N*-(2-chlorobenzyl)amines were prepared from 2-chlorobenzyl chloride and the corresponding amine according to the known literature procedure with spectral data matching those described in the literature.[6] In the glove box, a solution of the *N*-(2-chlorophenyl)amine (0.6 mmol, 1.2 equiv) in THF (3 mL) was charged to a solution of $Ni(cod)_2$ (138 mg, 0.5 mmol, 1.0 equiv) in THF (8 mL) at -35 °C. After stirring for 1 min, a solution of IPr (189 mg, 0.5 mmol, 1.0 equiv) was added and the mixture was warmed to room temperature and stirred for 2 h. The vial was opened to air and stirred for an additional 10 min. The solution was filtered through celite and concentrated *in vacuo*. The red-brown residue was purified by flash chromatography on neutral alumina (100% hexanes, then 0% to 100% $CH_2Cl_2$/hexanes).

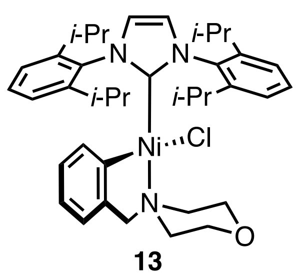


**13**

**Ni(IPr)[2-(*N*-morpholylmethyl)phenyl]Cl (13).** Prepared according to the general procedure using *N*-(2-chlorobenzyl)morpholine (127 mg, 0.6 mmol, 1.2 equiv) to yield **13** as a red solid (96 mg, 29%). Purification was unsuccessful due to product decomposition. $^1$H NMR (400 MHz, $CD_2Cl_2$): δ 7.48 (m, 2H), 7.38 (m, 2H), 7.25 (m, 2H), 7.14 (s, 2H), 6.76 (t, *J* = 7.3 Hz, 1H), 6.69 – 6.54 (m, 2H), 6.09 (d, *J* = 7.6 Hz, 1H), 3.73 – 3.58 (m, 4H), 3.48 – 3.35 (m, 6H), 2.96 (hept, *J* = 6.9 Hz, 2H), 2.15 – 2.05 (m, 2H), 1.42 (d, *J* = 6.6 Hz, 6H), 1.13 (d, *J* = 6.8 Hz, 6H), 1.00 (d, *J* = 6.8 Hz, 6H), 0.77 (d, *J* = 6.7 Hz, 6H) ppm. $^{13}$C{$^1$H} NMR (101 MHz, $CD_2Cl_2$): δ 178.3, 148.8, 148.4, 146.1, 138.8, 137.4, 130.1, 125.8, 124.9, 124.5, 124.5, 123.4, 121.2, 67.5, 64.2, 62.0, 60.2, 54.5, 29.5, 29.0, 26.7, 26.3, 23.2 ppm. HRMS-ESI (m/z): $[M-Cl]^+$ calcd. for $C_{38}H_{50}N_3NiO$, 622.3302; found 622.3313. MS-ESI (m/z): $[M+H]^+$ calcd. for $C_{38}H_{51}ClN_3NiO$, 622.33; found, 622.33.

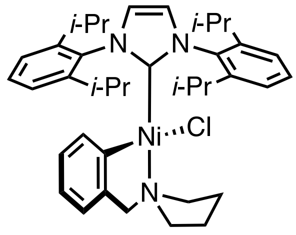


**14**

**Ni(IPr)[2-(*N*-pyrrolidylmethyl)phenyl]Cl (14).** Prepared according to the general procedure using *N*-(2-chlorobenzyl)pyrrolidine (117 mg, 0.6 mmol, 1.2 equiv) to yield **14** as a yellow solid (133 mg, 41%). Purification was unsuccessful due to product decomposition. $^1$H NMR (400 MHz, $CD_2Cl_2$): δ 7.45 (m, 2H), 7.35 (d, *J* = 7.7 Hz, 2H), 7.23 (d, *J* = 7.7 Hz, 2H), 7.13 (s, 2H), 6.71 (t, *J* = 7.3 Hz, 1H), 6.60 (t, *J* = 7.5 Hz, 1H), 6.52 (d, *J* = 7.3 Hz, 1H), 6.14 (d, *J* = 7.5 Hz, 1H), 3.47 (hept, *J* = 7.0 Hz, 2H), 3.27 (m, 4H), 3.04 (hept, *J* = 6.9 Hz, 2H), 2.21 (m, 2H), 1.57 – 1.48 (m, 4H), 1.41 (d, *J* = 6.6 Hz, 6H), 1.14 (d, *J* = 6.8 Hz, 6H), 0.99 (d, *J* = 6.8 Hz, 6H), 0.82 (d, *J* = 6.6 Hz, 6H) ppm. $^{13}$C{$^1$H} NMR (101 MHz, $CD_2Cl_2$): δ 180.6, 150.4, 148.8, 148.0, 145.9, 139.0, 137.6, 129.8, 125.5, 124.9, 124.4, 124.3, 122.9, 120.2, 68.5, 56.7, 29.5, 28.9, 26.56, 26.3, 23.4, 23.3, 22.6 ppm. HRMS-ESI (m/z): $[M-Cl]^+$ calcd. for $C_{38}H_{50}N_3Ni$, 606.3353; found, 606.3360. MS-ESI (m/z): $[M+H]^+$ calcd. for $C_{38}H_{51}ClN_3Ni$, 642.32; found, 642.32.

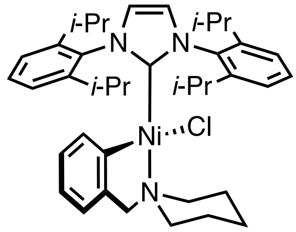


**15**

**Ni(IPr)[2-(*N*-piperidylmethyl)phenyl]Cl (15).** Prepared according to the general procedure using *N*-(2-chlorobenzyl)piperidine (126 mg, 0.6 mmol, 1.2 equiv) to yield **15** as a yellow solid (38 mg, 12%). $^1$H NMR (400 MHz, $CD_2Cl_2$): δ 7.45 (m, 2H), 7.35 (m, 2H), 7.22 (m, 2H), 7.12 (s, 2H), 6.76 – 6.68 (m, 1H), 6.59 (m, 2H), 6.11 (m, 1H), 3.57 – 3.37 (m, 4H), 3.25 (t, *J* = 13.3 Hz, 2H), 2.98 (hept, *J* = 6.8 Hz, 2H), 2.47 (m, 2H), 1.57 – 0.9 (m, 24H), 0.80 (d, *J* = 6.7 Hz, 6H) ppm. $^{13}$C{$^1$H} NMR (101 MHz, $CD_2Cl_2$): δ 179.2, 149.7, 148.2, 147.8, 145.2, 138.3, 137.0, 129.2, 124.9, 124.3, 123.7, 123.6, 122.4, 120.0, 62.3, 52.8, 28.9, 28.4, 26.0, 25.7, 23.7, 22.8, 22.6, 19.4 ppm. HRMS-ESI (m/z): $[M-Cl]^+$ calcd. for $C_{39}H_{52}N_3Ni$: 620.3509; found, 620.3527. ESI-MS (m/z): $[M+H]^+$ calcd. for $C_{39}H_{53}ClN_3Ni$, 656.35; found, 656.35.

***Olefin Tethered Amine-Derived Nickel-NHC Precatalysts***

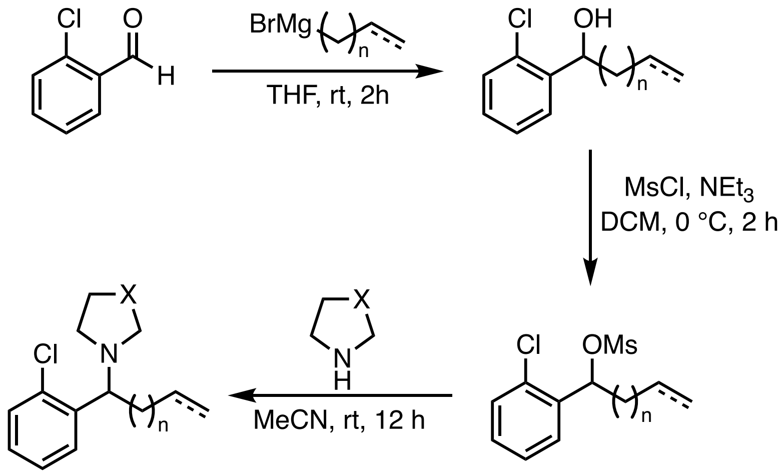

**General procedure:** To a suspension of magnesium powder (1.82 g, 75.0 mmol, 3.0 equiv) in THF (30 mL) was added a small amount of the (n+2)-bromo-1-alkene or 1-bromopropane. After initiation of the Grignard reaction was observed through gentle heating of the solution, the remaining (n+2)-bromo-1-alkene or 1-bromopropane (37.5 mmol, 1.50 equiv) was added dropwise. The solution was then heated to reflux and stirred for 2 h. After cooling to room temperature, the prepared Grignard reagent was added dropwise to a solution of 2-chlorobenzaldehyde (3.51 g, 25.0 mmol, 1.0 equiv) in THF (80 mL). After 2 h, sat. $NH_4Cl$ solution was added slowly until all of the formed magnesium salts had dissolved. The resulting alcohol product was extracted with ethyl acetate (3 x 50 mL), and the combined organic layers were dried over $MgSO_4$, concentrated *in vacuo* and purified by flash chromatography on silica (10% ethyl acetate / hexanes).

To a stirred solution of the respective benzylic alcohol (5.0 mmol, 1.0 equiv) and triethylamine (3.5 mL, 25.0 mmol, 5.0 equiv) in $CH_2Cl_2$ (50 mL) was added methanesulfonyl chloride (475 µL, 6.0 mmol, 1.2 equiv) dropwise at 0 °C. After stirring at 0 °C for 2 h, water (25 mL) was added and the product was extracted with $CH_2Cl_2$ (3 x 25 mL). The combined organic layers were dried over $MgSO_4$ and concentrated *in vacuo*. The respective benzyl mesylate was used without further purification.

The respective amine (25.0 mmol, 5.0 equiv) was added to a solution of the benzyl mesylate in acetonitrile (25 mL). The mixture was stirred at room temperature for 12 h. After concentration *in vacuo*, the residue was purified via flash chromatography on NH-capped silica (100% hexanes, followed by 20% EtOAc/hexanes).

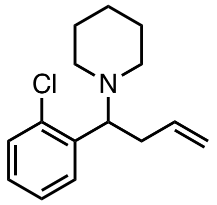

***N*-(1-(2-chlorophenyl)-but-3-en-1-yl)piperadine.** Prepared according to the general procedure to produce *N*-(1-(2-chlorophenyl)-but-3-en-1-yl)piperadine as a light yellow oil (834 mg, 67% yield from the corresponding alcohol). $^1$H NMR (400 MHz, $CDCl_3$): δ 7.44 (m, 1H), 7.33 (m, 1H), 7.23 (m, 1H), 7.14 (m, 1H), 5.63 (m, 1H), 4.95 – 4.86 (m, 2H), 4.05 (m, 1H), 2.63 (m, 1H), 2.51 (m, 3H), 2.37 (m, 2H), 1.53 (m, 4H), 1.39 (m, 2H) ppm. $^{13}$C{$^1$H} NMR (101 MHz, $CDCl_3$): δ 139.1, 135.1, 134.9, 129.5, 127.8, 126.4, 116.8, 104.5, 64.6, 51.6, 37.0, 26.4, 24.8 ppm. HRMS-ESI (m/z): $[M+H]^+$ calcd. for $C_{15}H_{21}ClN$, 250.1357; found, 250.1355.

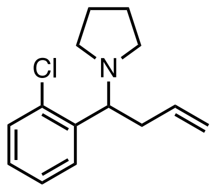

***N*-(1-(2-chlorophenyl)-but-3-en-1-yl)pyrrolidine.** Prepared according to the general procedure to produce *N*-(1-(2-chlorophenyl)-but-3-en-1-yl)pyrrolidine as a yellowish oil (694 mg, 56% yield from the corresponding alcohol). $^1$H NMR (400 MHz, $CDCl_3$): δ 7.54 (d, *J* = 7.8 Hz, 1H), 7.32 (dd, *J* = 8.0, 1.4 Hz, 1H), 7.23 (m, 1H), 7.14 (td, *J* = 7.6, 1.8 Hz, 1H), 5.63 (m, 1H), 4.91 (m, 1H), 4.88 (m, 1H), 3.90 (m, 1H), 2.69 – 2.41 (m, 6H), 1.75 (m, 4H) ppm. $^{13}$C{$^1$H} NMR (101 MHz, $CDCl_3$): δ 140.1, 134.4, 133.6, 129.7, 129.2, 127.7, 126.7, 116.9, 64.8, 52.5, 39.7, 23.3 ppm. HRMS-ESI (m/z): $[M+H]^+$ calcd. for $C_{14}H_{18}ClN$, 236.1201; found, 236.1200.

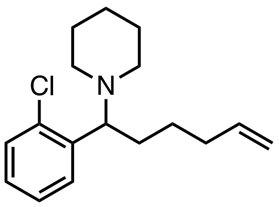

***N*-(1-(2-chlorophenyl)-hex-5-en-1-yl)piperadine.** Prepared according to the general procedure to produce *N*-(1-(2-chlorophenyl)-hex-5-en-1-yl)piperidine as a yellowish oil (1.01 g, 73% yield from the corresponding alcohol). $^1$H NMR (400 MHz, $CDCl_3$): δ 7.41 (dd, *J* = 7.7, 1.8 Hz, 1H), 7.34 (dd, *J* = 7.9, 1.4 Hz, 1H), 7.23 (td, *J* = 7.5, 1.4 Hz, 1H), 7.15 (td, *J* = 7.6, 1.8 Hz, 1H), 5.72 (m, 1H), 4.98 – 4.85 (m, 2H), 3.99 (dd, *J* = 9.0, 4.7 Hz, 1H), 2.47 (m, 2H), 2.33 (m, 2H), 2.00 (m, 2H), 1.88 (m, 1H), 1.71 (m, 1H), 1.52 (m, 4H), 1.37 (m, 2H), 1.32 – 1.21 (m, 1H), 1.15 (m, 1H). $^{13}$C{$^1$H} NMR (101 MHz, $CDCl_3$): δ 139.2, 138.7, 135.0, 129.4, 129.2, 127.6, 126.4, 114.5, 64.4, 51.3, 33.8, 31.9, 26.3, 24.9, 24.7. HRMS-ESI (m/z): $[M+H]^+$ calcd. for $C_{17}H_{25}ClN$, 278.1670; found, 278.1667.

***N*-(1-(2-chlorophenyl)-but-1-yl)piperadine.** Prepared according to the general procedure to produce *N*-(1-(2-chlorophenyl)-but-1-yl)piperidine as a colorless liquid (871 mg, 69% yield from the corresponding alcohol). $^{1}$H NMR (400 MHz, $CDCl_3$): δ 7.44 – 7.10 (m, 4H), 4.00 (m, 1H), 2.41 (m, 4H), 1.83 (m, 1H), 1.69 (m, 1H), 1.62 – 1.43 (m, 4H), 1.37 (m, 2H), 1.12 (m, 2H), 0.84 (t, *J* = 7.3 Hz, 3H) ppm. $^{13}$C{$^{1}$H} NMR (101 MHz, $CDCl_3$): δ 139.4, 135.1, 129.3, 129.2, 127.5, 126.3, 64.4, 51.3, 34.9, 26.4, 24.7, 18.9, 14.3 ppm. HRMS-DART (m/z): $[M+H]^+$ calcd. for $C_{15}H_{23}ClN$, 252.1514; found, 252.1516.

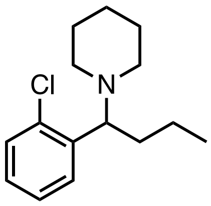

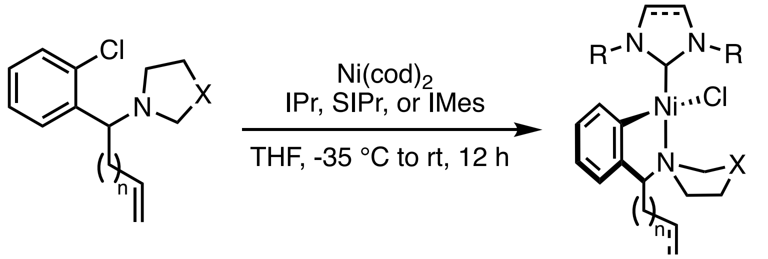


**5, 16–19**

**General procedure**: In the glovebox, a solution of IPr (189 mg, 0.5 mmol, 1.0 equiv) and the ligand precursor (0.6 mmol, 1.2 equiv) in THF (3 mL) was charged to a slurry of $Ni(cod)_2$ (138 mg, 0.5 mmol, 1.0 equiv) in THF (8 mL) at -35 °C. The mixture was warmed room temperature and stirred overnight. The vial was then opened to air and stirred for 10 min. The solution was filtered through celite and concentrated *in vacuo*. The red-brown residue was purified by flash chromatography on alumina (neutral, activated) (100% hexanes, then 0% to 100% $CH_2Cl_2$/hexanes).

**(IPr)[2-(1-(*N*-piperidyl)but-3-en-1-yl)phenyl]nickel(II)chloride (5a).** Prepared according to the general procedure using *N*-(1-(2-chlorophenyl)-but-3-en-1-yl)piperidine (150 mg, 0.60 mmol, 1.2 equiv) to yield **5a** as a yellow solid (149 mg, 43%). $^{1}$H NMR (400 MHz, $CD_2Cl_2$): δ 7.49 – 7.42 (m, 2H), 7.39 – 7.34 (m, 2H), 7.22 (dd, *J* = 7.6, 1.6 Hz, 1H), 7.13 (d, *J* = 2.0 Hz, 1H), 7.07 (d, *J* = 2.0 Hz, 1H), 7.05 (dd, *J* = 7.8, 1.6 Hz, 1H), 6.69 (td, *J* = 7.3, 1.2 Hz, 1H), 6.62 (td, *J* = 7.4, 1.6 Hz, 1H), 6.50 (dd, *J* = 7.2, 1.6 Hz, 1H), 6.17 (dd, *J* = 7.4, 1.2 Hz, 1H), 5.05 (m, 1H), 4.82 – 4.70 (m, 2H), 4.51 (hept, *J* = 7.0 Hz, 1H), 3.80 (hept, *J* = 6.7 Hz, 1H), 3.53 (dd, *J* = 10.5, 4.3 Hz, 1H), 3.31 – 3.19 (m, 1H), 2.86 (td, *J* = 13.3, 3.5 Hz, 1H), 2.80 – 2.71 (m, 1H), 2.65 (hept, *J* = 6.6 Hz, 1H), 2.56 (m, 1H), 2.43 (m, 1H), 2.08 – 1.94 (m, 2H), 1.56 – 0.87 (m, 6H), 1.46 (d, *J* = 6.5 Hz, 3H), 1.34 (d, *J* = 6.7 Hz, 3H), 1.30 (d, *J* = 6.8 Hz, 3H), 1.21 (d, *J* = 6.8 Hz, 3H), 1.12 (d, *J* = 6.8 Hz, 3H), 1.10 (d, *J* = 6.7 Hz, 3H), 0.85 (d, *J* = 7.0 Hz, 3H), 0.25 (d, *J* = 6.6 Hz, 3H) ppm. $^{13}$C{$^{1}$H} NMR (101 MHz, $CD_2Cl_2$): δ 180.1, 153.3, 149.1, 148.9, 148.6, 145.7, 145.5, 139.2, 138.2, 137.3, 137.2, 130.3, 129.6, 126.7, 124.9, 124.8, 124.5, 124.4, 124.3, 123.8, 122.7, 122.3, 116.1, 68.1, 52.4, 50.5, 40.9, 29.4, 29.3, 28.9, 28.8, 27.3, 26.7, 26.4, 25.3, 24.3, 23.8, 23.7, 23.3, 22.9, 20.2, 19.4 ppm. Anal. calcd. for $C_{42}H_{56}ClN_3Ni$: C, 72.37; H, 8.10; N, 6.03. Found: C, 72.66; H, 7.94; N, 5.97. HRMS-ESI (m/z): $[M-Cl]^+$ calcd. for $C_{42}H_{56}N_3Ni$, 660.3822; found, 660.3816. MS-ESI (m/z): $[M]^+$ calcd. for $C_{42}H_{56}ClN_3Ni$, 695.35; found, 695.36

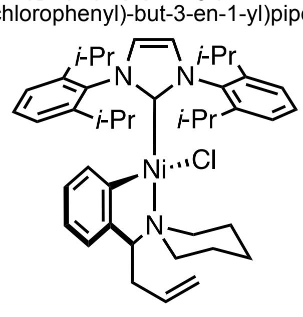


**5a**

**(IPr)[2-(1-(*N*-piperidyl)hex-5-en-1-yl)phenyl]nickel(II)chloride (16).** Prepared according to the general procedure using *N*-(1-(2-chlorophenyl)-hex-5-en-1-yl)piperidine (167 mg, 0.60 mmol, 1.20 equiv) to yield **16** as a yellow solid (139 mg, 38%). $^{1}$H NMR (400 MHz, $CD_2Cl_2$): δ 7.45 (dt, *J* = 16.0, 8.3 Hz, 3H), 7.35 (dd, *J* = 7.7, 1.5 Hz, 1H), 7.21 (dd, *J* = 7.7, 1.5 Hz, 1H), 7.12 – 7.01 (m, 3H), 6.70 (t, *J* = 7.2 Hz, 1H), 6.62 (td, *J* = 7.4, 1.5 Hz, 1H), 6.56 (d, *J* = 7.1 Hz, 1H), 6.14 (d, *J* = 7.4 Hz, 1H), 5.73 (m, 1H), 5.04 – 4.85 (m, 2H), 4.54 (hept, *J* = 6.9 Hz, 1H), 3.79 (hept, *J* = 6.8 Hz, 1H), 3.54 (dd, *J* = 10.8, 4.0 Hz, 1H), 3.29 – 3.14 (m, 1H), 2.86 (td, *J* = 13.2, 3.4 Hz, 1H), 2.78 – 2.52 (m, 3H), 2.00 (hept, *J* = 6.9 Hz, 1H), 1.92 – 1.74 (m, 2H), 1.73 – 1.61 (m, 1H), 1.55 – 1.05 (m, 24H), 0.95 (d, *J* = 14.3 Hz, 1H), 0.85 (m, *J* = 6.9 Hz, 4H), 0.69 (m, 1H), 0.23 (d, *J* = 6.5 Hz, 3H) ppm. $^{13}$C{$^{1}$H} NMR (101 MHz, $CD_2Cl_2$): δ 180.4, 153.8, 149.1, 149.0, 145.8, 145.6, 139.9, 139.3, 138.2, 137.3, 130.4, 129.6, 126.8, 124.8, 124.7, 124.5, 124.4, 124.4, 123.8, 122.5, 122.3, 114.3, 67.3, 52.6, 50.3, 35.2, 34.2, 29.4, 29.3, 28.9, 28.7, 27.1, 26.7, 26.6, 26.4, 25.4, 24.3, 23.8, 23.6, 23.3, 23.0, 20.2, 19.5 ppm. Anal. Calcd. for $C_{44}H_{60}ClN_3Ni$: C, 72.88; H, 8.34; N, 5.70. Found: C, 72.61; H, 8.40; N, 5.79. HRMS-ESI (m/z): $[M-Cl]^+$ calcd. for $C_{44}H_{60}N_3Ni$, 688.4135; found, 688.4147. MS-ESI (m/z): $[M]^+$ calcd. for $C_{44}H_{60}ClN_3Ni$, 723.38; found, 723.40.

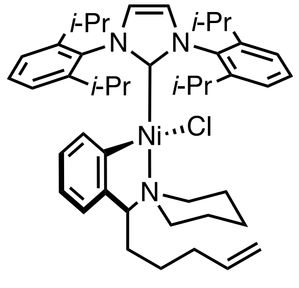


**16**

**(IPr)[2-(1-(*N*-pyrrolidyl)but-3-en-1-yl)phenyl]nickel(II)chloride (17).** Prepared according to the general procedure using *N*-(1-(2-chlorophenyl)but-3-en-1-yl)pyrrolidine (141 mg, 0.60 mmol, 1.2 equiv) to yield **17** as a yellow solid (132 mg, 39%). $^{1}$H NMR (400 MHz, $CD_2Cl_2$): δ 7.49 – 7.42 (m, 2H), 7.41 – 7.34 (m, 2H), 7.25 (dd, *J* = 7.7, 1.6 Hz, 1H), 7.13 – 7.08 (m, 2H), 7.06 (dd, *J* = 7.8, 1.6 Hz, 1H), 6.69 (td, *J* = 7.2, 1.3 Hz, 1H), 6.63 (td, *J* = 7.4, 1.7 Hz, 1H), 6.45 (dd, *J* = 7.1, 1.7 Hz, 1H), 6.19 (dd, *J* = 7.4, 1.3 Hz, 1H), 5.22 – 5.08 (m, 1H), 4.83 – 4.75 (m, 1H), 4.72 (m, 1H), 4.57 (hept, *J* = 6.7 Hz, 1H), 3.73 (hept, *J* = 6.8 Hz, 1H), 3.28 (m, 1H), 3.07 (m, 1H), 2.77 (dd, *J* = 8.9, 5.3 Hz, 1H), 2.69 (p, *J* = 6.7 Hz, 1H), 2.64 – 2.44 (m, 3H), 2.20 – 2.01 (m, 3H), 1.85 – 1.19 (m, 3H), 1.47 (d, *J* = 6.6 Hz, 3H), 1.34 (d, *J* = 6.7 Hz, 3H), 1.28 (d, *J* = 6.8 Hz, 3H), 1.22 (d, *J* = 6.7 Hz, 3H), 1.11 (d, *J* = 6.8 Hz, 3H), 1.09 (d, *J* = 6.8 Hz, 3H), 0.86 (d, *J* = 6.9 Hz, 3H), 0.30 (d, *J* = 6.6 Hz, 3H) ppm. $^{13}$C{$^{1}$H} NMR (101 MHz, $CD_2Cl_2$): δ 180.7, 154.5, 148.9, 148.7, 148.6, 145.8, 145.7, 139.1, 138.1, 137.1, 136.5, 130.4, 129.7, 126.6, 125.0, 124.7, 124.6, 124.6, 124.4, 123.9, 122.3, 121.7, 116.3, 77.5, 59.1, 53.6, 42.7, 29.4, 29.3, 28.9, 28.8,

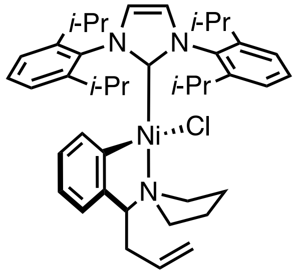


**17**

27.2, 26.7, 26.4, 25.6, 24.1, 23.7, 23.3, 23.2, 23.0, 23.0 ppm. HRMS-ESI (m/z): $[M]^+$ calcd. for $C_{41}H_{54}ClN_3Ni$, 681.3360; found, 681.3373.

**(IPr)[2-(1-(*N*-piperidyl)but-1-yl)phenyl]nickel(II)chloride (18).** Prepared according to the general procedure using *N*-(1-(2-chlorophenylhex-1-yl)piperidine (151 mg, 0.60 mmol, 1.20 equiv) to yield **18** as a yellow solid (77 mg, 22%). $^1$H NMR (400 MHz, $CD_2Cl_2$): δ 7.48 – 7.33 (m, 4H), 7.24 – 7.17 (m, 1H), 7.12 – 7.00 (m, 3H), 6.68 (t, *J* = 7.2 Hz, 1H), 6.61 (dt, *J* = 7.5, 3.8 Hz, 1H), 6.54 (d, *J* = 7.3 Hz, 1H), 6.12 (d, *J* = 7.4 Hz, 1H), 4.53 (hept, *J* = 6.9 Hz, 1H), 3.78 (hept, *J* = 6.9 Hz, 1H), 3.54 (dd, *J* = 10.7, 3.9 Hz, 1H), 3.21 (td, *J* = 14.0, 2.8 Hz, 1H), 2.86 (td, *J* = 13.2, 3.6 Hz, 1H), 2.78 – 2.70 (m, 1H), 2.66 (p, *J* = 6.6 Hz, 1H), 2.56 (d, *J* = 12.9 Hz, 1H), 2.00 (hept, *J* = 6.9 Hz, 1H), 1.58 – 0.58 (m, 34 H), 0.22 (d, *J* = 6.6 Hz, 3H) ppm. $^{13}C\{^1H\}$ NMR (101 MHz, $CD_2Cl_2$): δ 179.95, 153.47, 148.47, 148.36, 148.07, 145.18, 144.97, 138.70, 137.66, 136.71, 129.79, 128.95, 126.13, 124.14, 124.13, 123.86, 123.83, 123.74, 123.19, 121.80, 121.62, 66.75, 52.03, 49.74, 37.16, 28.83, 28.69, 28.34, 28.14, 26.57, 26.08, 25.80, 24.79, 23.77, 23.19, 22.98, 22.66, 22.42, 19.66, 19.63, 18.87, 13.85. HRMS-ESI (m/z): $M^+$ calcd. for $C_{42}H_{58}ClN_3Ni$, 697.3673; found, 697.3645.

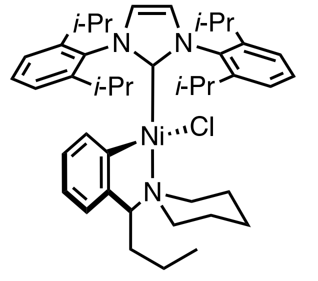


**18**

**(SIPr)[2-(1-(*N*-piperidyl)but-3-en-1-yl)phenyl]nickel(II)chloride (5b).** Prepared according to the general procedure using SIPr (195 mg, 0.50 mmol, 1.0 equiv) instead of IPr and *N*-(1-(2-chlorophenyl)-but-3-en-1-yl)piperidine (150 mg, 0.60 mmol, 1.2 equiv) to yield **5b** as a yellow solid (198 mg, 57%). $^1$H NMR (400 MHz, $CD_2Cl_2$): δ 7.45 (dd, *J* = 7.8, 1.4 Hz, 1H), 7.36 (t, *J* = 7.6 Hz, 1H), 7.29 (t, *J* = 7.8 Hz, 2H), 7.20 (dd, *J* = 7.5, 1.7 Hz, 1H), 7.03 – 6.94 (m, 2H), 6.83 – 6.70 (m, 2H), 6.52 (dd, *J* = 7.1, 1.6 Hz, 1H), 5.08 (m, 1H), 4.83 – 4.62 (m, 3H), 4.29 – 4.13 (m, 1H), 3.91 (m, 4H), 3.50 (dd, *J* = 10.6, 4.2 Hz, 1H), 3.22 (td, *J* = 14.2, 2.7 Hz, 1H), 2.90 (m, 2H), 2.73 (m, 2H), 2.45 (d, *J* = 13.6 Hz, 1H), 2.34 (m, 1H), 1.78 (td, *J* = 11.4, 5.4 Hz, 1H),1.58 – 0.84 (m, 27H), 0.15 (d, *J* = 6.5 Hz, 3H) ppm. $^{13}C\{^1H\}$ NMR (101 MHz, $CD_2Cl_2$): δ 211.5, 153.6, 149.4, 149.3, 148.3, 147.5, 147.2, 139.4, 139.4, 138.1, 137.3, 129.6, 128.6, 125.1, 125.0, 124.9, 124.8, 124.0, 122.8, 122.5, 116.0, 68.1, 56.2, 53.1, 52.3, 50.1, 40.4, 29.5, 29.2, 29.1, 28.7, 27.9, 27.0, 26.4, 26.1, 24.7, 24.3, 24.3, 24.0, 23.3, 20.1, 19.4 ppm. Anal. Calcd. for $C_{42}H_{58}ClN_3Ni$: C, 72.16; H, 8.36; N, 6.01. Found: C, 72.12; H, 8.49; N, 5.98. HRMS-ESI (m/z): $[M+H]^+$ calcd. for $C_{42}H_{59}ClN_3Ni$, 698.3746; found, 698.3736.

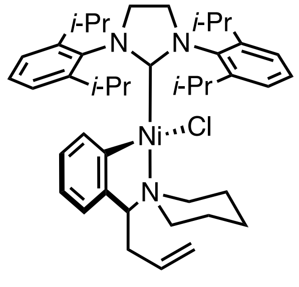


**5b**

## III. Carbonyl-ene type coupling of aldehydes and olefins with $R_3SiOTf$

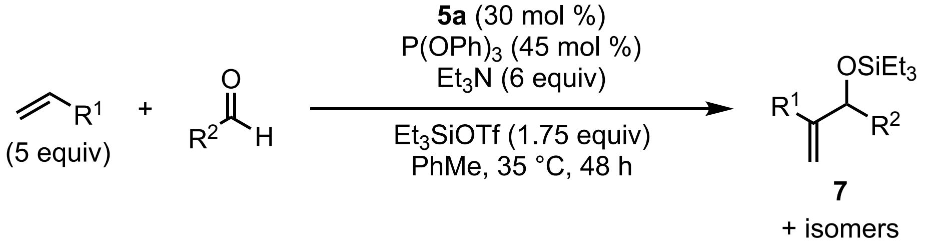


**General Procedure:**[7] In a glovebox, to a stirred solution of **5a** (105 mg, 0.15 mmol, 0.30 equiv) or $Ni(cod)_2$ (41 mg, 0.15 mmol, 0.30 equiv) and IPr (58 mg, 0.15 mmol, 0.30 equiv) in toluene (6 mL) triethylamine (304 mg, 3.00 mmol, 6.00 equiv), triphenylphosphite (106 mg, 0.23 mmol, 0.45 equiv), the respective alkene (2.50 mmol, 5.00 equiv) and the respective aldehyde (0.50 mmol, 1.00 equiv) were added in that order. Triethylsilyl triflate (232 mg, 0.88 mmol, 1.75 equiv) was added dropwise and the mixture was stirred at 35 °C for 48 h. Diethyl ether (10 mL) was added and the reaction was stirred in open air for 30 min. After filtration through a short plug of silica, the crude residue was purified by flash chromatography on silica (1% EtOAc/hexanes).

For some substrates, co-elution of the product with triphenylphosphite or degradation was observed. In these cases, the crude residue was treated with 3 equiv of 1 M TBAF in THF, and the respective alcohol was purified via column chromatography on silica (15% ethyl acetate/hexanes or 80% $CH_2Cl_2$/hexanes).

***O*-Triethylsilyl-2-methylene-1-phenyloctan-1-ol (7a).** Prepared according to the general procedure on a 0.5-fold scale, using benzaldehyde (27 mg, 0.25 mmol, 1.00 equiv) and 1-octene (140 mg, 1.25 mmol, 5.00 equiv). Purification afforded **7a** as a colorless oil (50 mg, 60%). $^1$H NMR (400 MHz, $CDCl_3$): δ 7.37 – 7.33 (m, 2H), 7.29 (m, 2H), 7.25 – 7.19 (m, 1H), 5.21 (m, 1H), 5.13 (s, 1H), 4.86 (m, 1H), 2.00 – 1.88 (m, 1H), 1.80 – 1.69 (m, 1H), 1.37 – 1.18 (m, 8H), 0.92 (t, *J* = 7.9 Hz, 9H), 0.85 (t, *J* = 6.9 Hz, 3H), 0.63 – 0.54 (m, 6H) ppm. $^{13}C\{^1H\}$ NMR (101 MHz, $CDCl_3$): δ 152.2, 143.7, 128.0, 127.0, 126.5, 109.4, 78.2, 31.9, 30.7, 29.3, 27.8, 22.8, 14.2, 7.0, 5.0 ppm. DART-MS (m/z): $[M+H]^+$ calcd. for $C_{21}H_{36}OSi$, 331.2452; found, 331.2464.

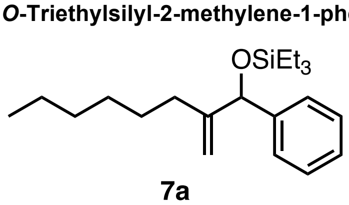


**7a**

***O*-Triethylsilyl-2-benzyl-1-(4-methoxyphenyl)prop-2-en-1-ol (7c).** Prepared according to the general procedure. Characterization data is of the corresponding alcohol. $^1$H NMR (400 MHz, $CDCl_3$): δ 7.31–7.27 (m, 4H), 7.22–7.20 (m, 1H), 7.12 (d, 1H, *J* = 7.1 Hz), 6.90 (d, *J* = 8.6 Hz, 2H), 5.36 (s, 1H), 5.06 (s, 1H), 4.91 (s, 1H), 3.84 (s, 3H), 3.36 (d, 1H, *J* = 15.5 Hz), 3.11 (d, 1H, *J* = 15.5 Hz), 1.81 (brs, 1H) ppm. $^{13}C\{^1H\}$ NMR (101 MHz, $CDCl_3$): δ 159.5, 150.8, 139.3, 134.2, 129.3, 128.5, 128.3, 126.3,

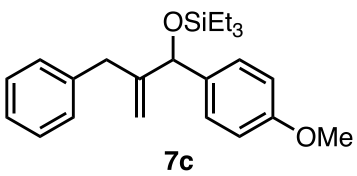


**7c**

114.1, 111.9, 76.1, 55.5, 39.4 ppm. Spectra in accordance with those previously described in the literature.[7]

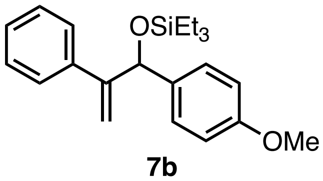

**7b**

***O*-triethyl((1-(4-methoxyphenyl)-2-phenylallyl)oxy)silane (7b).** Prepared according to the general procedure. Characterization data is of the corresponding alcohol. $^{1}$H NMR (400 MHz, $CDCl_3$): δ 7.22 – 7.35 (m, 7H), 6.85 (d, *J* = 8.7 Hz, 2H), 5.68 (s, 1H), 5.53 (s, 2H), 3.79 (s, 3H), 2.04 (s, 1H) ppm. $^{13}$C{$^{1}$H} NMR (101 MHz, $CDCl_3$): δ 159.4, 150.6, 139.7, 134.2, 128.6, 128.5, 127.8, 127.1, 114.1, 113.8, 75.7, 55.5 ppm. Spectra in accordance with those previously described in the literature.[7]

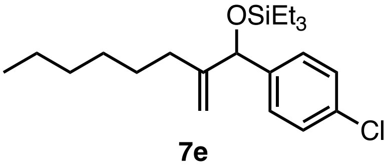

**7e**

***O*-Triethylsilyl-1-(4-chlorophenyl)-2-methyleneoctan-1-ol (7e).** Prepared according to the general procedure. $^{1}$H NMR (400 MHz, $CDCl_3$): δ 7.30 – 7.18 (m, 4H), 5.15 (s, 1.0 Hz, 1H), 5.06 (s, 1H), 4.83 (q, *J* = 1.7 Hz, 1H), 1.95 – 1.82 (m, 1H), 1.74 – 1.62 (m, 1H), 1.39 – 1.08 (m, 8H), 0.88 (t, *J* = 7.9 Hz, 9H), 0.82 (t, *J* = 6.9 Hz, 3H), 0.61 – 0.49 (m, 6H) ppm. $^{13}$C{$^{1}$H} NMR (101 MHz, $CDCl_3$): δ 151.8, 142.3, 132.7, 128.2, 127.8, 109.8, 77.6, 31.9, 30.5, 29.3, 27.8, 22.7, 14.2, 14.2, 7.0, 5.0 ppm. Spectra in accordance with those previously described in the literature.[7]

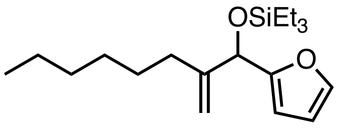

**7d**

***O*-Triethylsilyl-1-(furan-2-yl)-2-methyleneoctan-1-ol (7d).** Prepared according to the general procedure. Characterization data is of the corresponding alcohol. $^{1}$H NMR (400 MHz, $CDCl_3$): δ 7.41 (m, 1H), 6.6 (m, 1H), 6.28 (d, *J* = 3.2 Hz, 1H), 5.27 (s, 1H), 5.19 (d, *J* = 4.2 Hz, 1H), 5.06 (s, 1H), 2.07 – 1.99 (m, 3H), 1.50 – 1.38 (m, 2H), 1.36 – 1.21 (m, 6H), 0.89 (t, *J* = 6.8 Hz, 3H) ppm. $^{13}$C{$^{1}$H} NMR (101 MHz, $CDCl_3$): δ 155.14, 148.98, 142.37, 110.68, 110.41, 107.23, 70.94, 32.45, 31.83, 29.20, 27.98, 22.73, 14.21 ppm. Spectra in accordance with those previously described in the literature.[7]

### *Temperature Studies*

**Figure S1.** Product profile of carbonyl-ene at 35 °C and 50 °C.

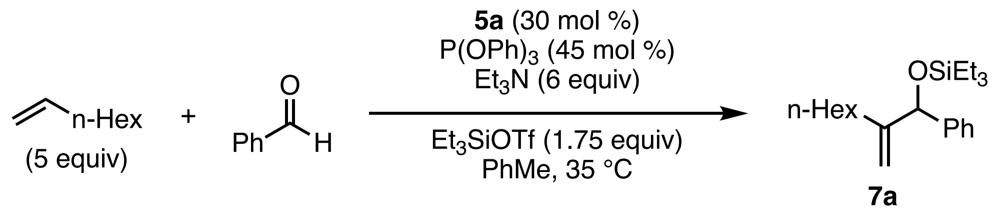


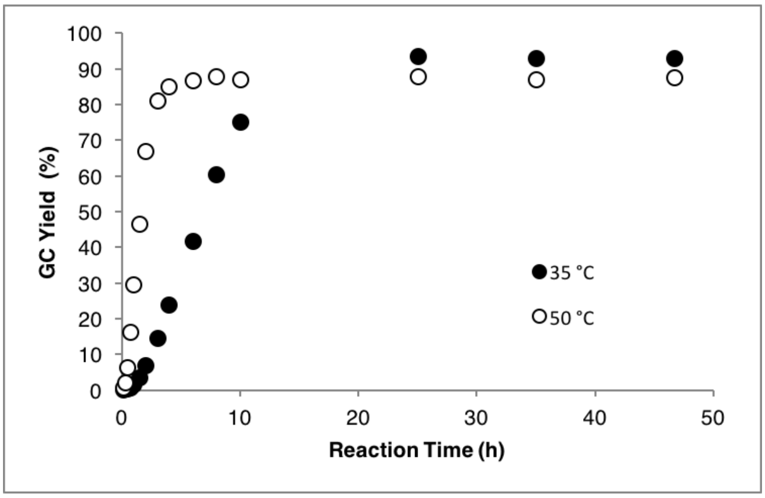

## IV. Hydrovinylation of Olefins

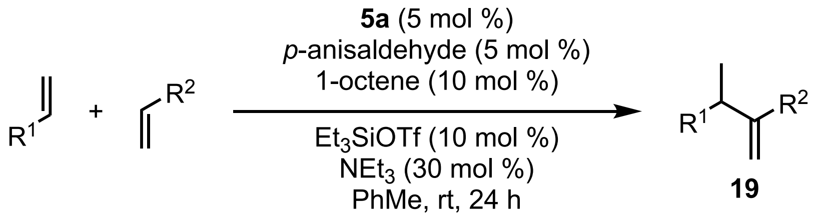


**19**
+ isomers

**General procedure:**[8] To a solution of **5a** (17 mg, 0.03 mmol, 0.05 equiv) or Ni(cod)$_2$ (8 mg, 0.03 mmol, 0.05 equiv) and IPr (12 mg, 0.03 mmol, 0.05 equiv) in toluene (1 mL), *p*-anisaldehyde (3 mg, 0.05 mmol, 0.05 equiv), 1-octene (6 mg, 0.05 mmol, 0.10 equiv), triethylamine (15 mg, 0.15 mmol, 0.30 equiv) and triethylsilyl triflate (13 mg, 0.05 mmol, 0.10 equiv) were added in that order. The mixture was stirred for 15 min, then the vinyl arene (0.50 mmol, 1.00 equiv) and the a-olefin (1.50 mmol, 3.00 equiv) were added and the mixture stirred for another 24 h. The vial was opened to air and the solution was diluted with 4 mL hexanes. After stirring for 30 min, the crude mixture was filtered through silica (washed with 20% EtOAc/hexanes), concentrated *in vacuo* and purified by flash chromatography on silica (1% $CH_2Cl_2$/hexanes).

**3-Methylene-2-phenylnonane (22a).** Prepared according to the general procedure using styrene (52 mg, 0.50 mmol, 1.0 equiv) and 1-octene (168 mg, 1.50 mmol, 3.0 equiv) to yield **22a** as a colorless oil (84 mg, 78%). $^1$H NMR (400 MHz, $CDCl_3$): δ 7.31 (t, *J* = 7.5 Hz, 2H), 7.26 – 7.18 (m, 3H), 4.96 (s, 1H), 4.92 (s, 1H), 3.44 (q, *J* = 7.1 Hz, 1H), 2.02 – 1.87 (m, 2H), 1.40 (d, *J* = 7.1 Hz, 3H), 1.33 – 1.24 (m, 8H), 0.89 (t, *J* = 6.8 Hz, 3H) ppm. $^{13}$C{$^1$H} NMR (101 MHz, $CDCl_3$): δ 153.5, 145.7, 128.4, 127.7, 126.1, 108.6, 45.4, 35.2, 31.9, 29.2, 28.1, 22.8, 20.9, 14.2 ppm. Spectra in accordance with those previously described in the literature.[8]

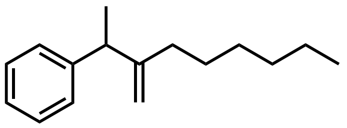

**19a**

**3-Methylene-2-(2-naphthyl)nonane (22b).** Prepared according to the general procedure using 2-vinylnaphthalene (77 mg, 0.50 mmol, 1.0 equiv) and 1-octene (168 mg, 1.50 mmol, 3.0 equiv) to yield **22b** as a colorless oil (86 mg, 65%). $^1$H NMR (400 MHz, $CDCl_3$): δ 7.79 (dd, *J* = 14.7, 8.1 Hz, 3H), 7.66 (s, 1H), 7.50 – 7.39 (m, 2H), 7.35 (dd, *J* = 8.5, 1.8 Hz, 1H), 5.01 (s, 1H), 4.96 (s, 1H), 3.59 (q, *J* = 7.1 Hz, 1H), 2.02 – 1.80 (m, 2H), 1.47 (d, *J* = 7.1 Hz, 3H), 1.41 – 1.19 (m, 8H), 0.85 (t, *J* = 6.8 Hz, 3H) ppm. $^{13}$C{$^1$H} NMR (101 MHz, $CDCl_3$): δ 153.4, 143.2, 133.7, 132.4, 128.0, 127.8, 127.7, 126.5, 125.9, 125.8, 125.3, 109.0, 45.5, 35.2, 31.9, 29.2, 28.1, 22.8, 20.8, 14.2 ppm. Spectra in accordance with those previously described in the literature.[8]

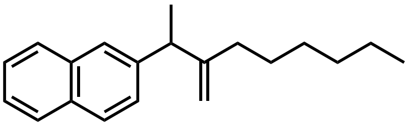

**19b**

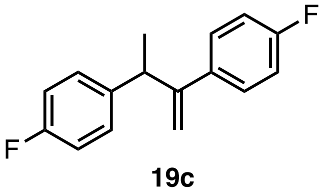


**19c**

**2,3-Bis-(4-fluorophenyl)but-1-ene (22c).** Prepared according to the general procedure using 4-fluorostyrene (122 mg, 1.00 mmol, 2.00 equiv) to yield **22c** as a colorless liquid (79 mg, 65%). $^1$H NMR (400 MHz, $CDCl_3$): δ 7.29 – 7.18 (m, 4H), 6.96 (q, *J* = 8.6 Hz, 4H), 5.43 – 5.35 (m, 1H), 5.20 (d, *J* = 1.3 Hz, 1H), 4.00 (q, *J* = 7.1 Hz, 1H), 1.49 (d, *J* = 7.0 Hz, 3H) ppm. $^{13}$C NMR (101 MHz, $CDCl_3$): δ 162.2 (d, *J* = 246.1 Hz), 161.5 (d, *J* = 244.0 Hz), 151.7, 140.6 (d, *J* = 3.2 Hz), 138.1 (d, *J* = 3.3 Hz), 129.1 (d, *J* = 7.9 Hz), 128.4 (d, *J* = 7.9 Hz), 115.3 (d, *J* = 21.2 Hz), 115.0 (d, *J* = 21.3 Hz), 113.2 (d, *J* = 1.1 Hz), 43.8, 21.8 ppm. Spectra in accordance with those previously described in the literature.[8]

## V. N-Arylation of Indoles

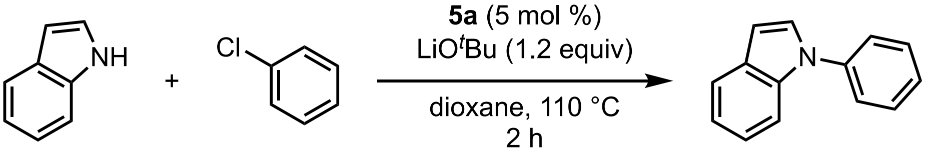


**20**
73% isolated yield

***N*-Phenylindole (23):**[12] Lithium *tert*-butoxide (120 mg, 1.50 mmol, 1.50 equiv) was suspended in a solution of IPr-precatalyst **5a** (35 mg, 0.05 mmol, 0.05 equiv), indole (140 mg, 1.20 mmol, 1.20 equiv) and chlorobenzene (113 mg, 1.00 mmol, 1.00 equiv) in dioxane (1 mL). The mixture was heated under reflux for 2 h. After cooling to room temperature, the reaction mixture was filtered through celite and concentrated in vacuo. Purification of the residue by flash chromatography on silica (100% hexanes) afforded **23** as a white solid (141 mg, 73%). $^1$H NMR (400 MHz, $CDCl_3$) δ 7.67 – 7.61 (m, 1H), 7.51 (dt, *J* = 8.2, 1.0 Hz, 1H), 7.43 (d, *J* = 4.2 Hz, 4H), 7.27 (dd, *J* = 8.5, 3.9 Hz, 2H), 7.20 – 7.09 (m, 2H), 6.63 (dd, *J* = 3.2, 0.8 Hz, 1H) ppm. $^{13}$C NMR (101 MHz, $CDCl_3$) δ 139.9, 136.0, 129.7, 129.4, 128.0, 126.5, 124.5, 122.5, 121.2, 120.5, 110.6, 103.7 ppm.

## VI. Synthesis of Proposed Heck-Activation Product

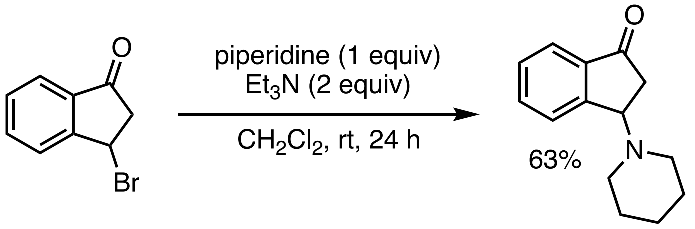


**3-(1-piperidinyl)-1-indanone.** To a stirring solution of 3-bromo-1-indanone (1.48 g, 7 mmol) in $CH_2Cl_2$ (20 mL) was added $Et_3N$ (2.0 mL, 14 mmol, 2 equiv) followed by piperidine (0.7 mL, 7 mmol, 1 equiv). The reaction stirred at room temperature for 24 h. The mixture was then concentrated under vacuum, and the product was purified by automated flash chromatography ($R_f$ = 0.42, 50% EtOAc/hexanes) to afford 3-(1-piperidinyl)-1-indanone as an orange oil (0.95 g, 63% yield). $^1H$ NMR (400 MHz, $CDCl_3$): δ 7.64 (m, 2H), 7.55 (m, 1H), 7.34 (t, *J* = 7.4 Hz, 1H), 4.45 (m, 1H), 2.68 (dd, *J* = 18.9, 3.4 Hz, 1H), 2.53 (dd, *J* = 18.9, 7.1 Hz, 1H), 2.43 – 2.33 (m, 2H), 2.21 (m, 2H), 1.50 (m, 4H), 1.34 (m, 2H). $^{13}C\{^1H\}$ NMR (101 MHz, $CDCl_3$): δ 204.9, 154.9, 137.6, 134.6, 128.6, 126.8, 123.2, 63.4, 49.8, 36.5, 26.3, 24.6. DART-MS (m/z): $[M+H]^+$ calcd. for $C_{14}H_{17}NO$, 216.1383; found, 216.1379.

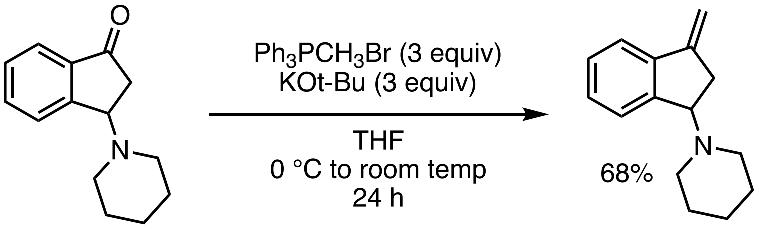


**1-(3-methylene-2,3-dihydro-1*H*-inden-1-yl)piperidine (6).** In the glovebox, methyltriphenylphosphonium bromide (2.1 g, 5.9 mmol, 3 equiv) was added to a round-bottom with THF (8 mL). The round-bottom was placed in the freezer for 30 min. Once cooled, KO*t*-Bu (0.66 g, 5.9 mmol, 3 equiv) was added to the stirring slurry causing the mixture to turn yellow. After stirring for 30 min, a solution of 3-(1-piperidinyl)-1-indanone (0.42 g, 2.0 mmol) in THF (2 mL) was added. The reaction then stirred for 24 h at room temperature. The reaction was then removed from the glovebox and quenched with water (25 mL). The product was first extracted with DCM (3 x 10 mL). The organic solutions were combined, and the product was then extracted with a 0.25 M HCl solution (3 x 10 mL). The aqueous solutions were combined and the pH was adjusted to >10 with NaOH. The product was then extracted again with DCM (3 x 10 mL). The organic layers were combined, dried with $MgSO_4$, and concentrated under vacuum. The product was purified by automated column chromatography using NH-capped silica ($R_f$ = 0.44 with 5% EtOAc/hexanes) to yield **6** as a colorless oil (0.28 g, 68% yield). $^1H$ NMR (400 MHz, $CDCl_3$): δ 7.58 – 7.42 (m, 2H), 7.34 – 7.23 (m, 2H), 5.49 (t, *J* = 2.4 Hz, 1H), 5.07 (t, *J* = 2.1 Hz, 1H), 4.40 (dd, *J* = 7.8, 4.5 Hz, 1H), 2.92 – 2.74 (m, 2H), 2.57 – 2.46 (m, 2H), 2.35 (m, 2H), 1.67 – 1.52 (m, 4H), 1.43 (m, 2H). $^{13}C\{^1H\}$ NMR (101 MHz, $CDCl_3$): δ 148.5, 145.7, 141.4, 128.2, 127.8, 126.2, 120.5, 102.9, 67.6, 49.8, 32.6, 26.5, 24.9. DART-MS (m/z): $[M+H]^+$ calcd. for $C_{15}H_{19}N$, 214.1590; found, 214.1588.

## VII. References

[1] X. Bantreil, S. P. Nolan, *Nat. Protocols* **2011**, *6*, 69-77.
[2] Y. Xiong, J. Wu, S. Xiao, J. Xiao, S. Cao, *J. Org. Chem.* **2013**, *78*, 4599–4603.
[3] T. Nguyen, W. Chiu, X. Wang, M. O. Sattler, J. A. Love, *Org. Lett.* **2016**, *18*, 5492–5495.
[4] D. Song, S. Cho, Y. Han, Y. You, W. Nam, *Org. Lett.* **2013**, *15*, 3582–3585.
[5] J. Li, M. Lutz, A. L. Spek, G. P. M. van Klink, G. van Koten, R. J. M. Klein Gebbink, *J. Organomet. Chem.* **2010**, *695*, 2618–2628.
[6] F. S. Wekesa, N. Phadke, C. Jahier, D. B. Cordes, M. Findlater, *Synthesis* **2014**, *46*, 1046–1051.
[7] C.-Y. Ho, T. F. Jamison, *Angew. Chem., Int. Ed.* **2007**, *46*, 782–785; *Angew. Chem.* **2007**, *119,* 796–799.
[8] C.-Y. Ho, L. He, *Angew. Chem., Int. Ed.* **2010**, *49*, 9182–9186; *Angew. Chem.* **2010**, *122,* 9363–9372.

## VIII. Crystallographic Data for Compound 12

**Figure S2.** Crystal structure of compound **12**.

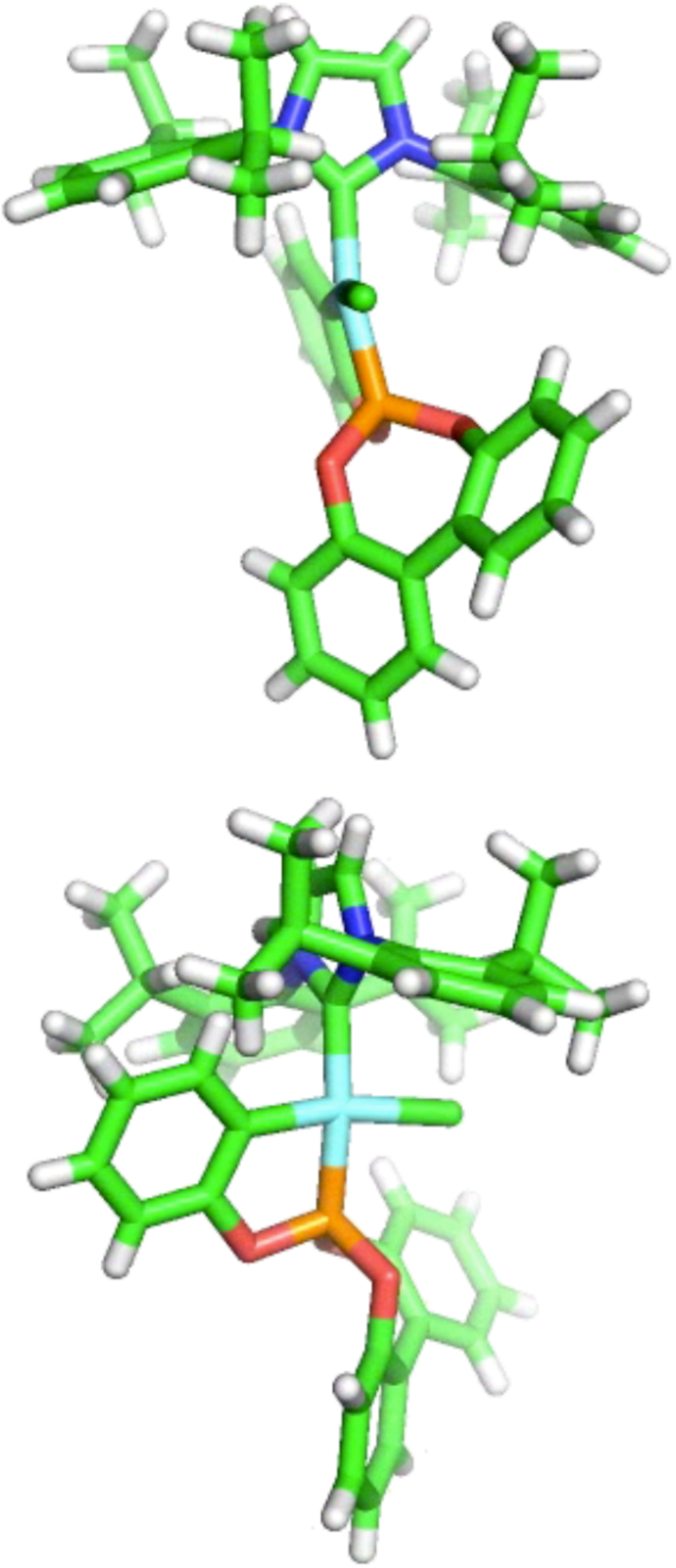

**Table S1**. Crystal data and structure refinement for compound **12**.

| | | |
|---|---|---|
| Identification code | X16219 | |
| Empirical formula | C45 H48 Cl N2 Ni O3 P | |
| Formula weight | 789.98 | |
| Temperature | 100(2) K | |
| Wavelength | 0.71073 Å | |
| Crystal system | Orthorhombic | |
| Space group | Pbca | |
| Unit cell dimensions | a = 13.8741(17) Å | a= 90°. |
| | b = 15.2911(18) Å | b= 90°. |
| | c = 37.543(5) Å | g = 90°. |
| Volume | 7964.8(17) $Å^3$ | |
| Z | 8 | |
| Density (calculated) | 1.318 $Mg/m^3$ | |
| Absorption coefficient | 0.637 $mm^{-1}$ | |
| F(000) | 3328 | |
| Crystal size | 0.198 x 0.144 x 0.007 $mm^3$ | |
| Theta range for data collection | 1.085 to 27.485°. | |
| Index ranges | -18<=h<=18, -19<=k<=19, -48<=l<=48 | |
| Reflections collected | 127259 | |
| Independent reflections | 9116 [R(int) = 0.1163] | |
| Completeness to theta = 25.242° | 100.0 % | |
| Absorption correction | Semi-empirical from equivalents | |
| Max. and min. transmission | 0.7457 and 0.5700 | |
| Refinement method | Full-matrix least-squares on $F^2$ | |
| Data / restraints / parameters | 9116 / 0 / 486 | |
| Goodness-of-fit on $F^2$ | 1.100 | |
| Final R indices [I>2sigma(I)] | R1 = 0.0602, wR2 = 0.1173 | |
| R indices (all data) | R1 = 0.0944, wR2 = 0.1302 | |
| Extinction coefficient | n/a | |
| Largest diff. peak and hole | 0.413 and -0.775 e.$Å^{-3}$ | |

## IX. NMR Spectra

All reported spectra were obtained on a 400 MHz NMR spectrometer.

**Figure S3.** $^{1}H$, $^{13}C$, and $^{19}F$ NMR spectra of 2-(2-chlorophenyl)-5-fluoropyridine in $CDCl_3$.

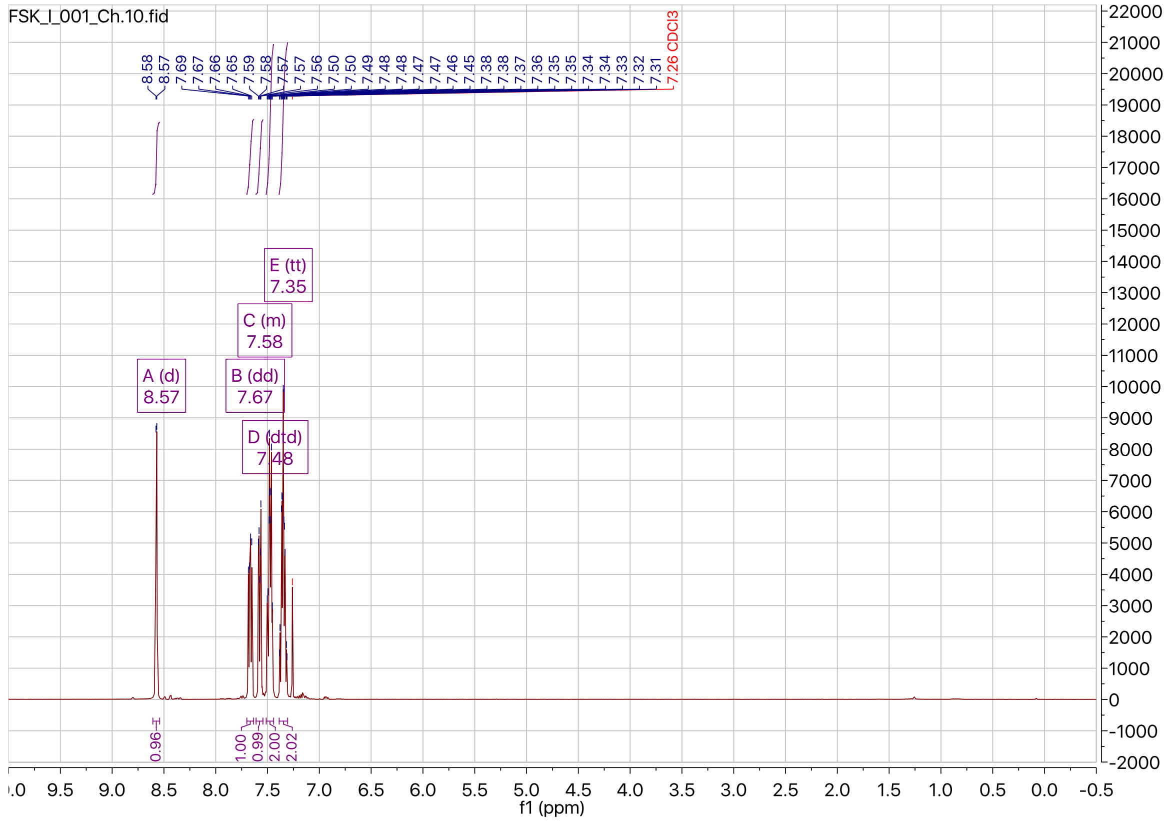

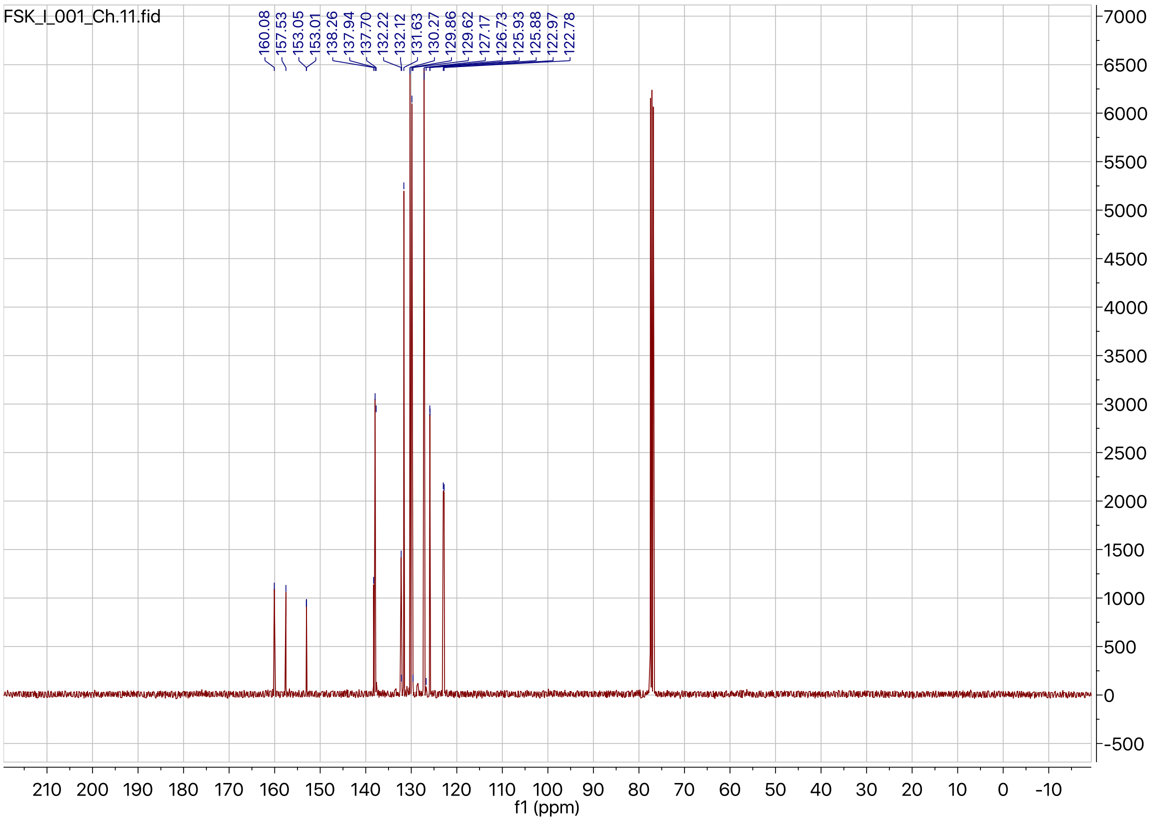
FSK_I_001_Ch.11.fid
160.08
157.53
153.05
153.01
138.26
137.94
137.70
132.22
132.12
131.63
130.27
129.86
129.62
127.17
126.73
125.93
125.88
122.97
122.78
f1 (ppm)

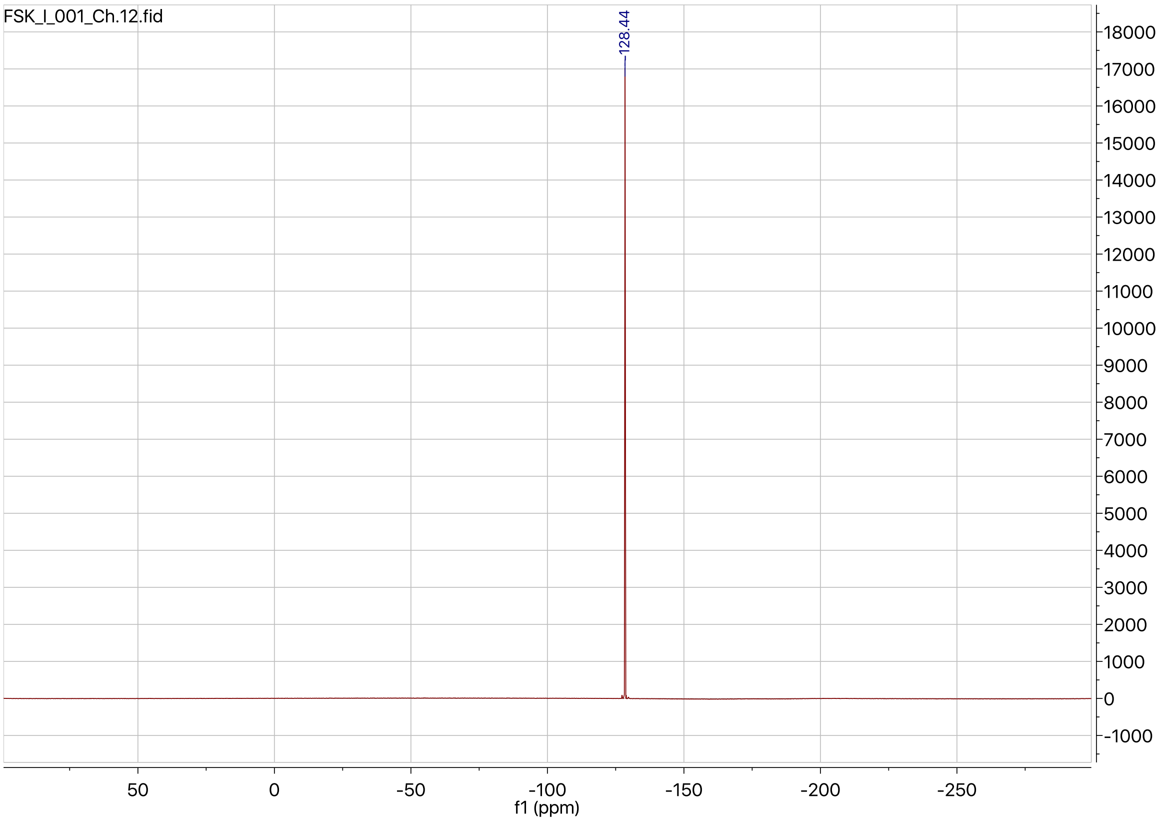
FSK_I_001_Ch.12.fid
128.44
f1 (ppm)

**Figure S4.** $^{1}H$, $^{13}C$, and $^{19}F$ NMR spectra of 2-(2-chlorophenyl)-5-trifluoromethylpyridine in $CDCl_3$.

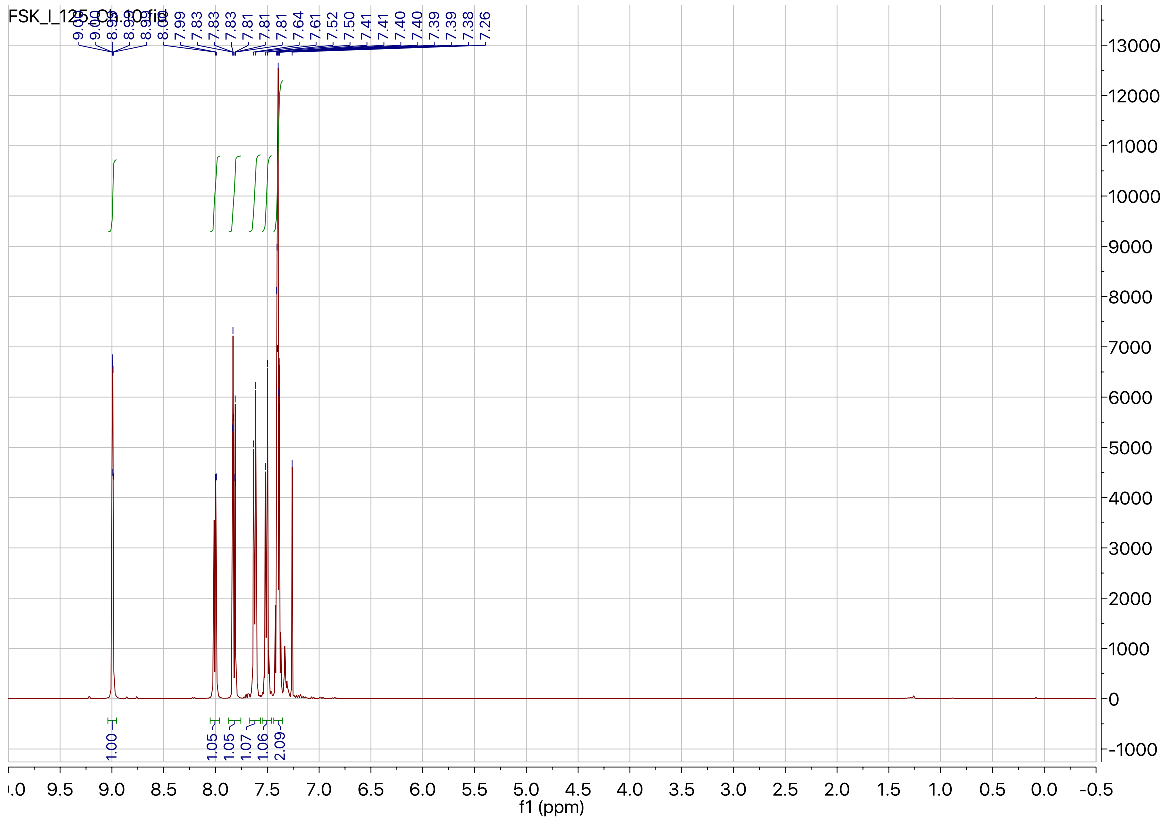


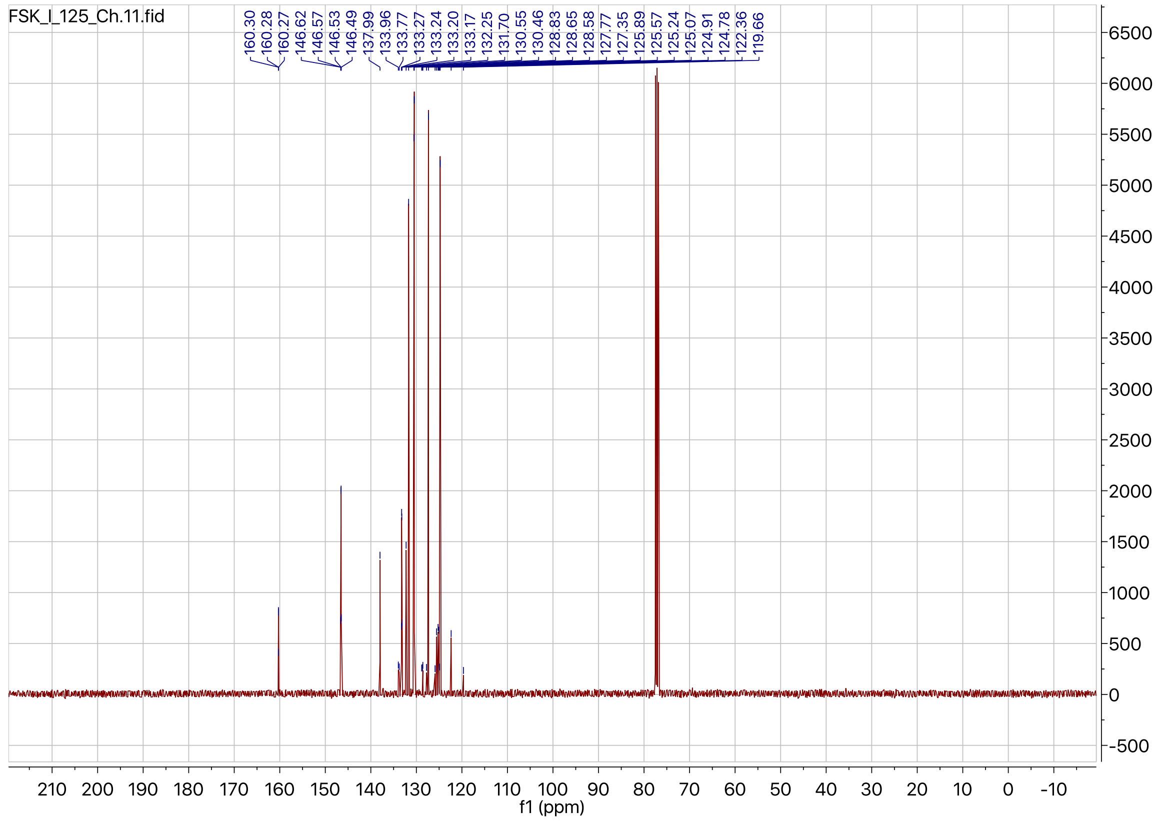

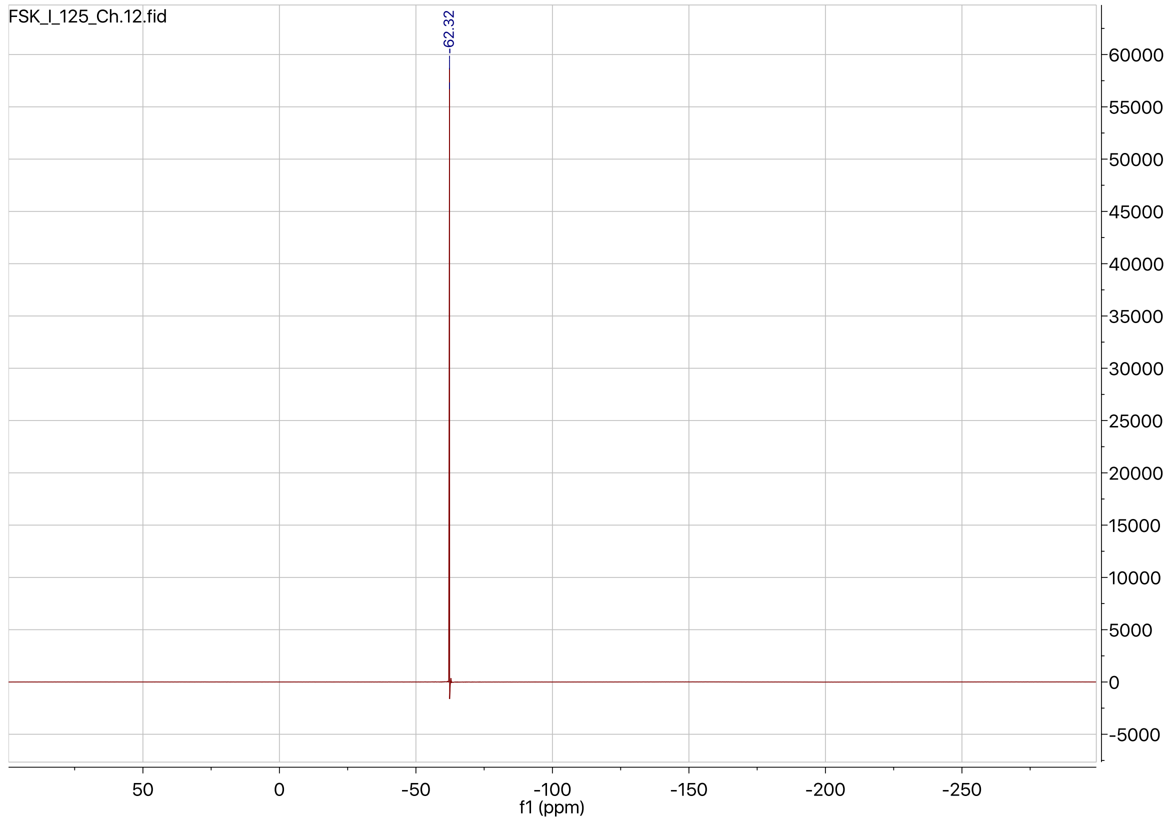
FSK_I_125_Ch.12.fid
-62.32
60000
55000
50000
45000
40000
35000
30000
25000
20000
15000
10000
5000
0
-5000
50
0
-50
-100
-150
-200
-250
f1 (ppm)

**Figure S5.** $^{1}$H and $^{13}$C NMR spectra of Ni(IPr)(2-(pyrid-2-yl)phenyl)Cl (**8**) in $CD_2Cl_2$.

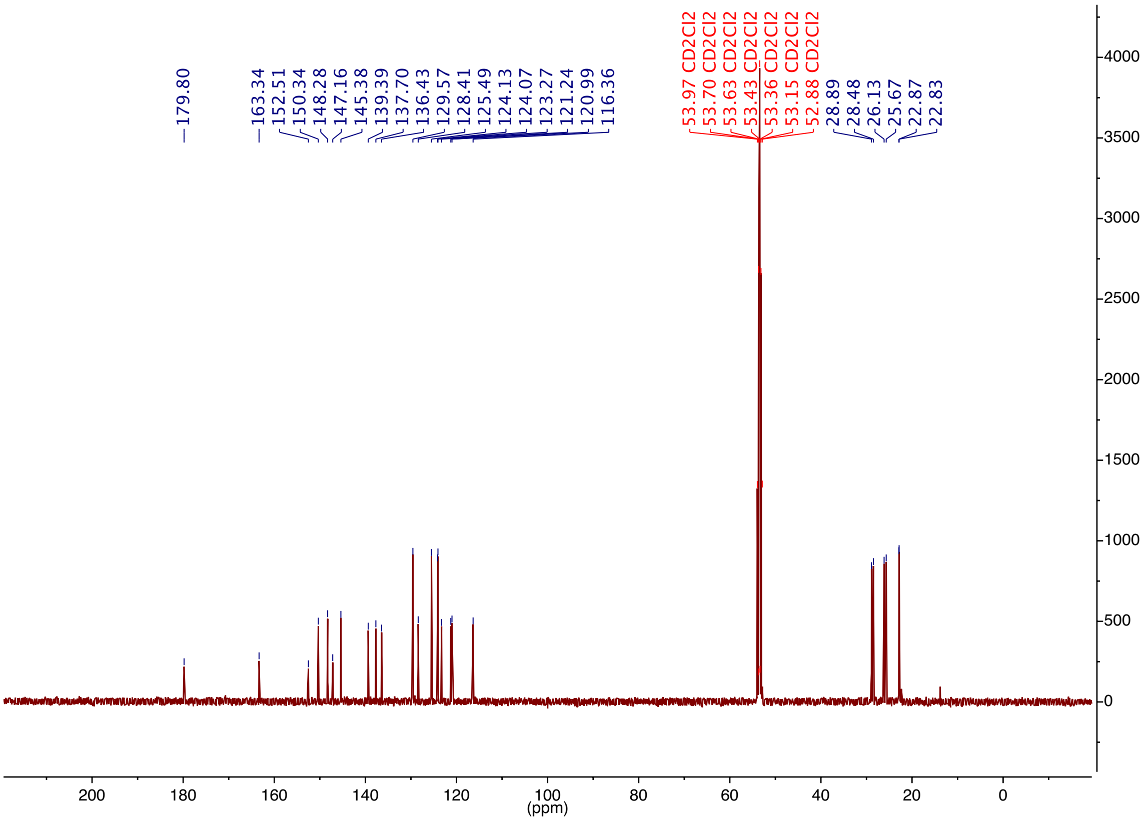


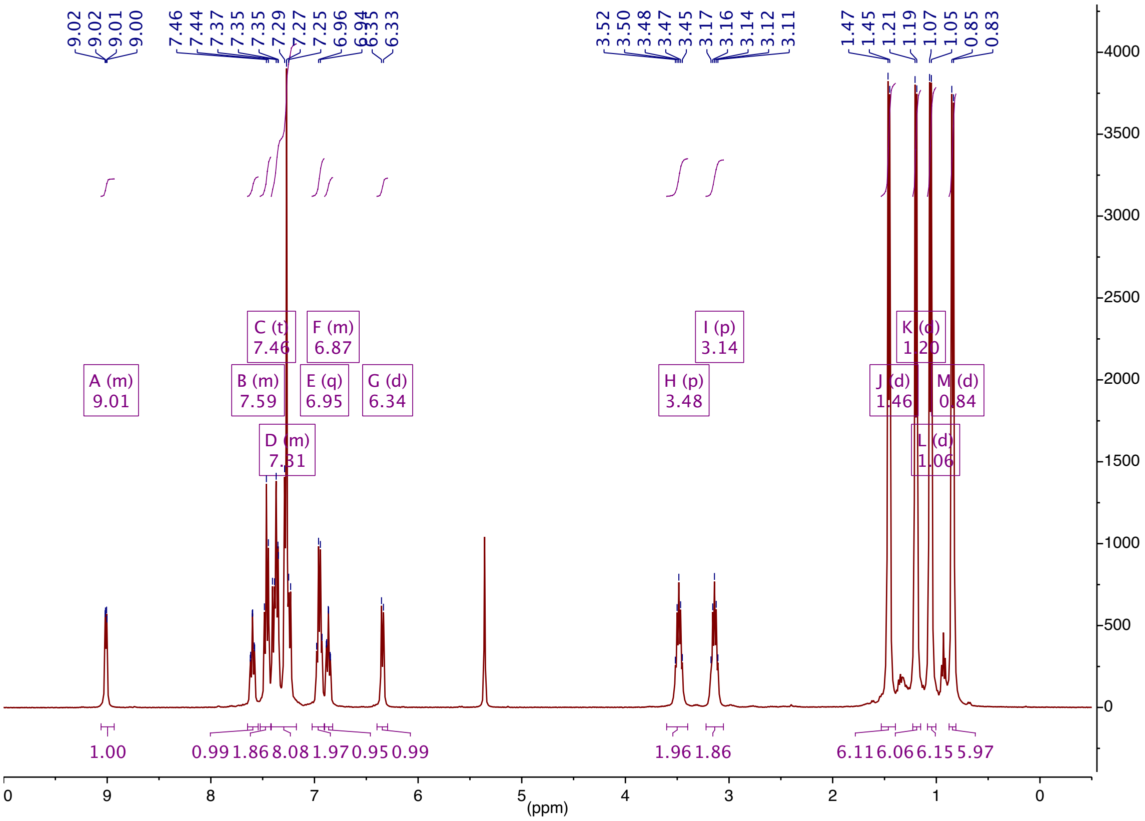

**Figure S6.** $^{1}$H, $^{13}$C, and $^{19}$F NMR spectra of Ni(IPr)(2-(5-fluoropyrid-2-yl)phenyl)Cl (**9**) in $CD_2Cl_2$.

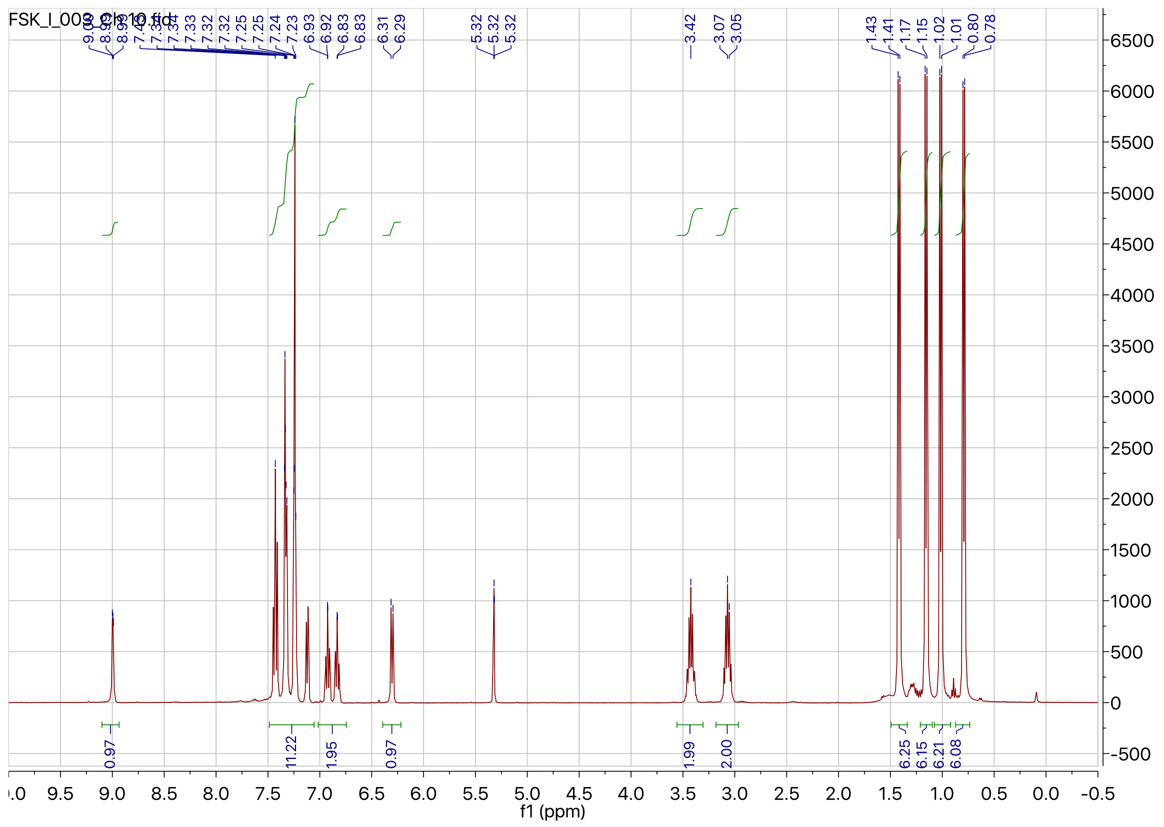


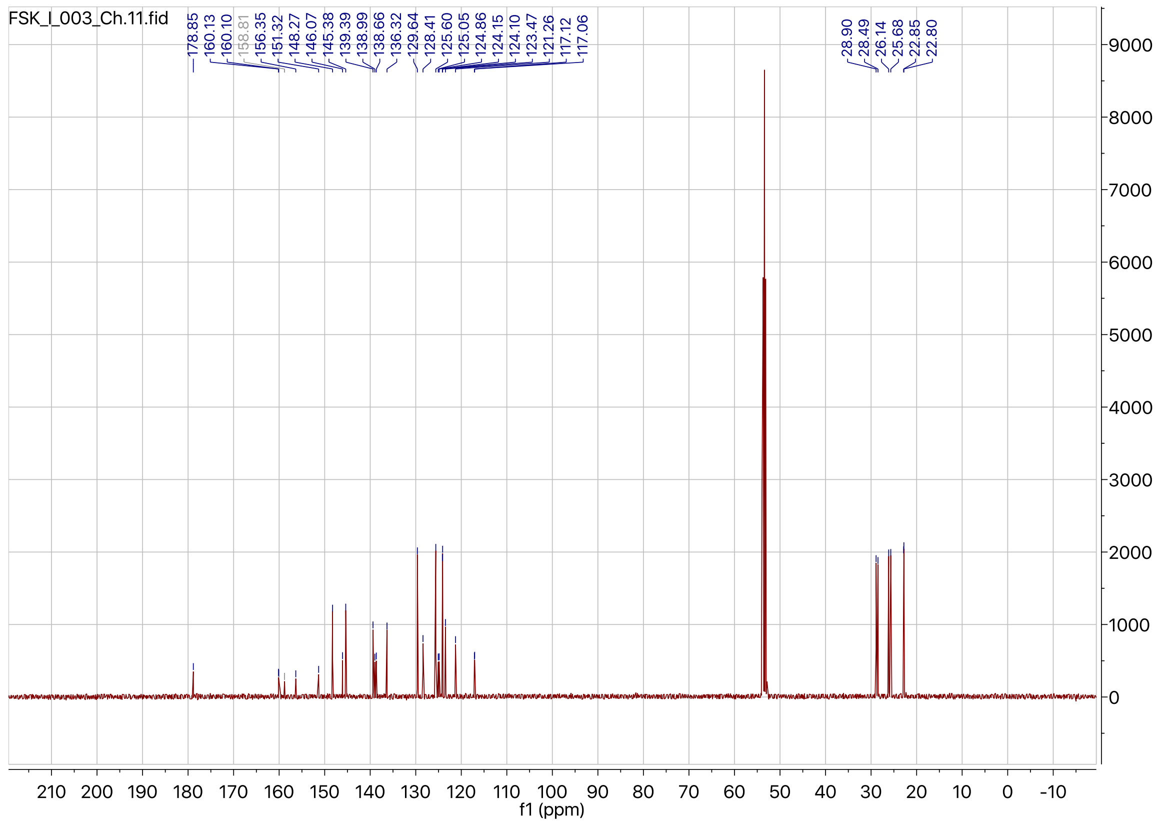

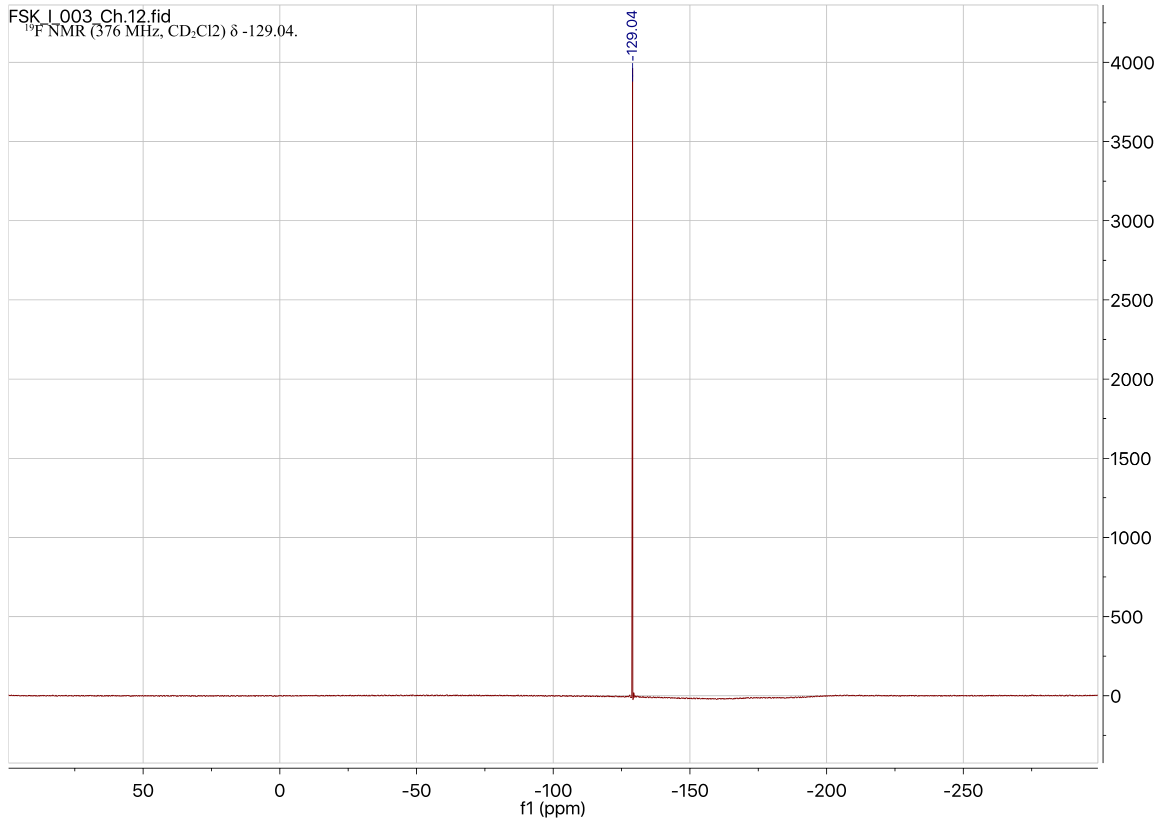


**Figure S7.** $^{1}$H, $^{13}$C, and $^{19}$F NMR spectra of Ni(IPr)(2-(5-trifluoromethylpyrid-2-yl)phenyl)Cl (**10**) in $CD_2Cl_2$.

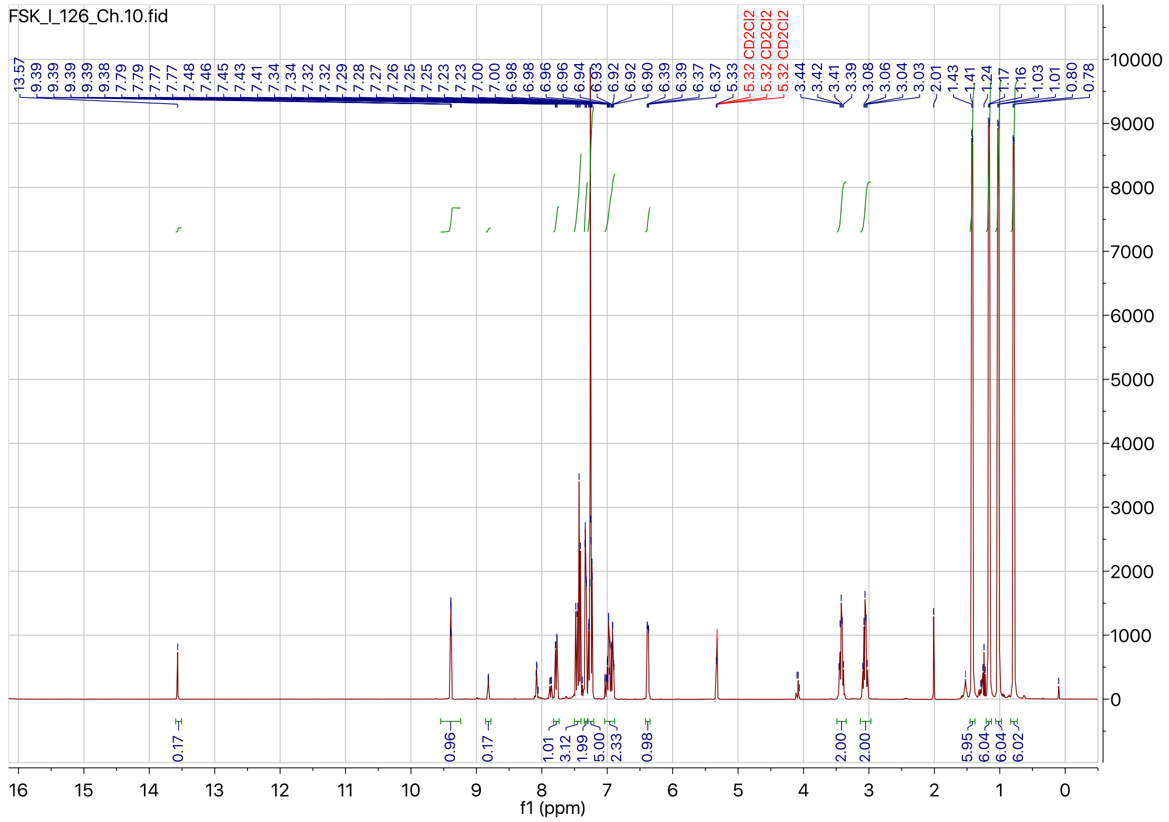

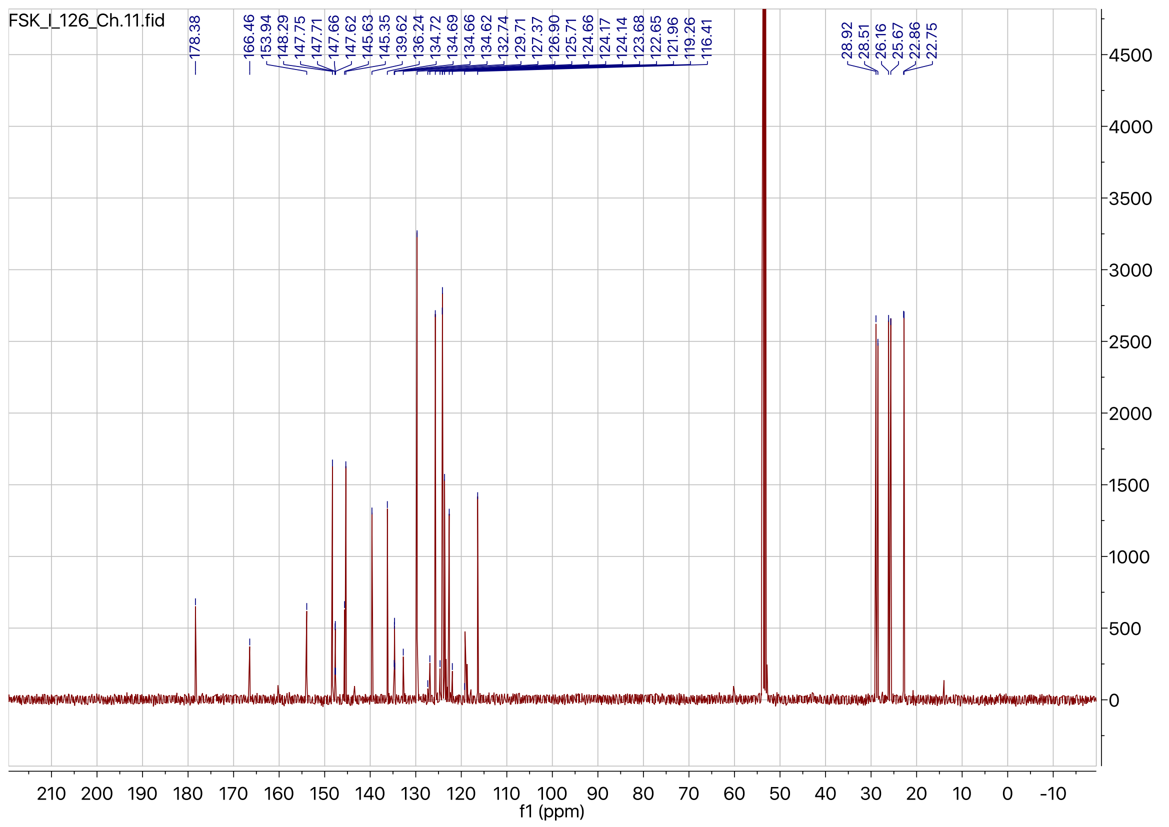
FSK_I_126_Ch.11.fid
178.38
166.46
153.94
148.29
147.75
147.71
147.66
147.62
145.63
145.35
139.62
136.24
134.72
134.69
134.66
134.62
132.74
129.71
127.37
126.90
125.71
124.66
124.17
124.14
123.68
122.65
121.96
119.26
116.41
28.92
28.51
26.16
25.67
22.86
22.75
f1 (ppm)

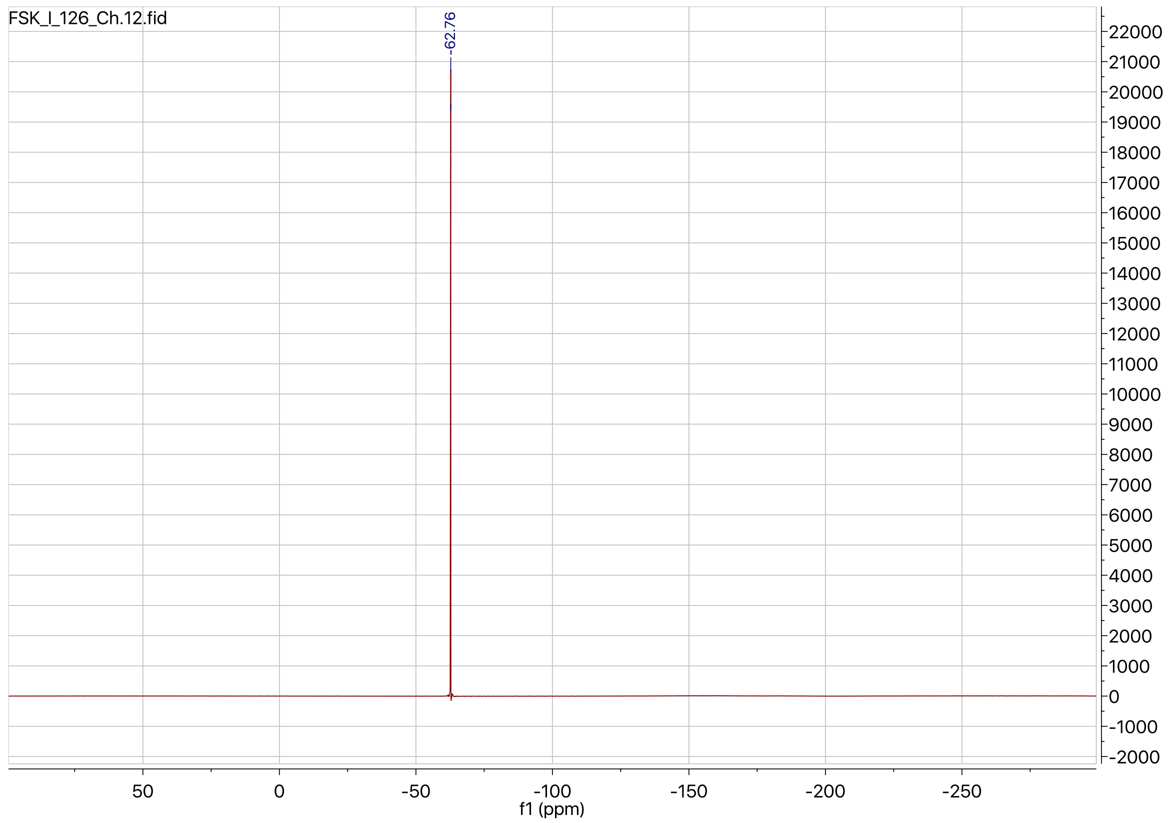
FSK_I_126_Ch.12.fid
-62.76
f1 (ppm)

**Figure S8.** $^1$H and $^{13}$C NMR spectra of 1-(2-chlorophenyl)-*N*,1-diphenylmethanimine in $CDCl_3$.

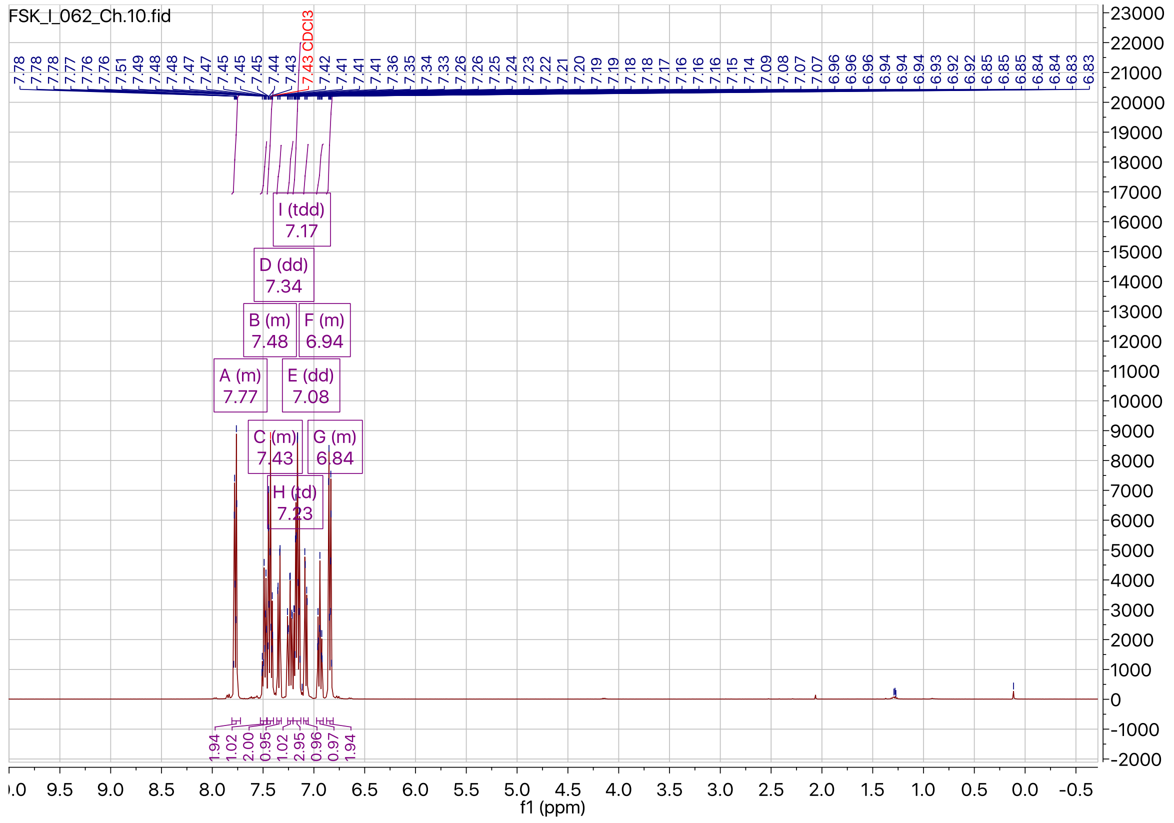


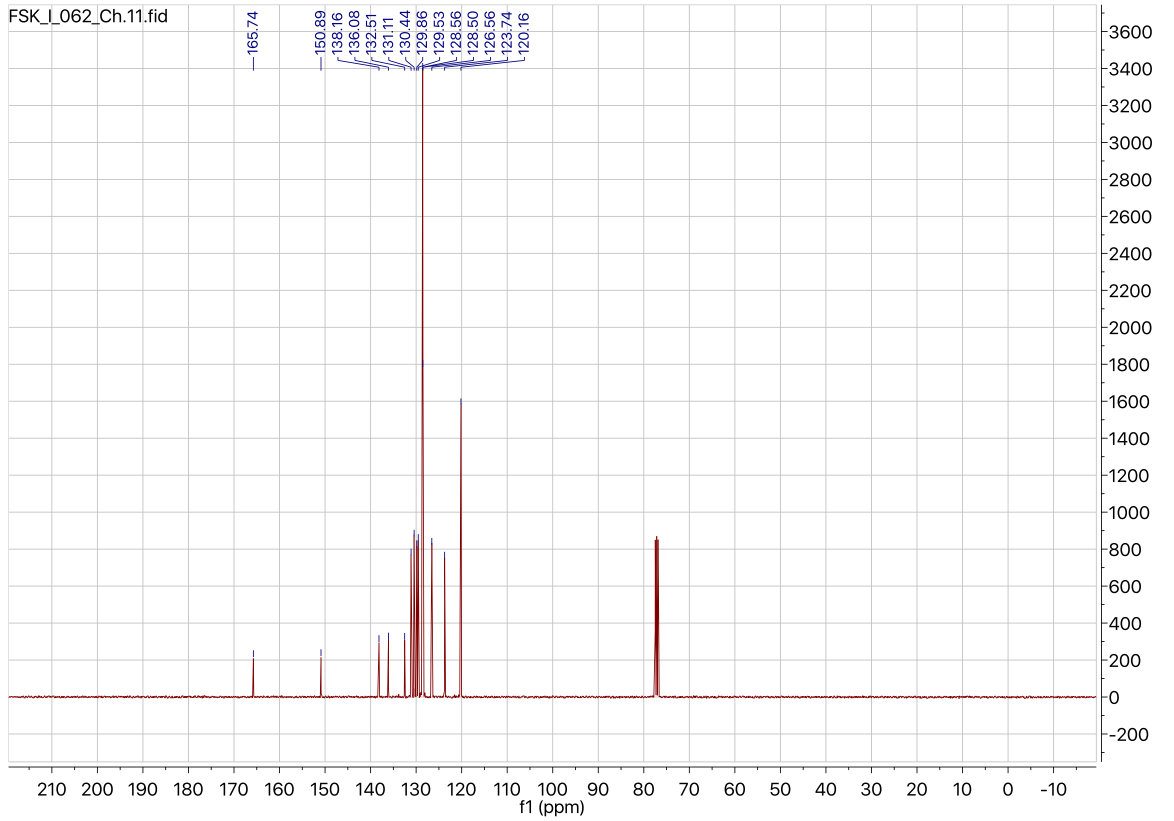

**Figure S9.** $^{1}H$ and $^{13}C$ NMR spectra of Ni(IPr[2-(*N*,1-diphenyliminomethyl)phenyl]Cl (**11**) in $CD_2Cl_2$.

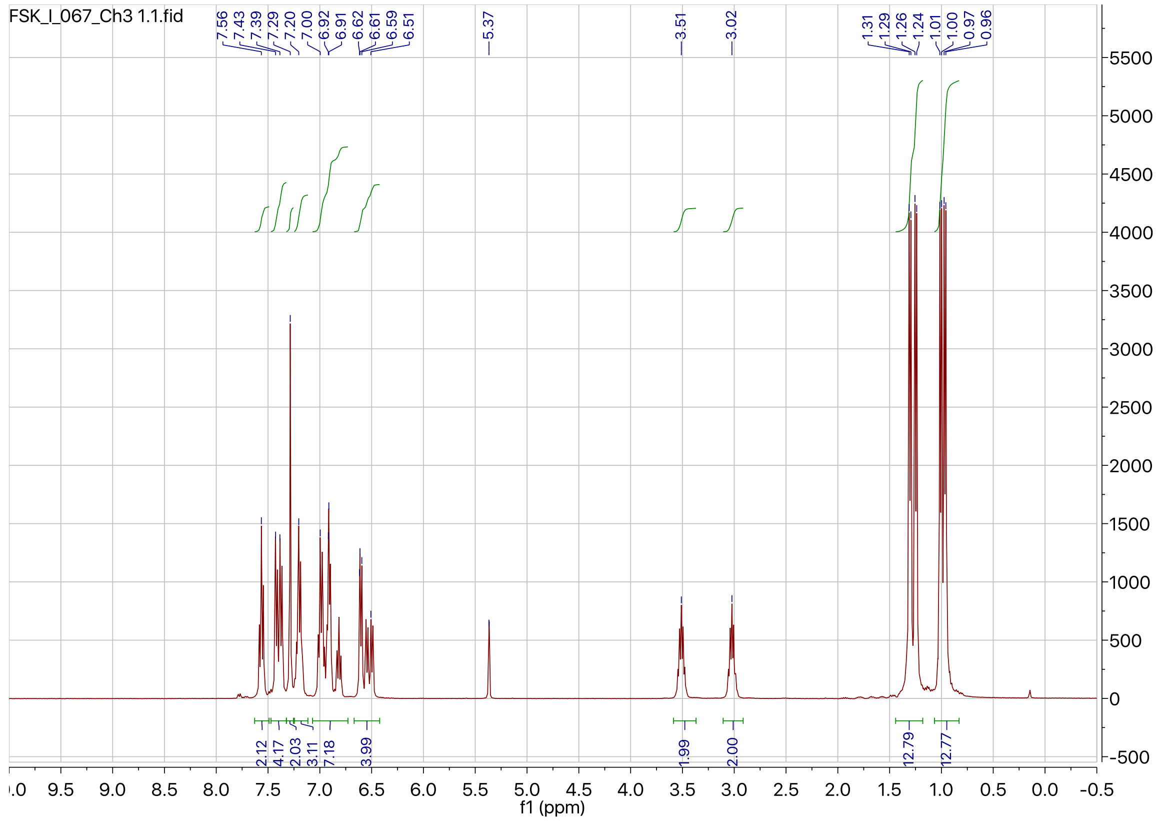


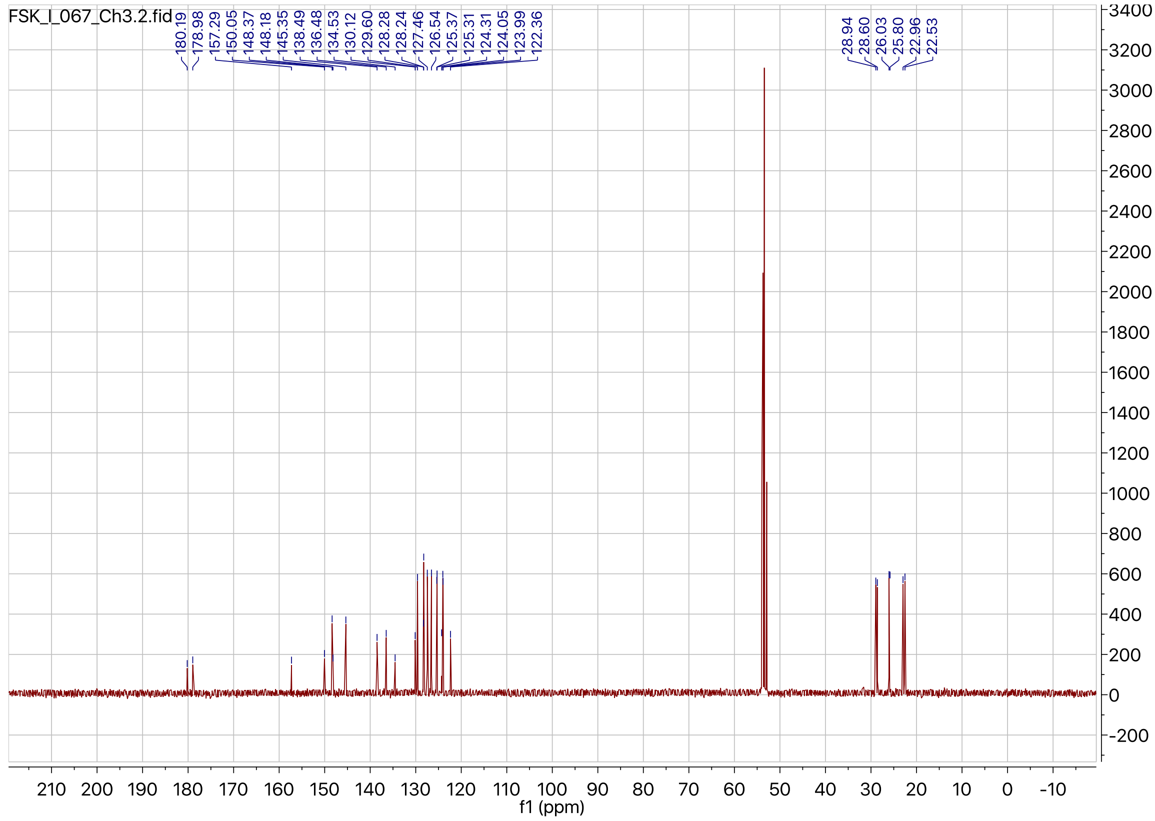

**Figure S10.** $^{1}H$, $^{13}C$, and $^{31}P$ NMR spectra of (2,2'-biphenyl)(2-chlorophenyl)phosphite in $CDCl_3$.

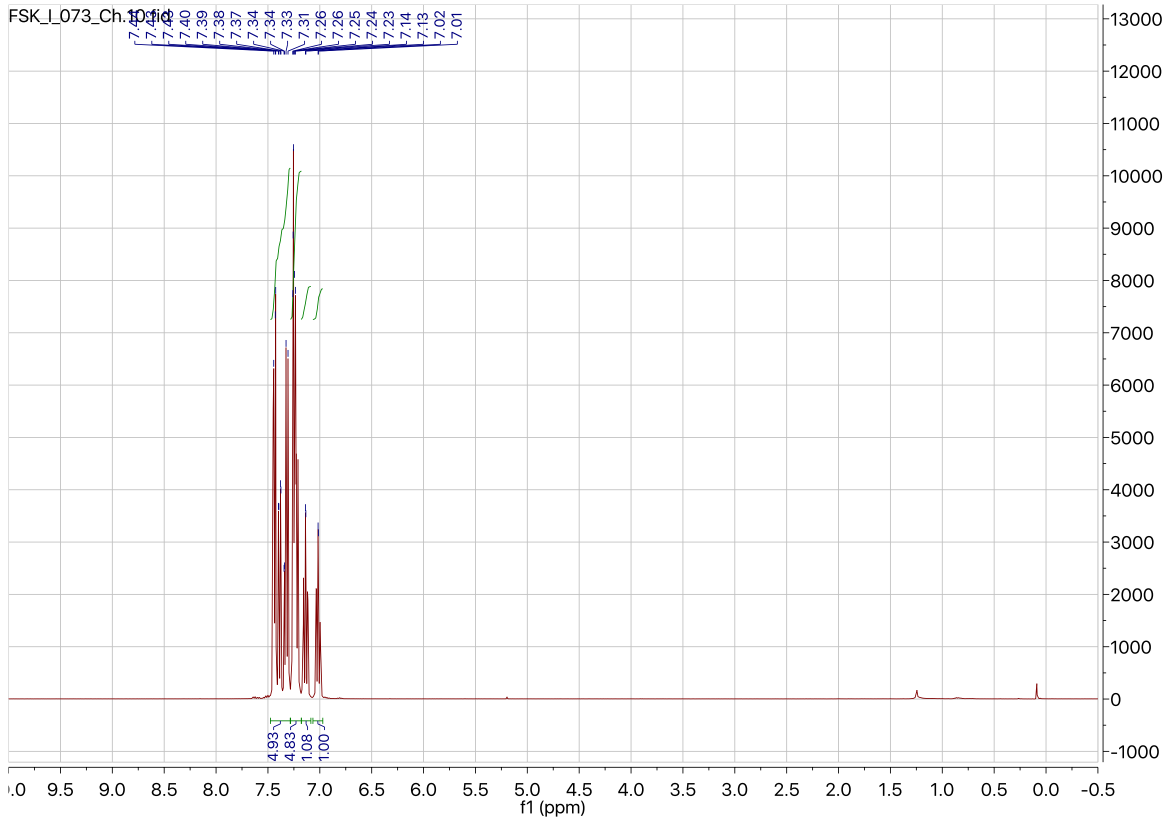


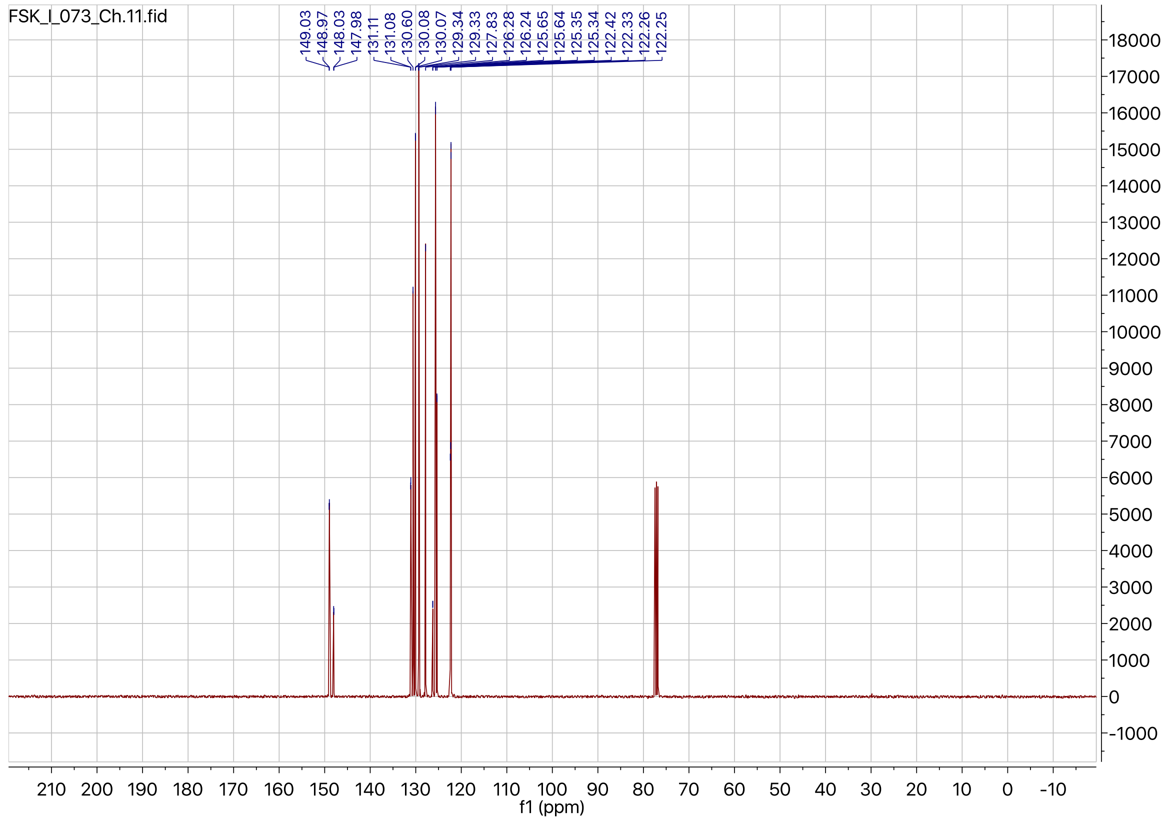

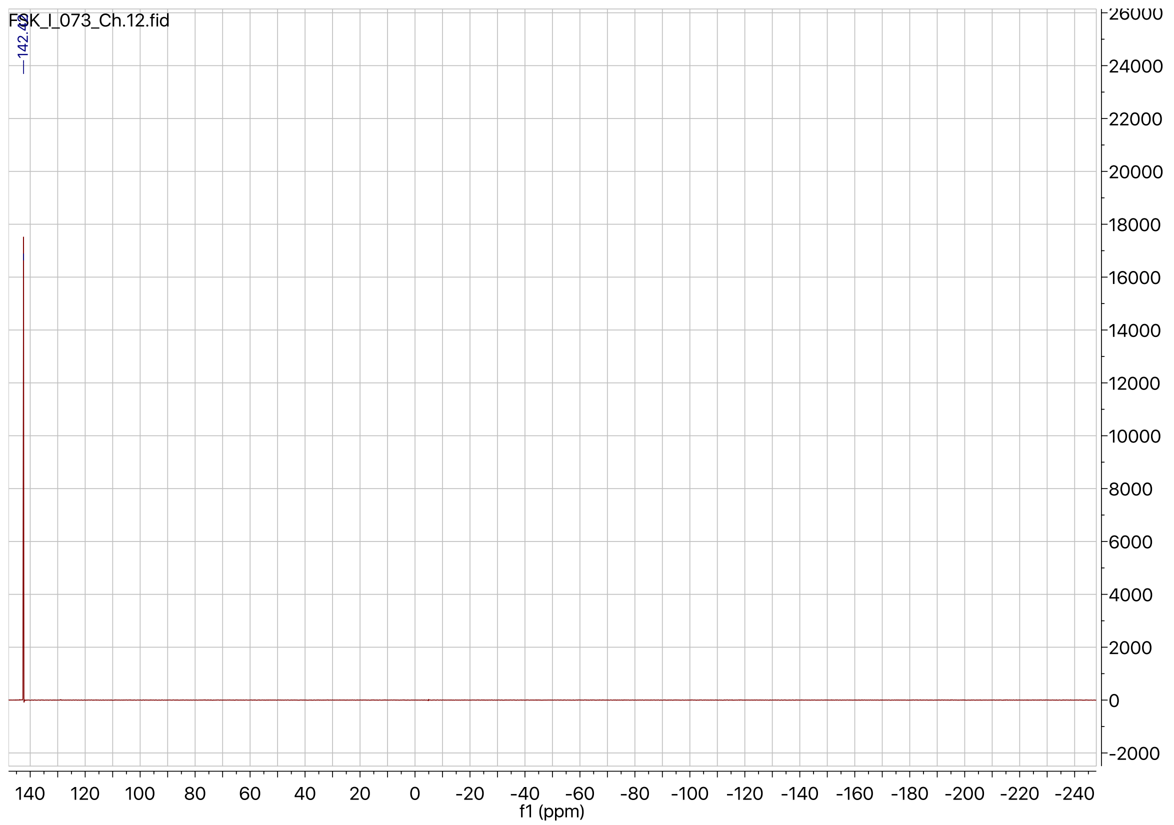

**Figure S11.** $^1H$, $^{13}C$, and $^{31}P$ NMR spectra of Ni(IPr)($\eta^2$-(2((2,2'-biphenyl)phosphito)phenyl)Cl (**12**) in $CD_2Cl_2$.

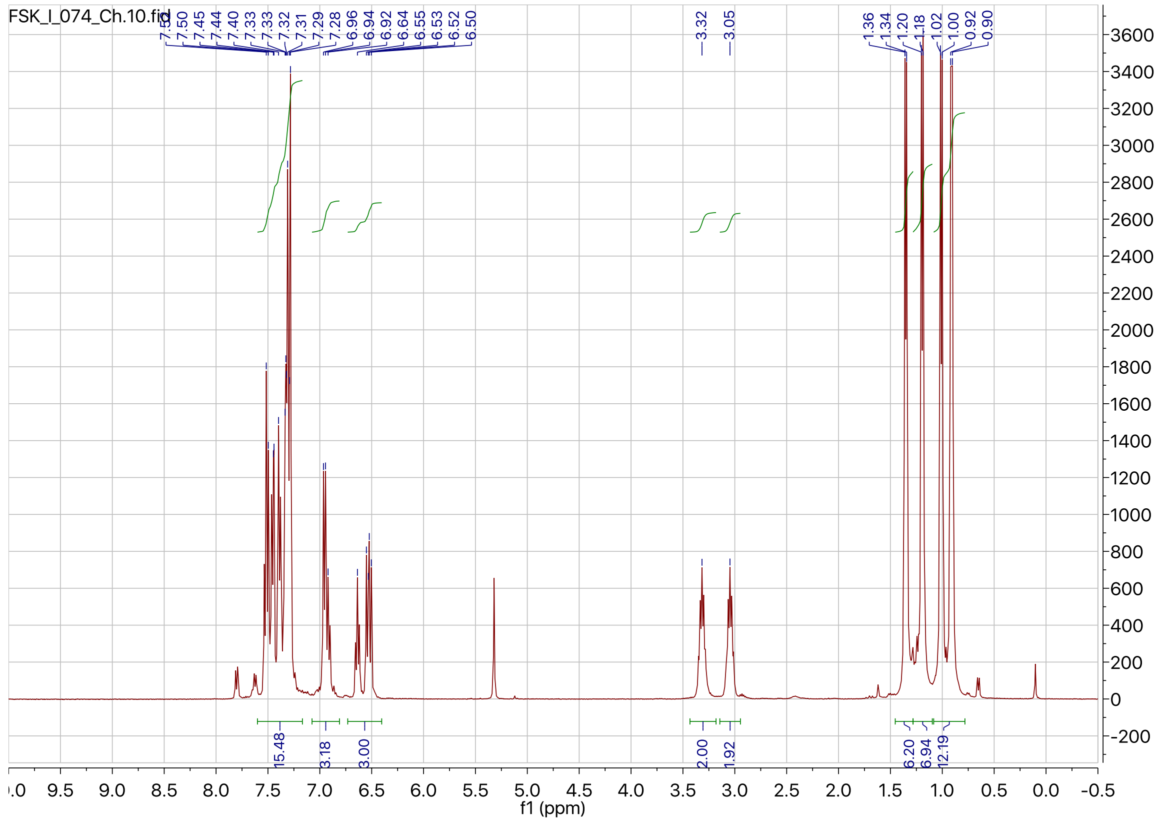

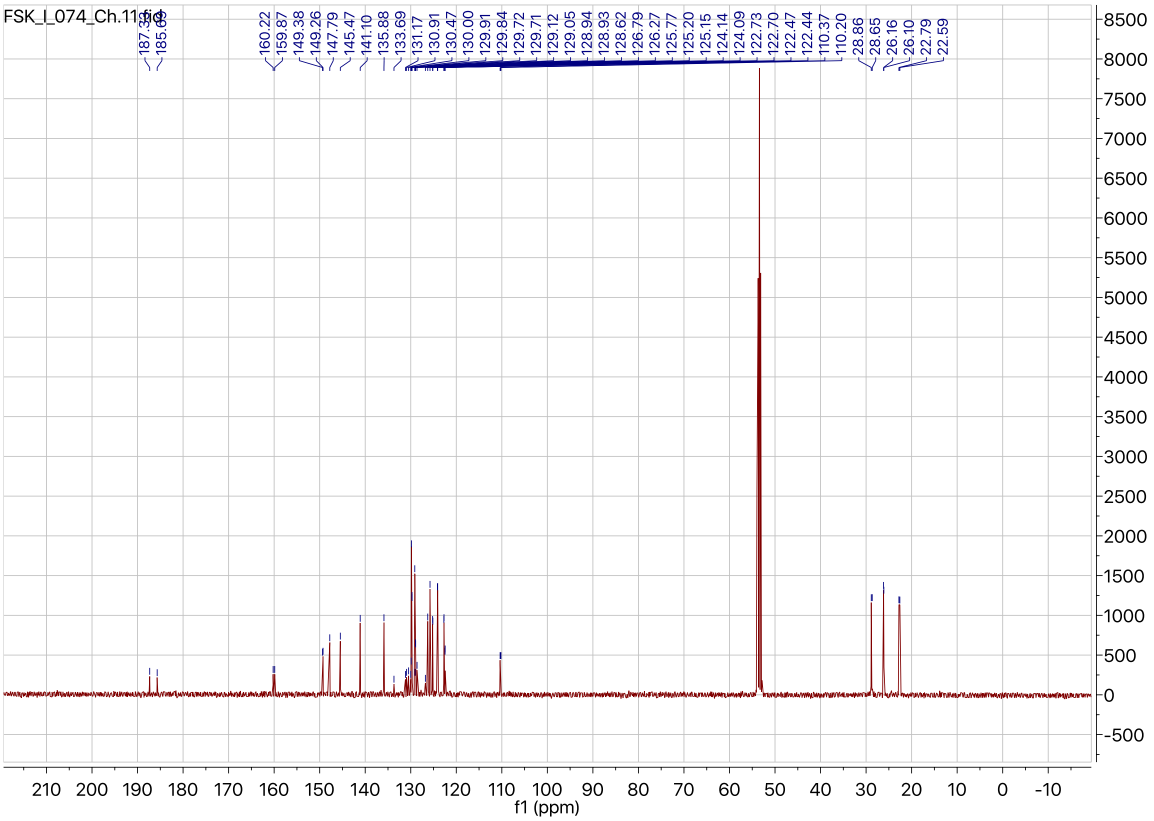
FSK_I_074_Ch.11.fid
160.22
159.87
149.38
149.26
147.79
145.47
141.10
135.88
133.69
131.17
130.91
130.47
130.00
129.91
129.84
129.72
129.71
129.12
129.05
128.94
128.93
128.62
126.79
126.27
125.77
125.20
125.15
124.14
124.09
122.73
122.70
122.47
122.44
110.37
110.20
28.86
28.65
26.16
26.10
22.79
22.59
f1 (ppm)

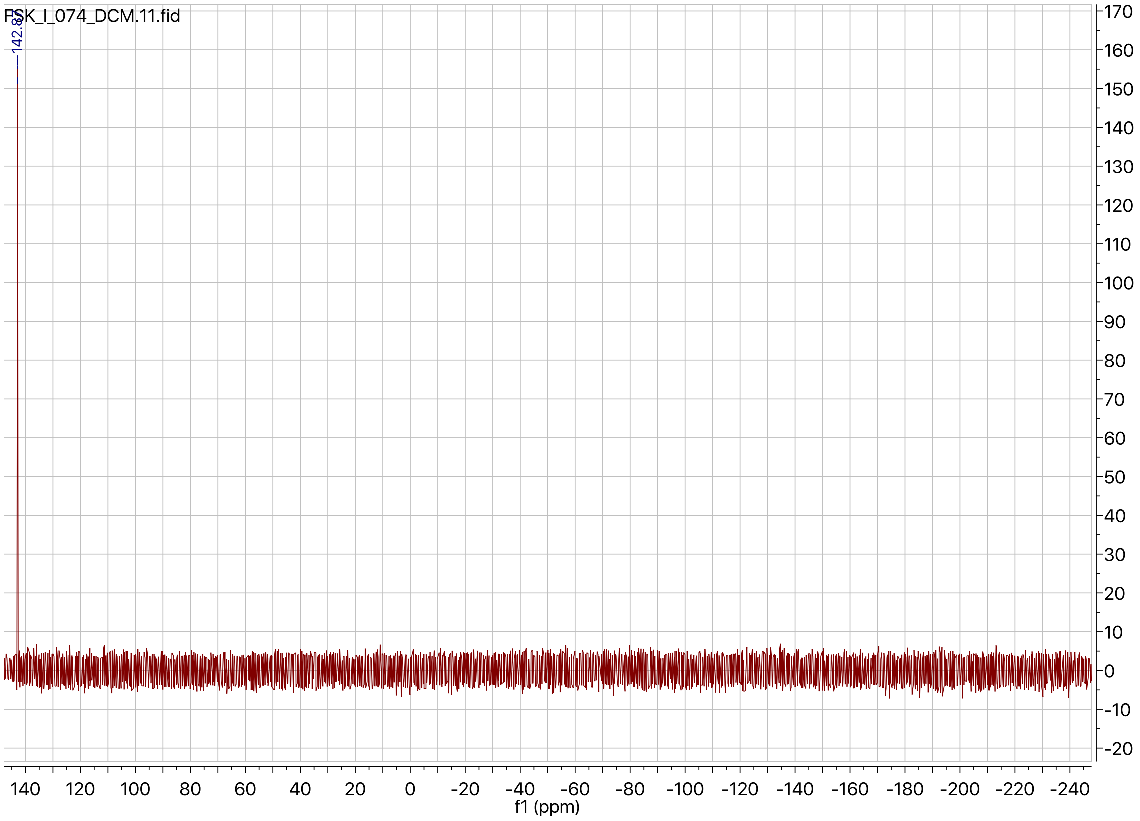
FSK_I_074_DCM.11.fid
f1 (ppm)

**Figure S12.** $^{1}H$ and $^{13}C$ NMR spectra of Ni(IPr)[2-(*N*-morpholylmethyl)phenyl]Cl (**13**) in $CD_2Cl_2$.

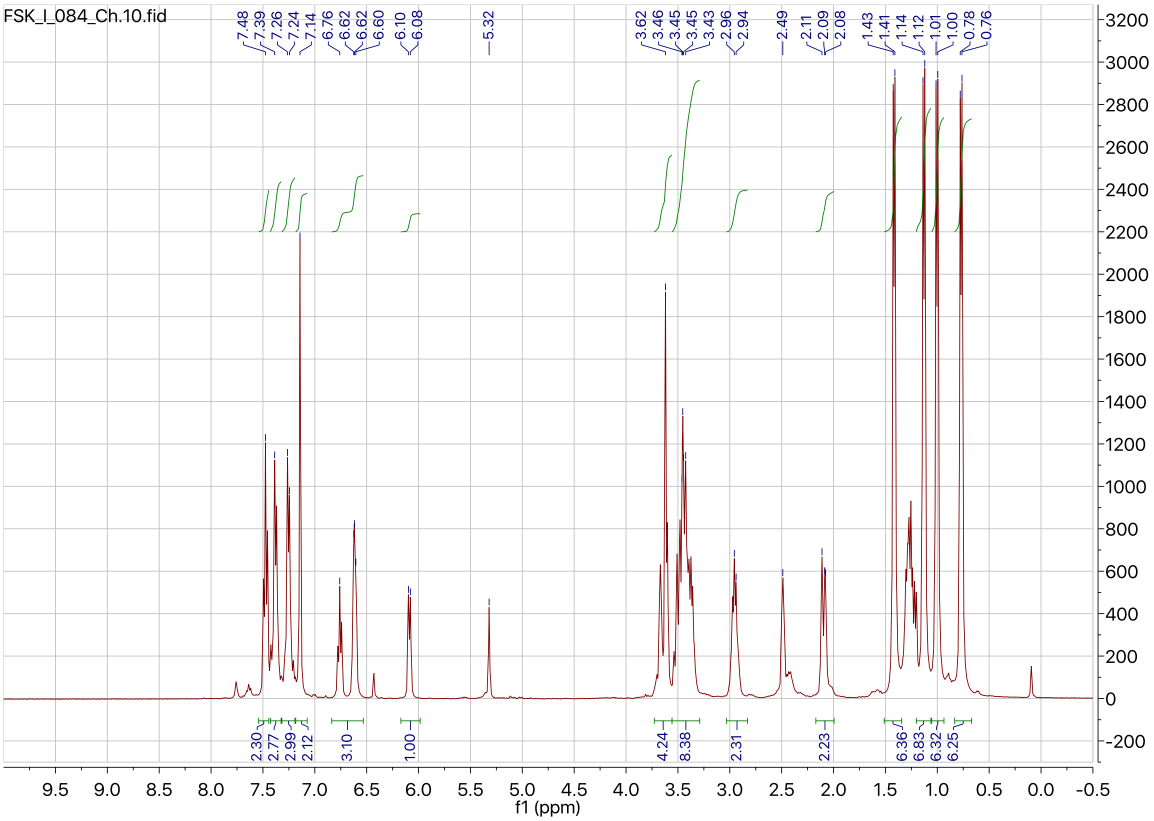


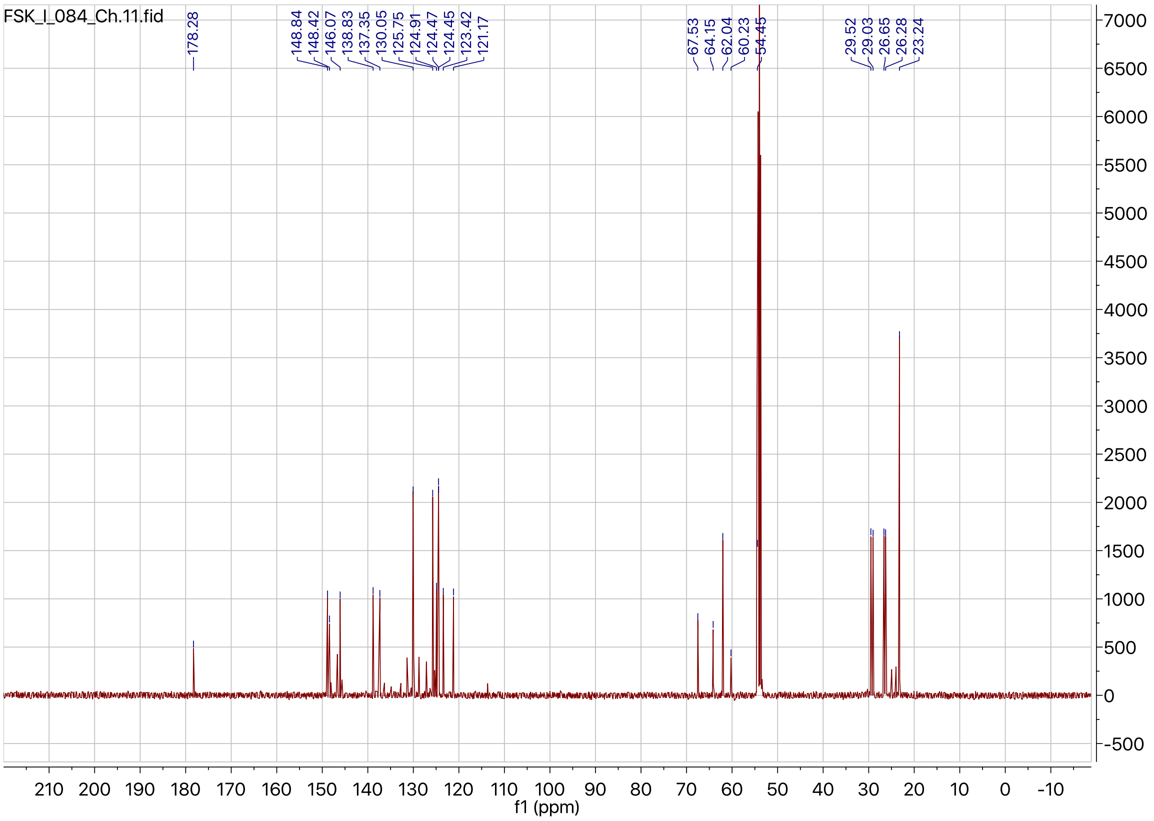

**Figure S13.** $^{1}H$ and $^{13}C$ NMR spectra of Ni(IPr)[2-(*N*-pyrrolidylmethyl)phenyl]Cl (**14**) in $CD_2Cl_2$.

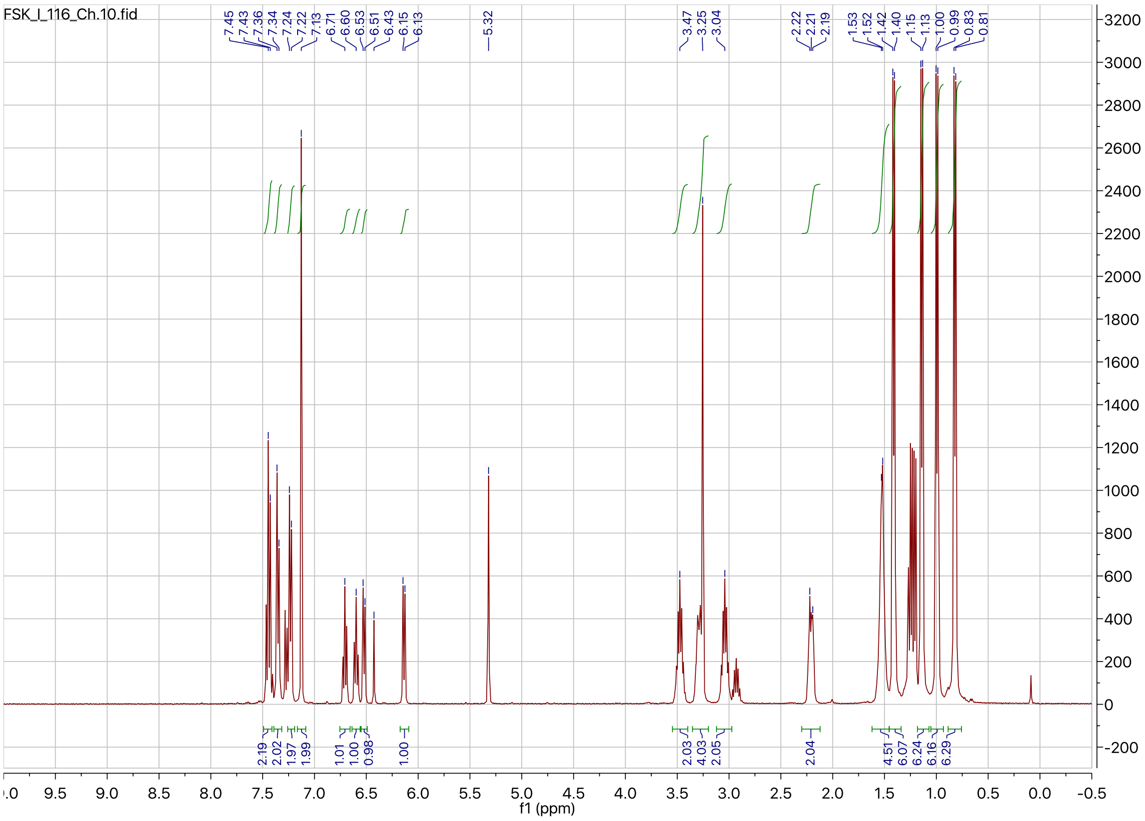


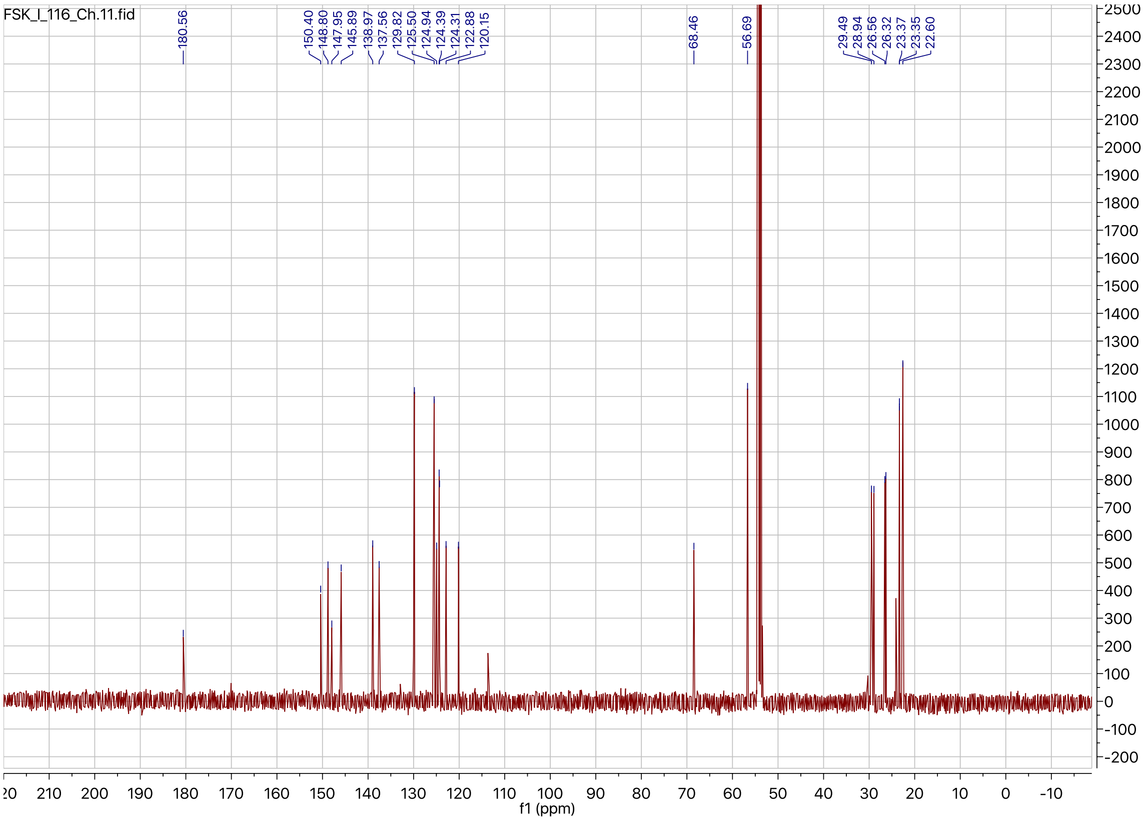

**Figure S14.** $^{1}$H and $^{13}$C NMR spectra of Ni(IPr)[2-(*N*-piperidylmethyl)phenyl]Cl (**15**) in $CD_2Cl_2$.

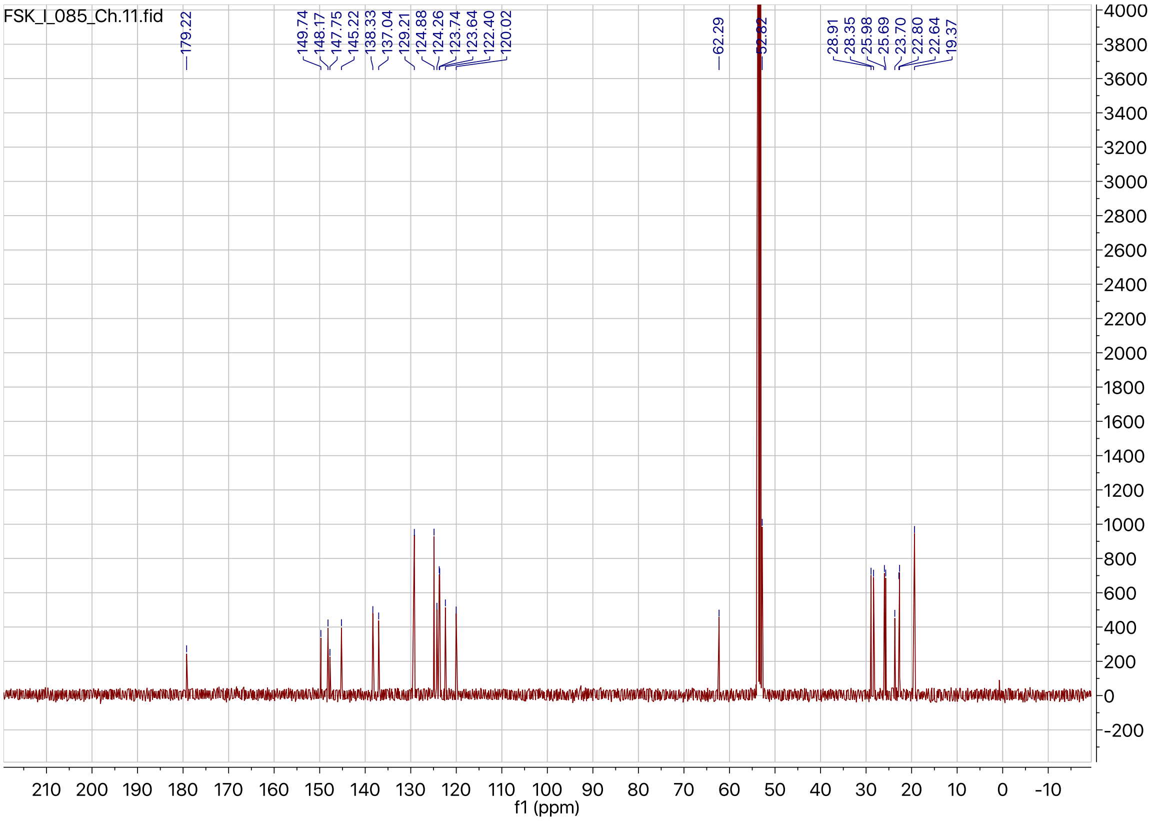

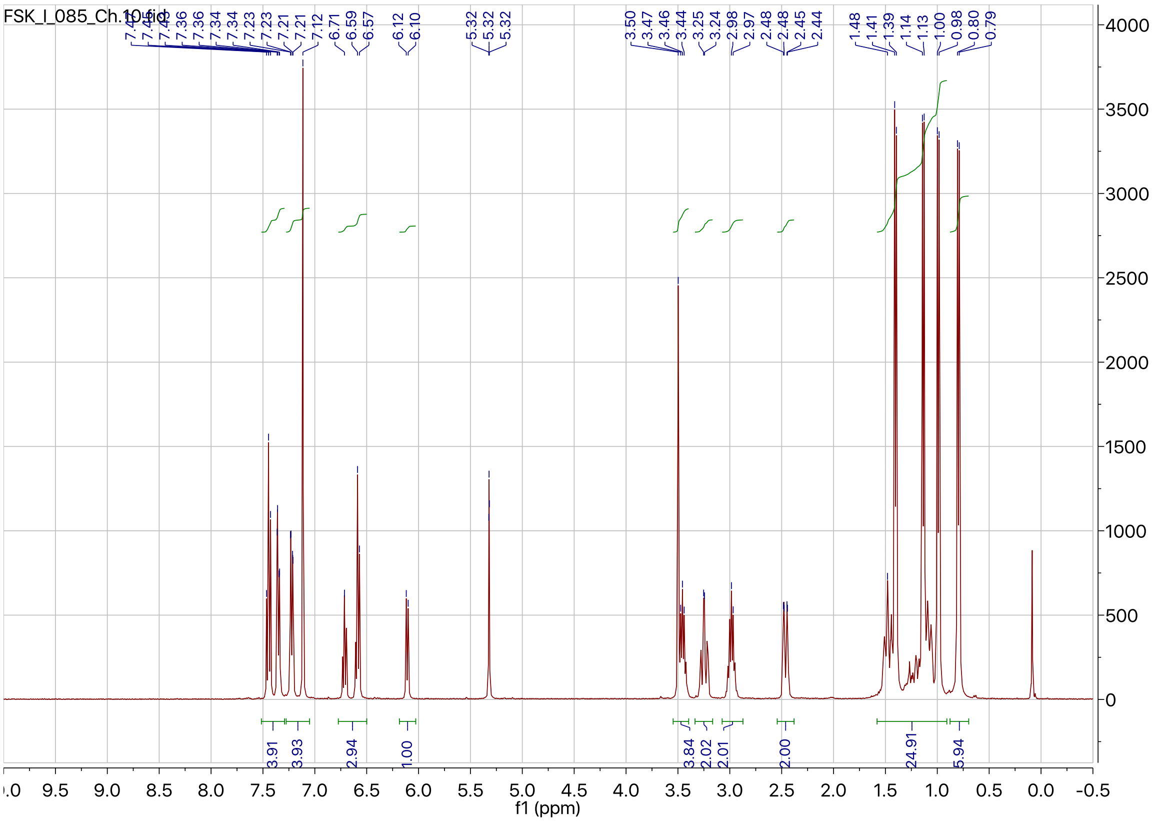

**Figure S15.** $^{1}H$ and $^{13}C$ NMR spectra of *N*-(1-(2-chlorophenyl)-but-3-en-1-yl)piperadine in $CDCl_3$.

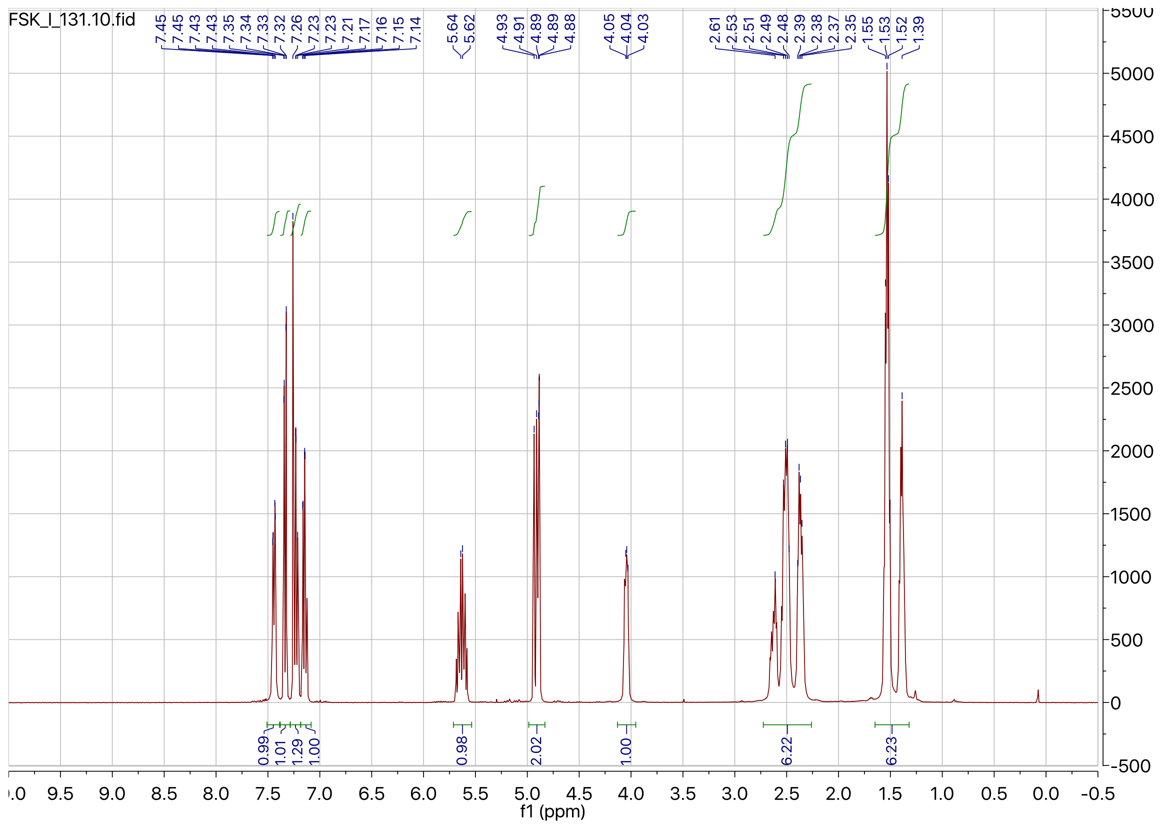


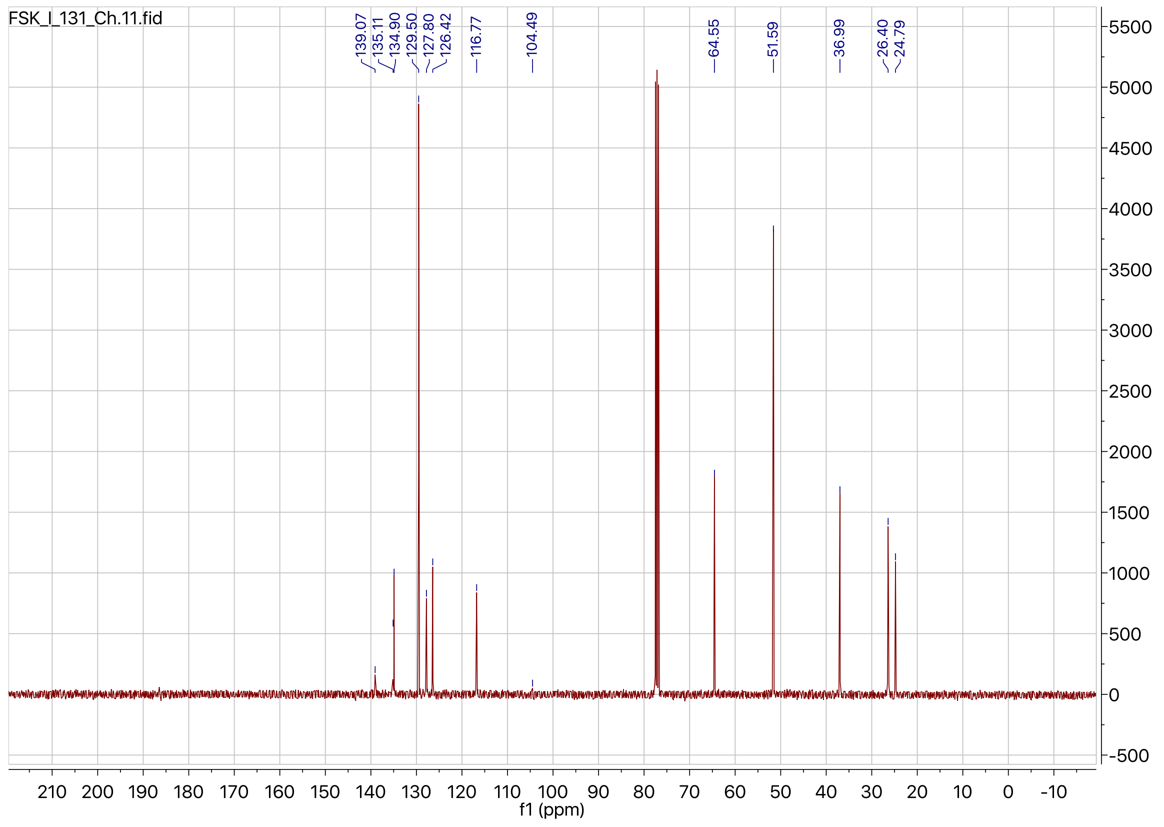

**Figure S16.** $^{1}H$ and $^{13}C$ NMR spectra of *N*-(1-(2-chlorophenyl)-but-3-en-1-yl)pyrrolidine in $CDCl_3$.

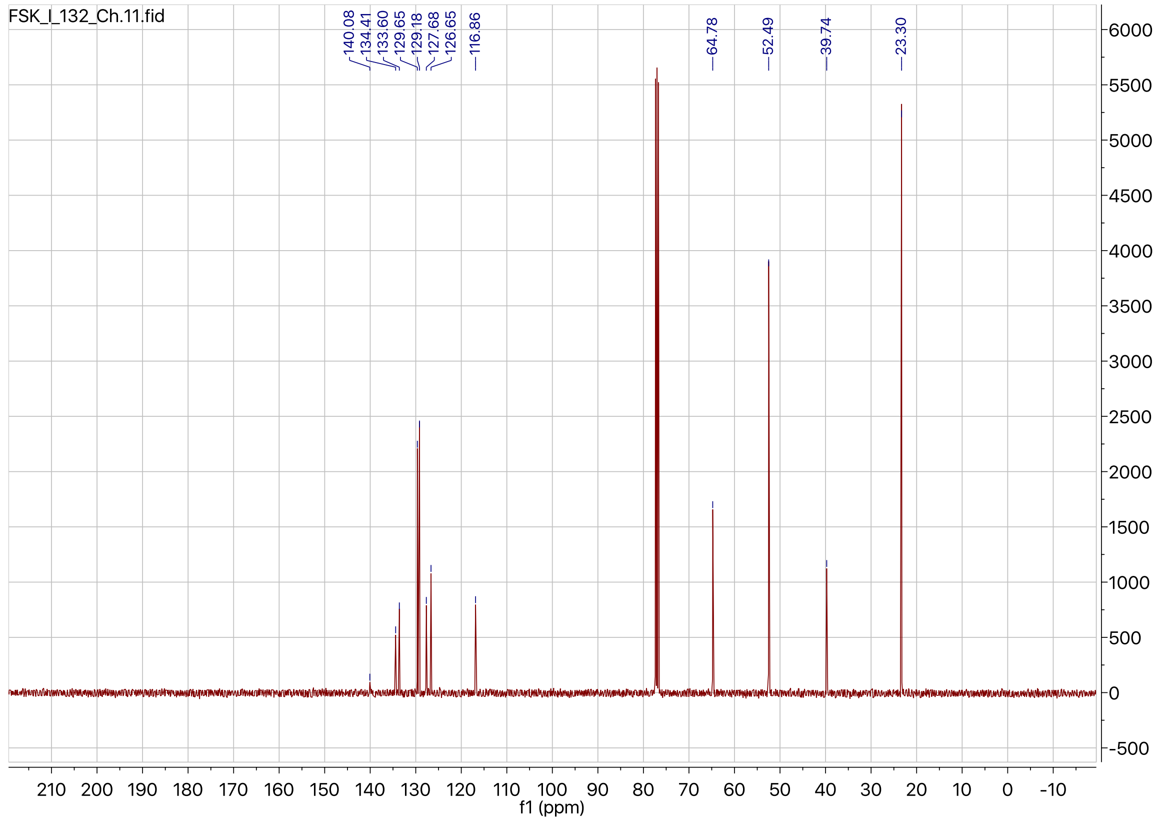


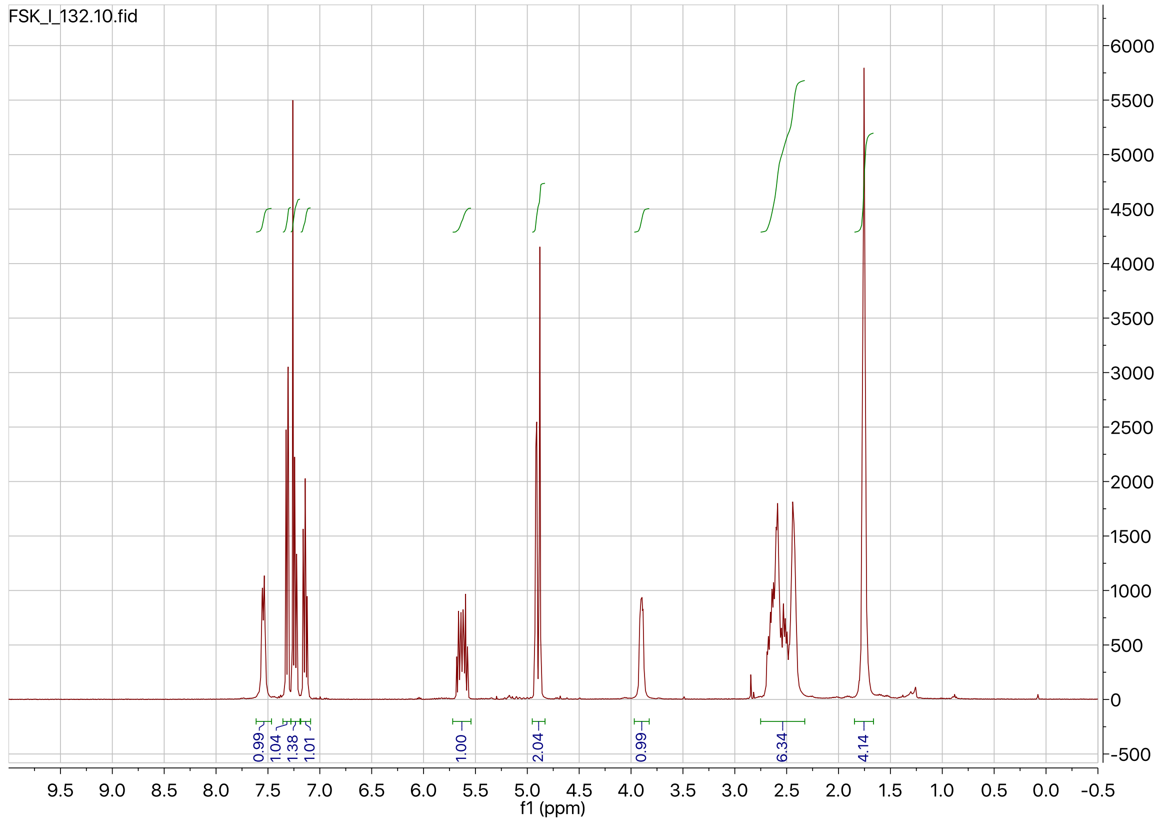

**Figure S17.** $^{1}H$ and $^{13}C$ NMR spectra of *N*-(1-(2-chlorophenyl)-hex-5-en-1-yl)piperadine in $CDCl_3$.

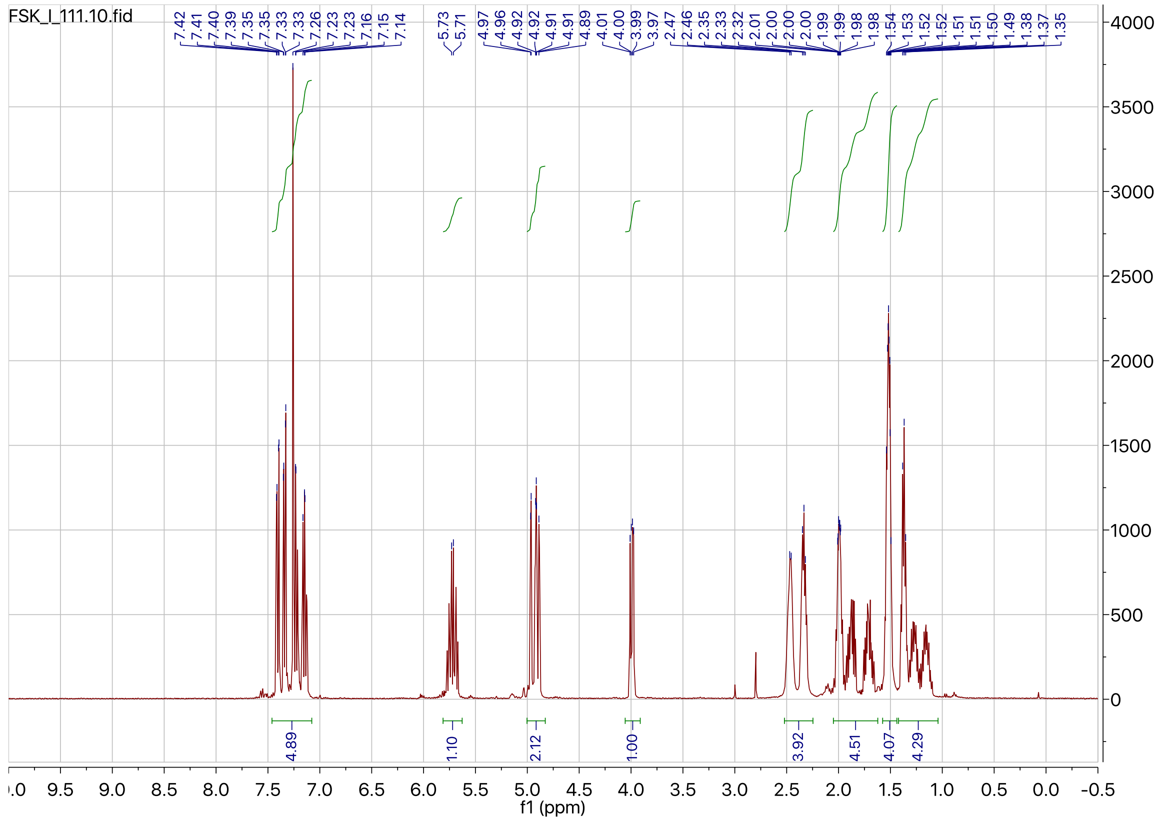


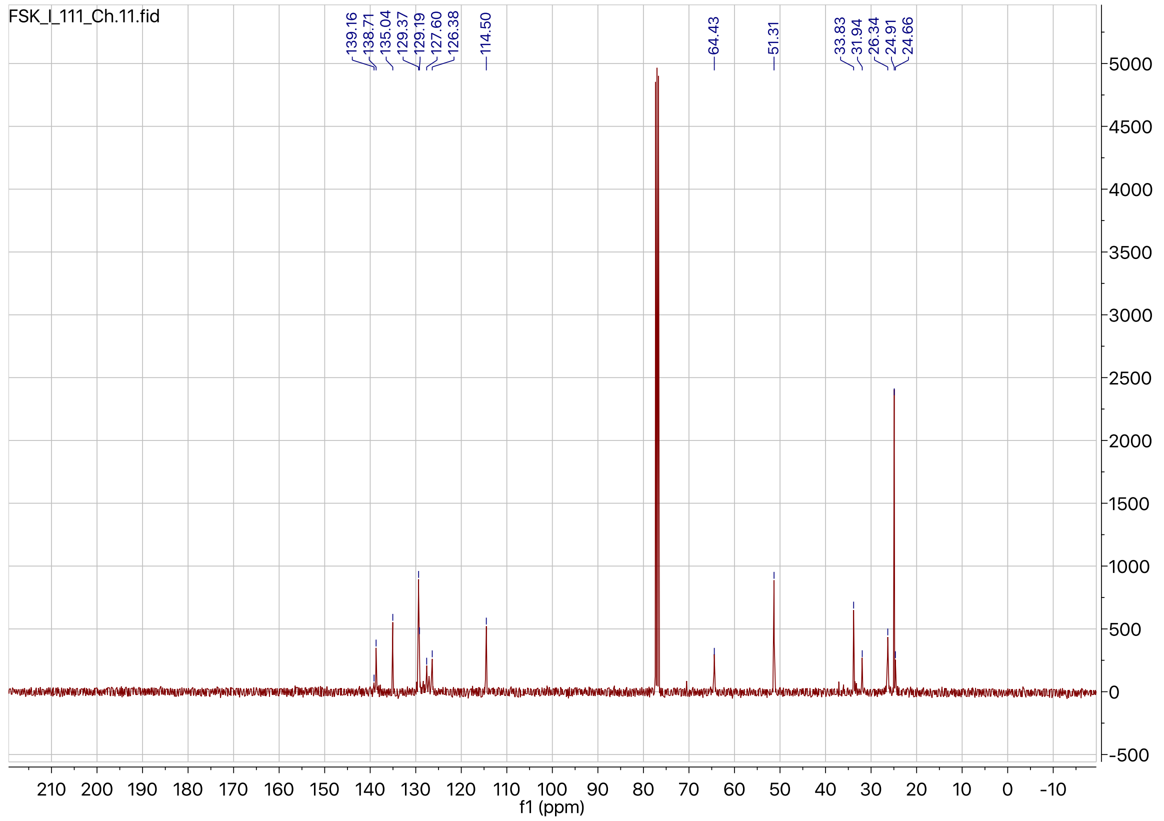

**Figure S18.** $^{1}H$ and $^{13}C$ NMR spectra of *N*-(1-(2-chlorophenyl)-but-1-yl)piperadine in $CDCl_3$.

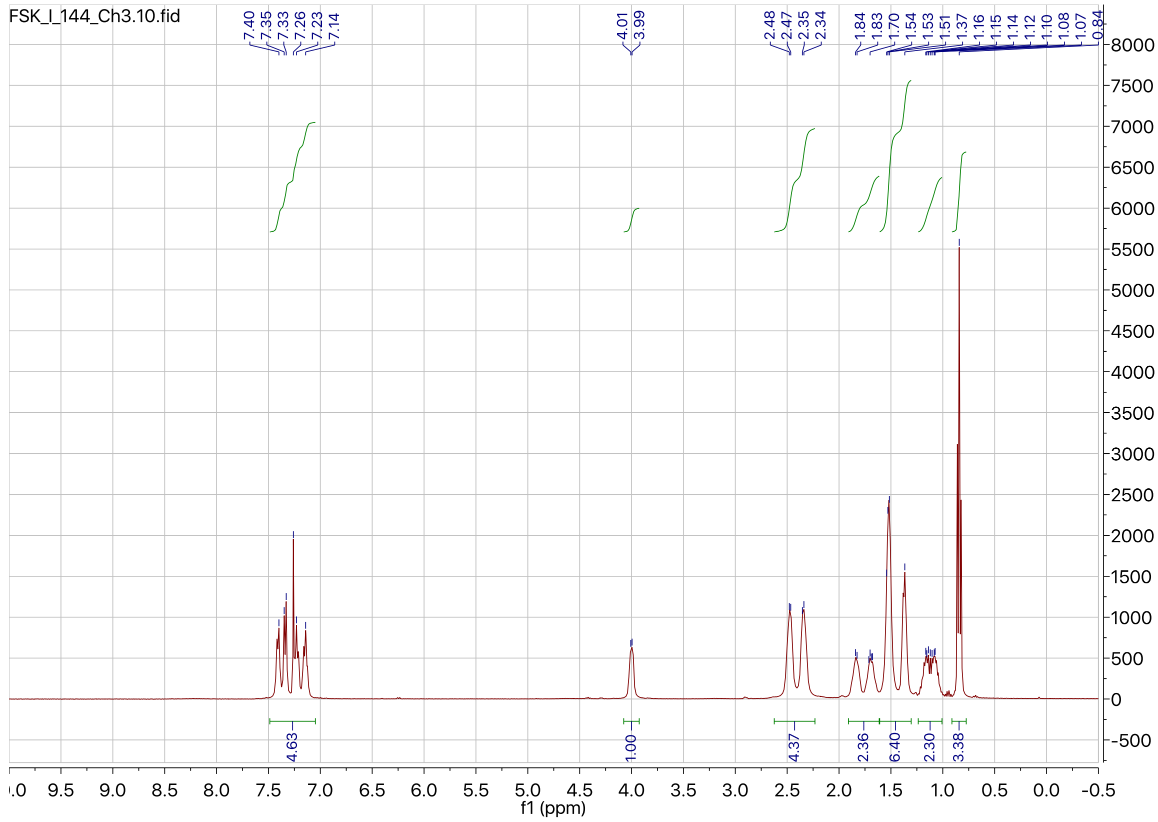


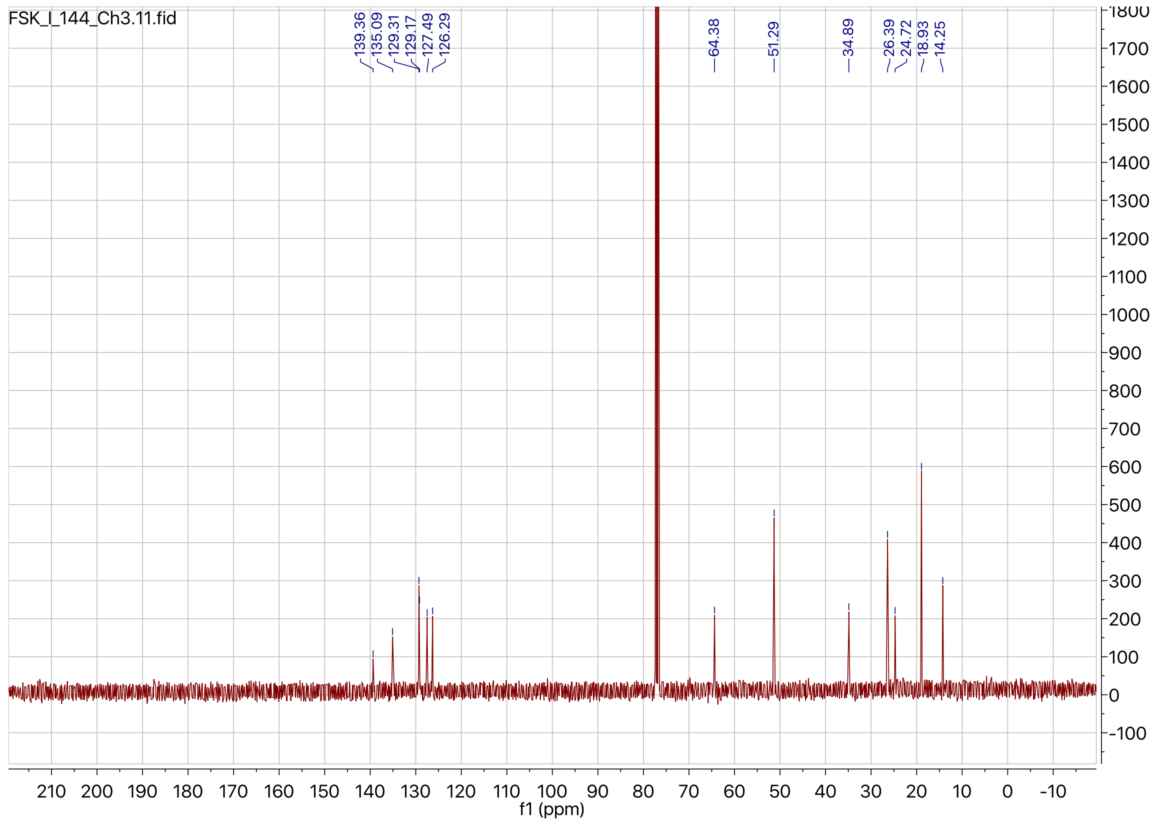

**Figure S19.** $^{1}H$ and $^{13}C$ NMR spectra of (IPr)[2-(1-(*N*-piperidyl)but-3-en-1-yl)phenyl]nickel(II)chloride (**5a**) in $CD_2Cl_2$.

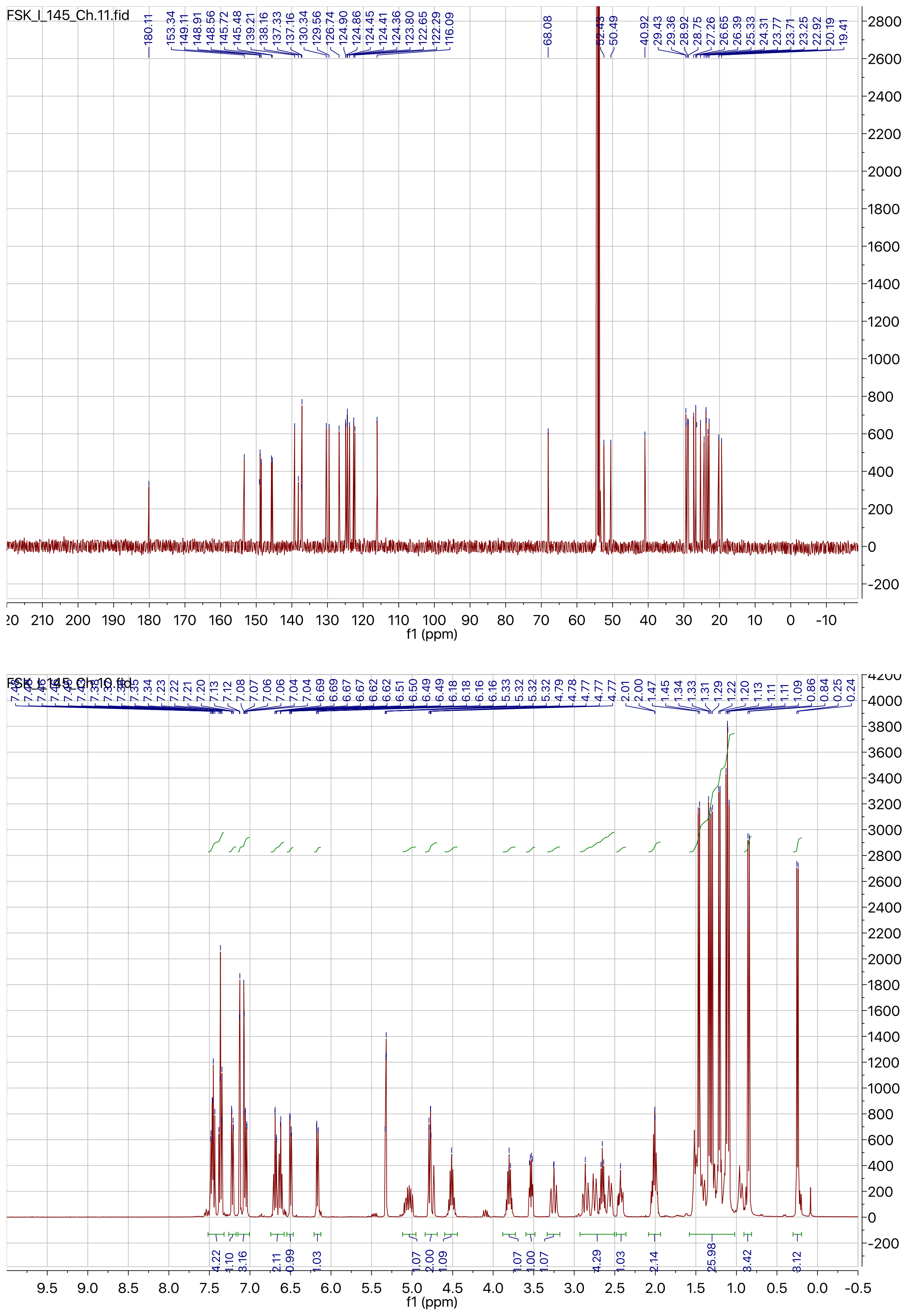

**Figure S20.** $^{1}H$ and $^{13}C$ NMR spectra of (IPr)[2-(1-(*N*-piperidyl)hex-5-en-1-yl)phenyl]nickel(II)chloride (**16**) in $CD_2Cl_2$.

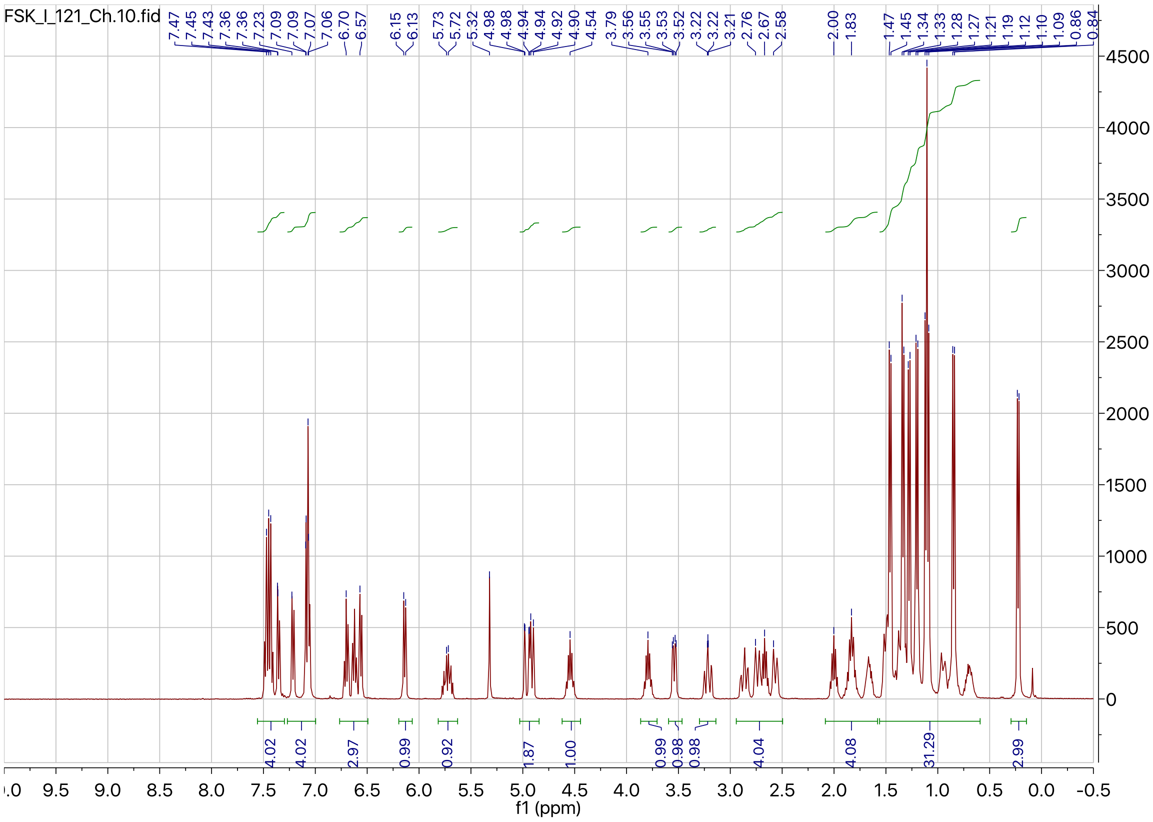

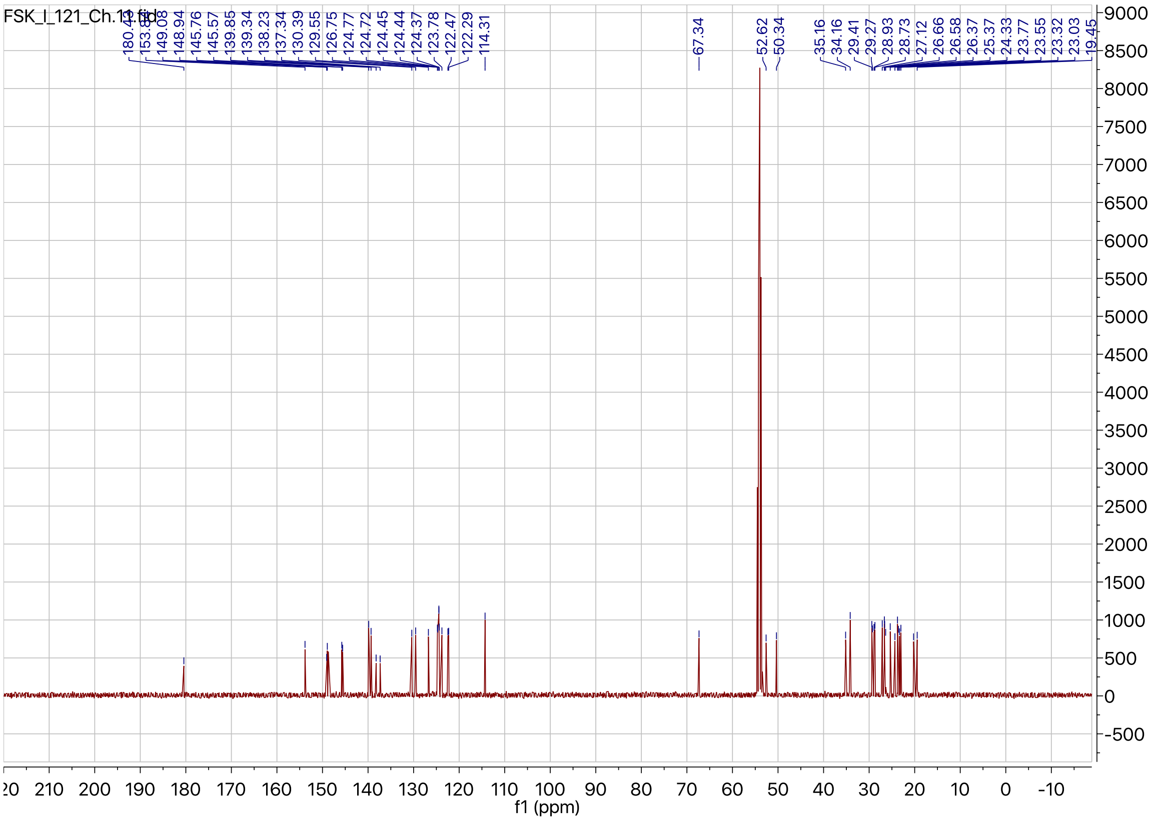

**Figure S21.** $^{1}H$ and $^{13}C$ NMR spectra of (IPr)[2-(1-(*N*-piperidyl)hex-5-en-1-yl)phenyl]nickel(II)chloride (**17**) in $CD_2Cl_2$.

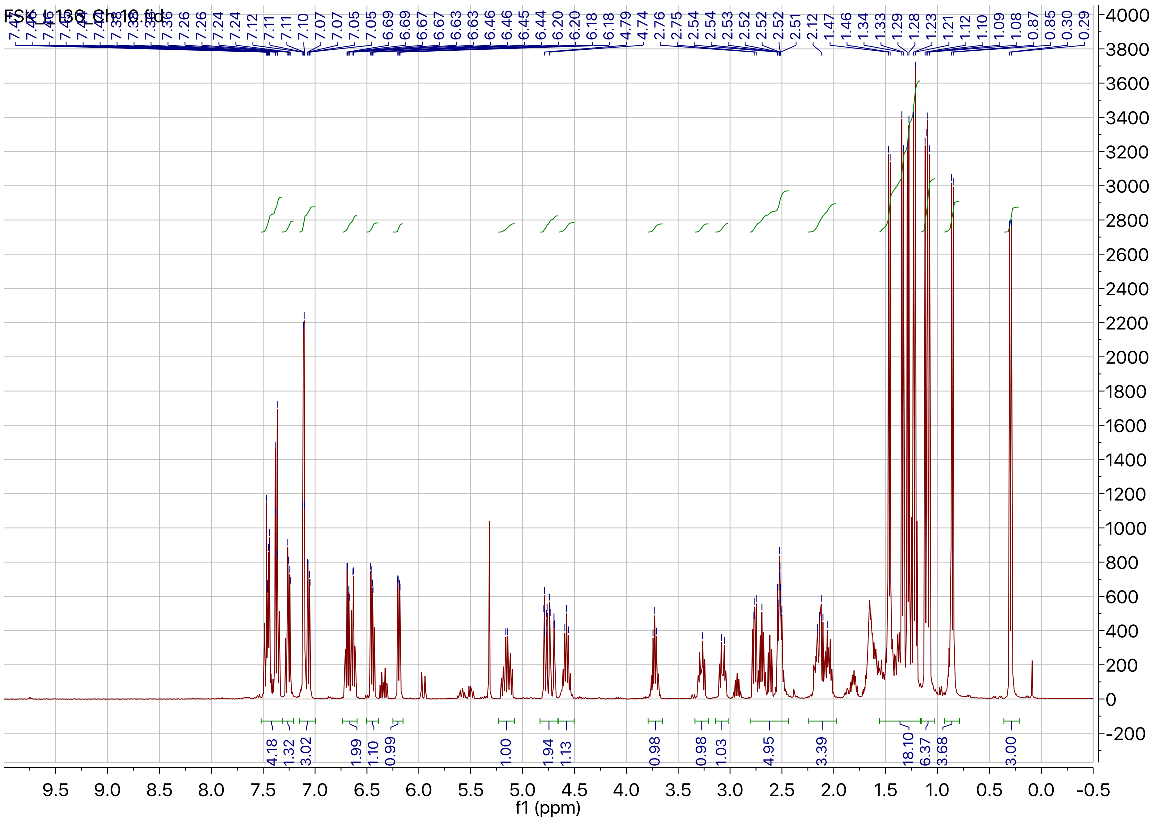


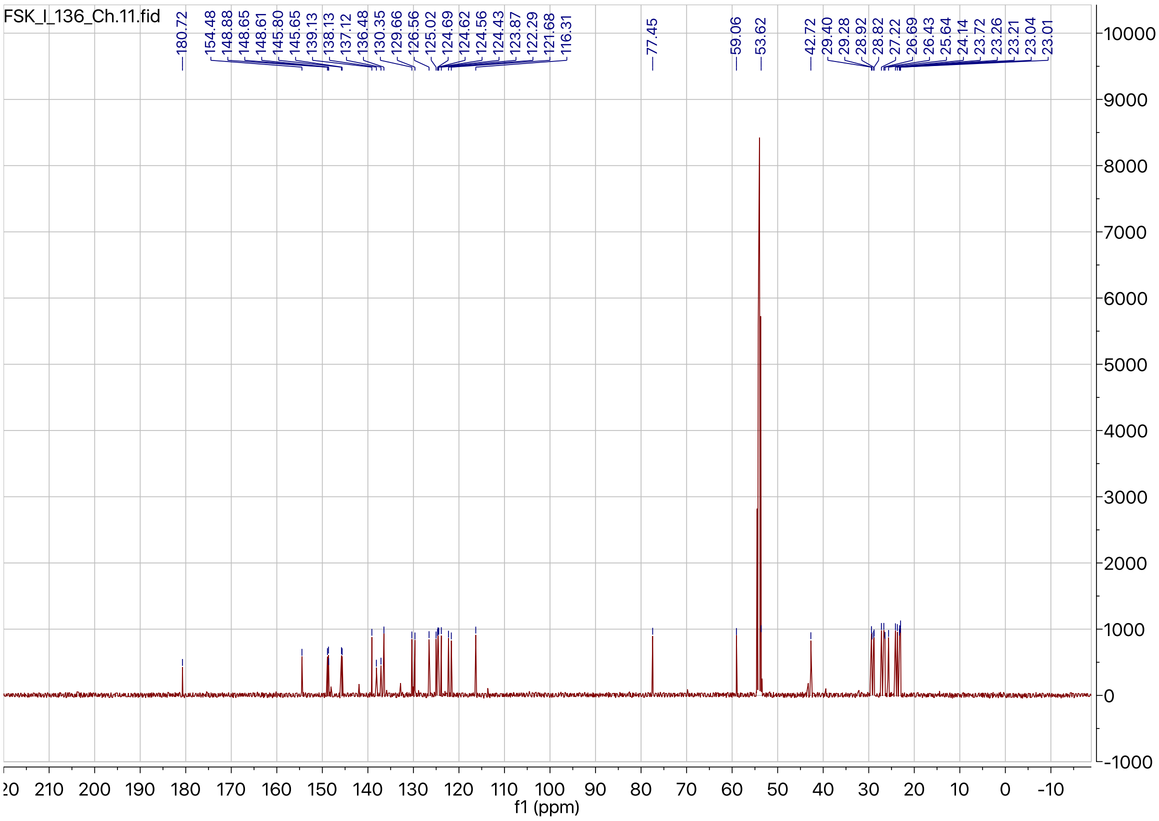

**Figure S22.** $^{1}H$ and $^{13}C$ NMR spectra of (IPr)[2-(1-(*N*-piperidyl)but-1-yl)phenyl]nickel(II)chloride (**18**) in $CD_2Cl_2$.

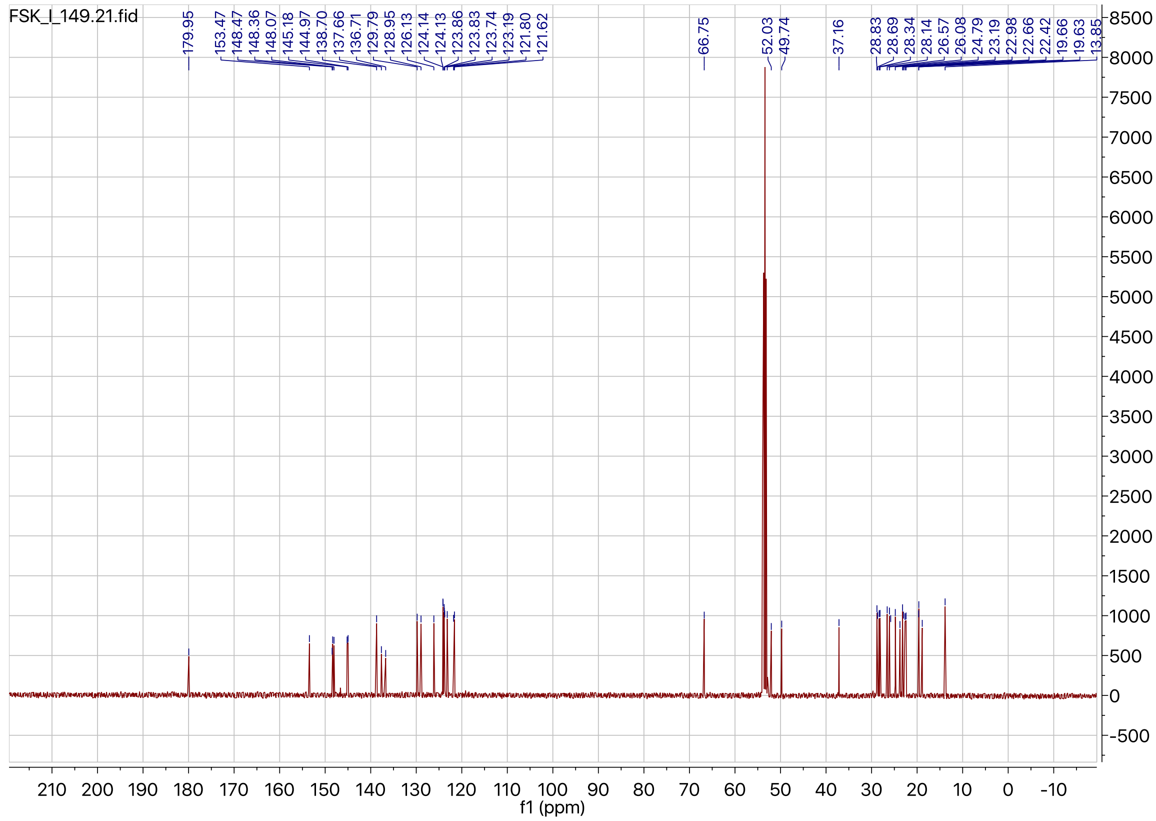


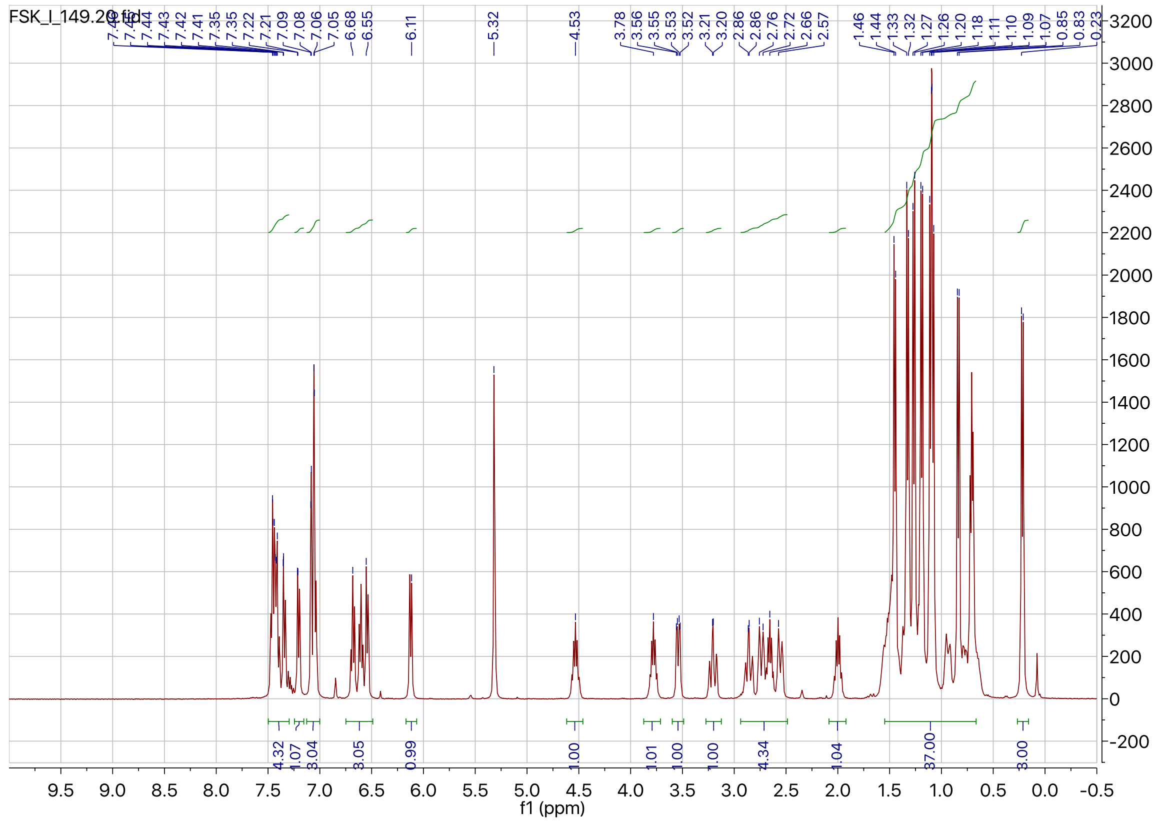

**Figure S23.** $^{1}H$ and $^{13}C$ NMR spectra of (SIPr)[2-(1-(*N*-piperidyl)but-3-en-1-yl)phenyl]nickel(II)chloride (**5b**) in $CD_2Cl_2$.

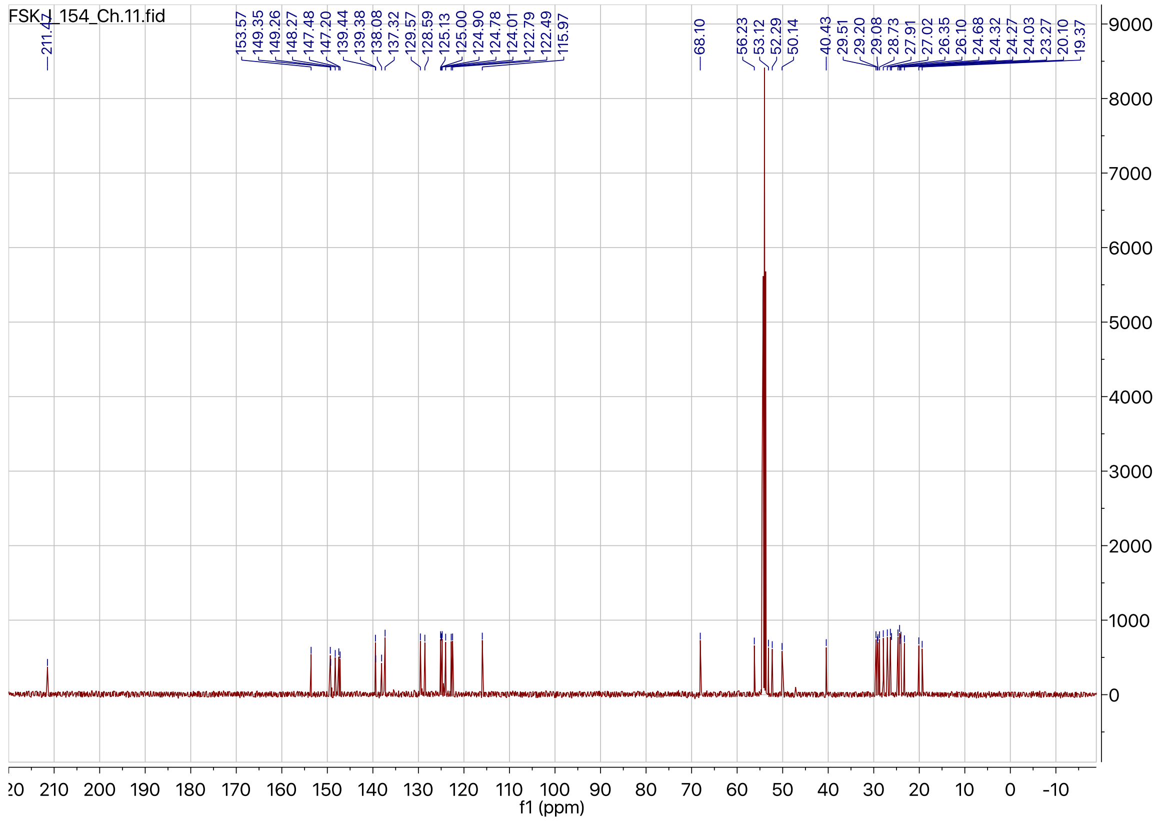

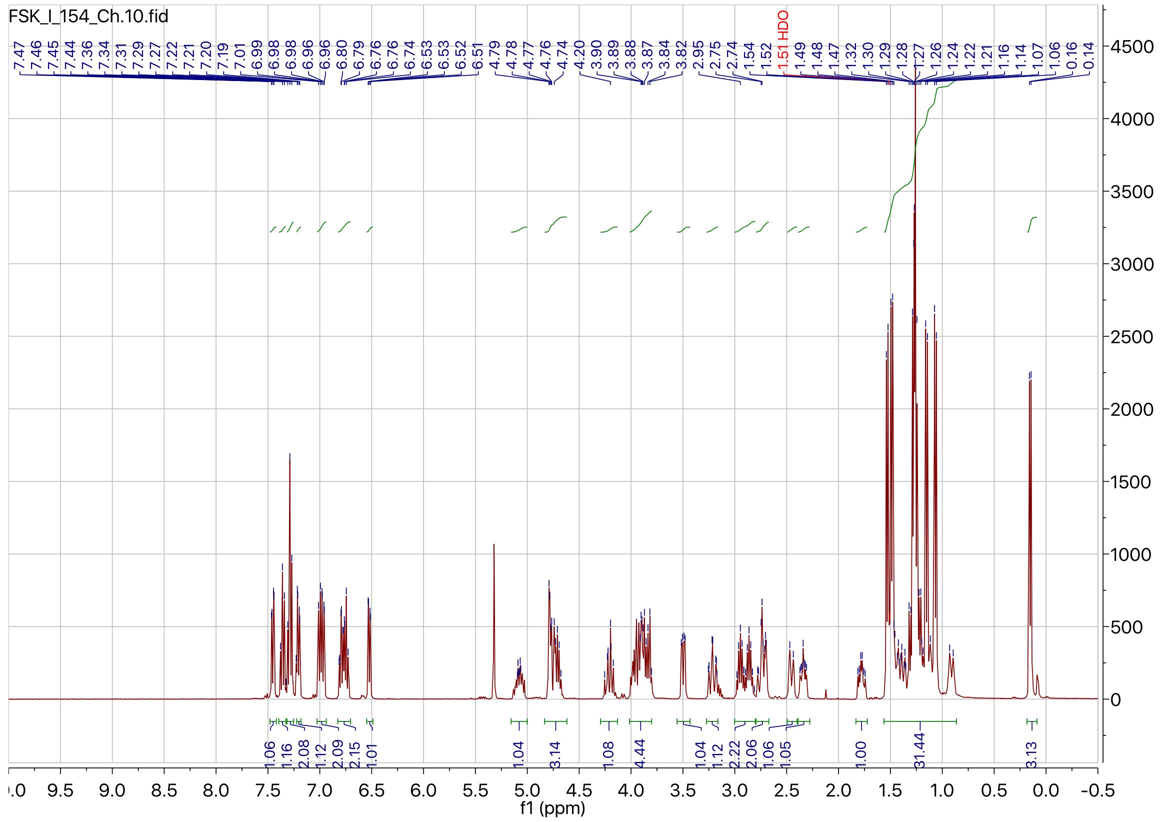

**Figure S24.** $^{1}H$ and $^{13}C$ NMR spectra of compound **6** in $CDCl_3$.

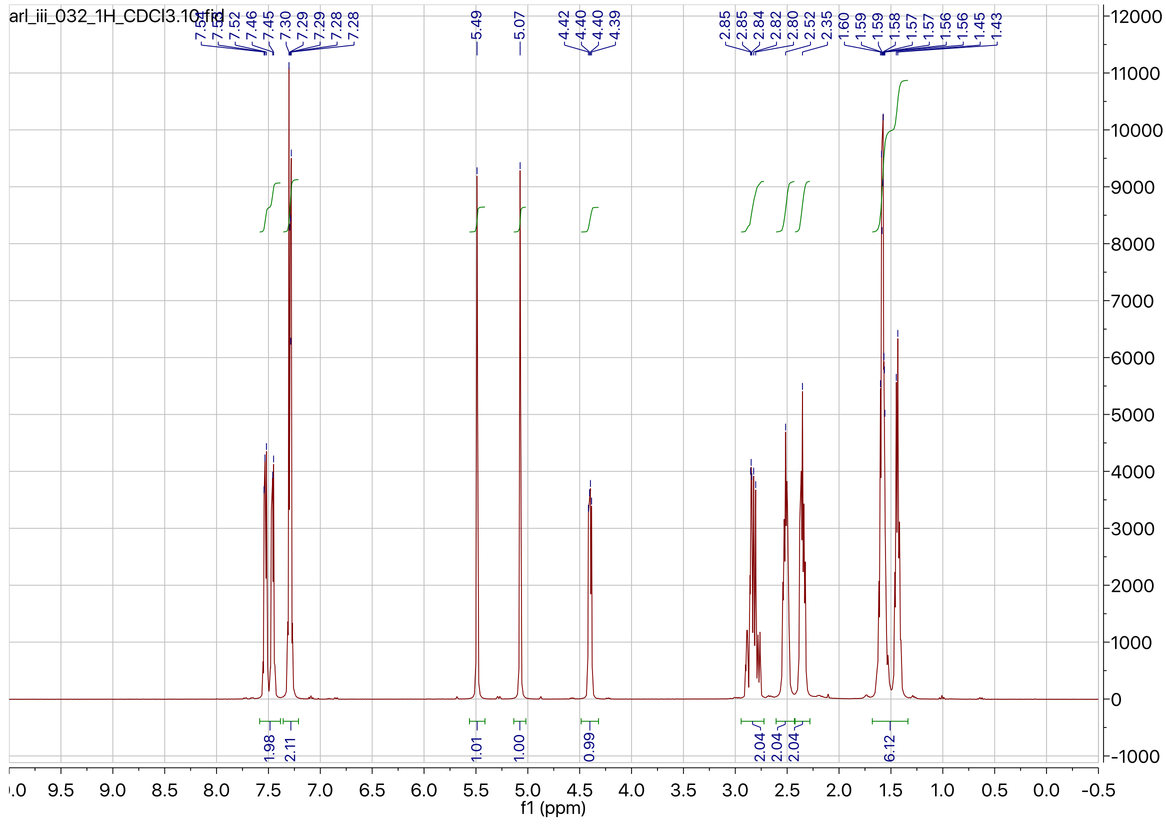


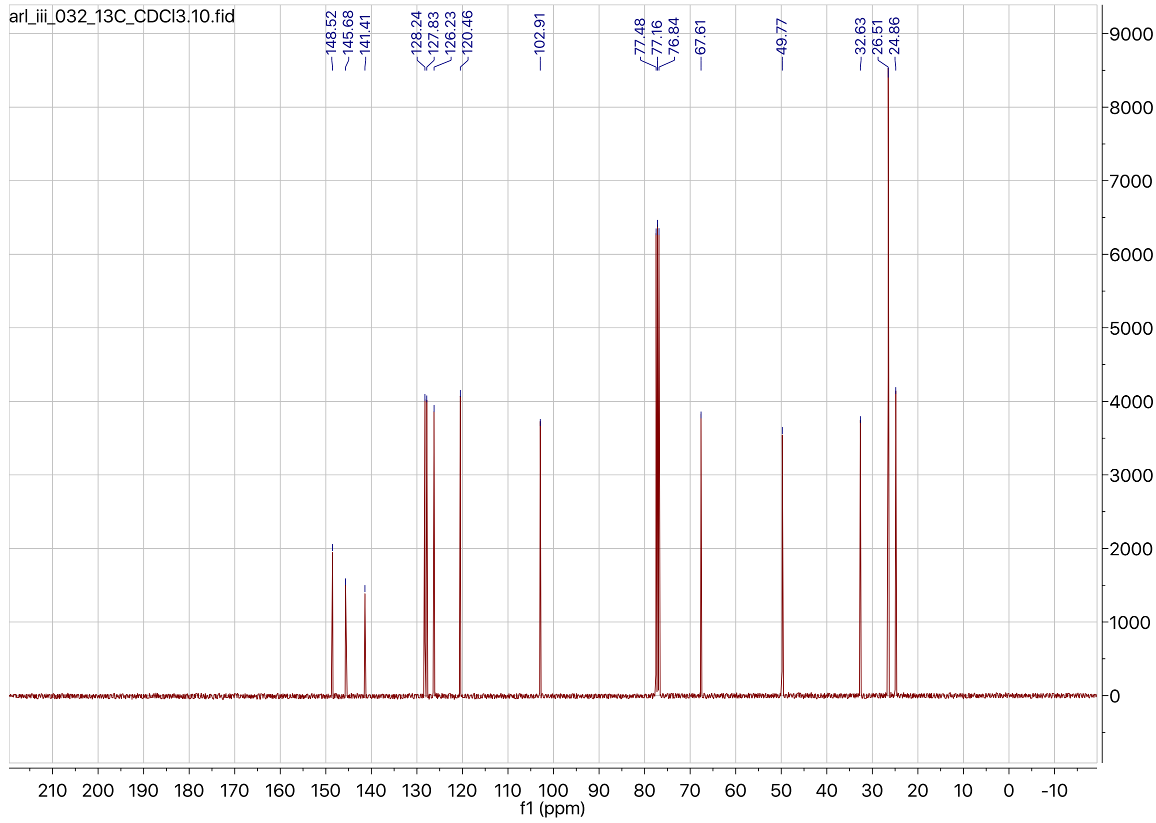

Supplement: Supplementary file 1 — Supplementary [file CCTC-10-2873-s001.pdf]
